# Interpreting, analysing and modelling COVID-19 mortality data

**DOI:** 10.1007/s11071-020-05966-z

**Published:** 2020-10-01

**Authors:** Didier Sornette, Euan Mearns, Michael Schatz, Ke Wu, Didier Darcet

**Affiliations:** 1 Tokyo Tech World Research Hub Initiative (WRHI), Institute of Innovative Research, Tokyo Institute of Technology, Yokohama, 226-8502 Japan; 2 Institute of Risk Analysis, Prediction and Management (Risks-X), Academy for Advanced Interdisciplinary Studies, Southern University of Science and Technology (SUSTech), Shenzhen, 518055 China; 3 Department of Management, Technology and Economics, ETH Zurich, Scheuchzerstrasse 7, 8092, Zurich, Switzerland; 4 Gavekal Intelligence Software, 75016 Paris, France

**Keywords:** COVID-19 epidemic, Mortality, Life expectancy, Stringency of confinement measures, Logistic equation, Outbreak progress

## Abstract

We present results on the mortality statistics of the COVID-19 epidemic in a number of countries. Our data analysis suggests classifying countries in five groups, (1) Western countries, (2) East Block, (3) developed Southeast Asian countries, (4) Northern Hemisphere developing countries and (5) Southern Hemisphere countries. Comparing the number of deaths per million inhabitants, a pattern emerges in which the Western countries exhibit the largest mortality rate. Furthermore, comparing the running cumulative death tolls as the same level of outbreak progress in different countries reveals several subgroups within the Western countries and further emphasises the difference between the five groups. Analysing the relationship between deaths per million and life expectancy in different countries, taken as a proxy of the preponderance of elderly people in the population, a main reason behind the relatively more severe COVID-19 epidemic in the Western countries is found to be their larger population of elderly people, with exceptions such as Norway and Japan, for which other factors seem to dominate. Our comparison between countries at the same level of outbreak progress allows us to identify and quantify a measure of efficiency of the level of stringency of confinement measures. We find that increasing the stringency from 20 to 60 decreases the death count by about 50 lives per million in a time window of 20  days. Finally, we perform logistic equation analyses of deaths as a means of tracking the dynamics of outbreaks in the “first wave” and estimating the associated ultimate mortality, using four different models to identify model error and robustness of results. This quantitative analysis allows us to assess the outbreak progress in different countries, differentiating between those that are at a quite advanced stage and close to the end of the epidemic from those that are still in the middle of it. This raises many questions in terms of organisation, preparedness, governance structure and so on.

**Electronic supplementary material:**

The online version of this article (10.1007/s11071-020-05966-z) contains supplementary material, which is available to authorized users.

## Introduction

Since first identified in December 2019 in Wuhan, China, a novel coronavirus disease (COVID-19) caused by the SARS-CoV-2 virus has been spreading in China in Jan–Feb 2020, and then was declared a global pandemic on March 11, 2020, by the World Health Organisation (WHO). As of 24 April 2020 when the first version of this paper was finalised, despite various starting times of the outbreak among different countries, more than 2.7 million cases of COVID-19 have been reported worldwide with 190K acknowledged deaths. As of 19th July 2020 when the revised version of this paper was finalised, according to the European Centre for Disease Prevention and Control (in accordance with the applied case definitions and testing strategies in the affected countries), 14.3 million cases of COVID-19 have been reported, including approximately 602,000 deaths.

An immediate qualitative observation of the current epidemic is the wide range of mortality outcomes among various countries and regions, suggesting a number of entangled factors affecting the statistics. It was clear from the start that mortality data were impacted by two key variables, namely the size of population and the degree of progression of the outbreak. We developed methodology to normalise for both of these variables that we describe below. For example, Japan, South Korea and Singapore seemed to have much lower death rates compared to West European economic peers. Hubei Province in China seemed to have much higher mortality than all other Chinese provinces. Germany and Norway seemed to be performing a lot better than West European peers. Eastern Europe seemed to be performing much better than the West. And Mexico seemed to perform better than the neighbouring USA. Why was it that poorer countries seemed to be performing so much better than the rich countries of the OECD with their high performance health services?

In this paper, we try to untangle some of these questions by dissecting mortality statistics in details. In Sect. 2, we demonstrate how mortality statistics are generated and discuss the potential reliability and consistency issues. In Sect. 3, we transform the mortality data to partly account for the normalisation problem across country and time, and then analyse the two key variables: age distribution and lockdown strategies. In Sect. 4, we provide a top-down modelling approach to analyse the current stage of the epidemic in different countries and project future scenarios. In Sect. 5, we discuss potential implications of our results.

## Mortality data: understanding its nature and biases

In order to understand the mortality data of COVID-19, it is vital to first analyse the characteristics of the disease and how it leads to a death. SARS-CoV-2 is a positive-sense single-stranded RNA virus, with a single linear RNA segment, belonging to the broad family of viruses known as coronaviruses. It is unique among known betacoronaviruses in its incorporation of a polybasic cleavage site, a characteristic known to increase pathogenicity and transmissibility in other viruses [1–3]. Common symptoms include fever, cough and shortness of breath. Other symptoms may include fatigue, muscle pain, diarrhoea, sore throat, loss of smell and abdominal pain [4]. The elderly and those with underlying medical problems like chronic bronchitis, emphysema, heart disease or diabetes are more likely to develop serious illness [5–7]. There has been an increasing number of reports of COVID-19 outbreaks in long-term care homes across Europe with high associated mortality, highlighting the extreme vulnerability of the elderly in this setting [8]. It is important to stress the characteristics of infections by SARS-CoV-2, which is mainly dangerous for the elderly and persons with co-morbidity, in contrast with many previous epidemics (including the Spanish flu of 1918–19, the Asian flu of 1957) for which a large proportion of deaths were teenagers and young people [9–11].

The five stages of COVID-19 progression as we understand them are:

- Stage 1 (asymptomatic or presymptomatic): Asymptomatic infection with SARS-CoV-2 where the infected person does not know they have the disease but could probably transmit it to others [12–14]. It is this feature of SARS-CoV-2 that makes it particularly difficult to contain. A recent modelling study suggested that asymptomatic individuals might be major drivers for the growth of the COVID-19 pandemic [15]. It is possible that asymptomatic cases may never develop symptoms [16], but if they do, the time between exposure to COVID-19 and the onset of symptoms is commonly around five to six days but can range from 1 to 14 days [17–19]. We note however that the WHO did not accept the claim of asymptomatic infections and even challenges this claim on its website; see also the points raised by Beda M Stadler, former director of the Institute for Immunology at the University of Bern [20] against this claim of “healthy sicks”.
- Stage 2 (mild): An unknown number of persons progress from Stage 1 to develop symptoms. Based on data from China, the WHO estimate that 75% asymptomatic cases continue to develop symptoms after testing positive, 80% of laboratory confirmed patients have had mild to moderate disease and the median time from onset to clinical recovery for mild cases is approximately 2 weeks [21]. The two key symptoms are a mild fever accompanied by a chesty cough. The severity of these symptoms varies widely from case to case.
- Stage 3 (moderate): A small but unknown fraction of those who develop symptoms do not recover and begin to develop more serious pathological conditions. Many who contract this secondary infection remain at home and manage to recover.
- Stage 4 (severe): A small but unknown fraction from Stage 3 become more seriously ill and develop respiratory distress, requiring admission to hospital. In China and the USA, hospitalisation has occurred in 10.6% and 20.7 to 31.4% of cases reported, respectively [8]. Lungs lose their ability to absorb sufficient amount of oxygen. The administration of oxygen buys the patient time and aids recovery. Autopsies have revealed severe violation of microcirculation in the lungs in a number of dead patients [22].
- Stage 5 (critical): A small but unknown fraction do not recover at Stage 4, become critically ill, and are admitted to an intensive care unit where many are placed on invasive mechanical ventilation. The European Centre for Disease Prevention and Control (ECDC) estimates that 7% hospitalised cases are admitted to intensive care units (ICU) based on data from 13 countries [8]. Median length of stay in ICU has been reported to be around seven days for survivors and eight days for non-survivors, though evidence is still limited [23, 24]. At this stage, an unknown fraction dies while the remainder recover with potential lung damage and viral damage to a wide range of organs including kidneys, liver and heart [22].

Stage 1 makes COVID-19 a particularly infectious disease since an infected and contagious person may pass the disease on to others without even knowing they had it. Compared to seasonal influenza with a basic reproduction number $R_0 \sim$ 1.1 to 2.0 [25, 26], COVID-19 is estimated to have a much higher $R_0 \sim$ from 1.4 to 6.5 [17, 19, 27–29]. In fact, there is no unique number since transmission is heavily dependent upon population density and structure as well as the biological characteristics. Large cities with underground trains will have higher $R_0$ than remote rural areas. Family tradition may also play a role since this is mainly a disease of the very old. If the family tradition is to have grandparents in the family home (Italy and Iran), or staying in care homes, then there is a higher possibility of the elderly getting infected.

Before tackling these entangled factors, we need to first understand what is behind the numbers. Usually, we have an absolute measure and relative measure of mortality statistics. For the absolute measure, i.e. the number of COVID-19 deaths, it is important to acknowledge different standards of death reporting system among countries. The WHO guidelines mandated that the death be recorded as COVID-19 if it is a probable or confirmed COVID-19 case, unless there is a clear alternative cause of death that cannot be related to COVID-19 disease [30].

**Fig. 1:**
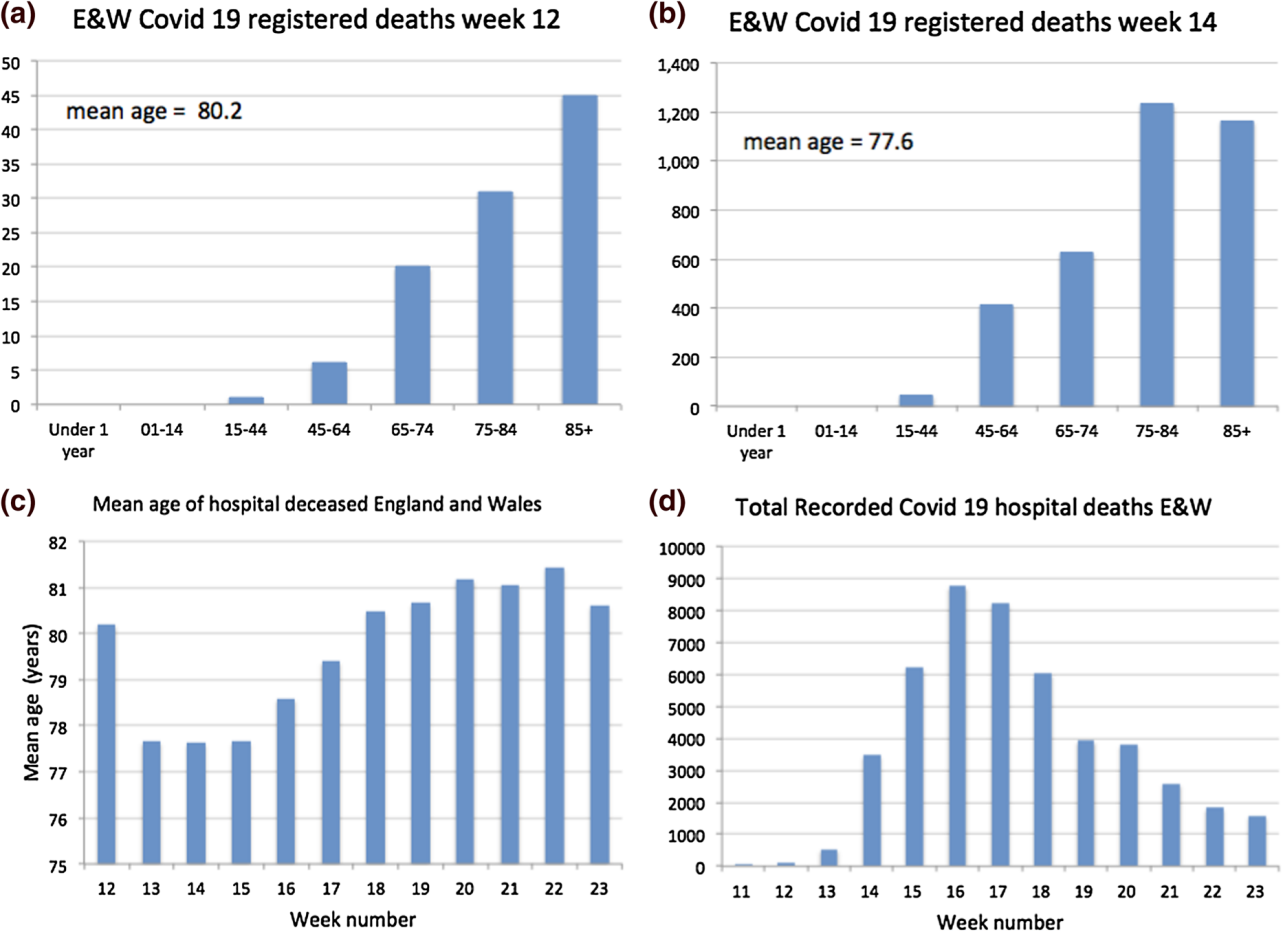
Mortality statistics for England and Wales for week 12 ending 20 March (**a**), for week 14 (**b**), mean recorded age of hospital decease week 12–23 (**c**) and total recorded hospital deaths in week 11–23 (**d**)

As illustrations of the heterogeneity of reporting standards, it is useful to review the case of the UK and of New York. The UK Office of National Statistics (ONS) began publishing data on the number of UK deaths from COVID-19 that began to occur during week 11, i.e. the week ending 13 March 2020. Figure 1 shows details on the UK reported mortality in hospitals. (A) shows the age profile of hospital deaths from Covid-19 in England and Wales. Like other countries, the mortality profile shows that the disease was most lethal in the ageing 65+ cohorts and increased exponentially with age; (B) in week 14, the age profile had changed reflecting a change in policy where (1) elderly patients in hospitals were sent back to their care homes and (2) doctors became more selective sending very elderly Covid-19 patients from care homes to hospital since it was recognised that the survival of the very old was not good and hospital capacity was reserved for younger patients; (C) shows how the plunge in age of the hospital deceased was reversed in week 16 [the peak of mortality, see panel (D)], presumably because it was now recognised that hospital capacity was not over-stretched and an increasing number of elderly were admitted, many of whom died as testified by the statistics; (D) the profile of hospital deaths from Covid-19 in England and Wales showing the huge peaks of more than 8000 deaths in weeks 16 and 17. These huge peaks in part reflect failure to protect the most vulnerable from infection in care homes.

On April 14, New York’s mortality statistics included people who died at home without getting tested, or who died in nursing homes or at hospitals, but did not have a confirmed positive test result. The New York Times [31], The Economist [32] and The Financial Times [33] estimated there might be up to 100% more deaths not included in the current statistics in some countries based on an analysis of the excess deaths, although they did not correct for the significant short-term reporting lag in mortality data (see section A.3 in the “appendix” for a visualisation of this reporting delay). On April 17, authorities in Wuhan revised the local death toll upward by 50%.

Regarding the relative measure of mortality statistics, most media and reports only use case fatality rate (CFR), the number of deaths divided by the number of confirmed cases, to compare status of the epidemic among different countries. Let alone the reliability of the absolute number of deaths (the numerator mentioned above), the denominator—number of confirmed cases—is also subject to a number of biases. For example, China’s national health commission issued seven versions of a case definition for COVID-19 between 15 January and 3 March, and a recent study found each of the first four changes increased the proportion of cases detected and counted by between 2.8 and 7.1 times [34]. Furthermore, the number of cases is usually on the basis of testing, which is biased towards severe cases in some countries, health care staff in others (the UK) while towards a larger group in some other countries implementing massive testing programs, such as South Korea and Iceland. The testing protocols and accuracy may also have a large impact on the results.

Relating positive test results to real levels of infection is also subject to a large number of biases. It is important to note that the real number of infections is far higher than those recorded in positive tests since only a tiny fraction of any population has been tested. This relates to another concept: Infection Fatality Rate (number of deaths divided by total infections including asymptomatic cases). The commonly cited death rates for seasonal flu of  0.1% to 0.2% are usually reported in terms of a version of the CFR (deaths among the population who have visible symptoms of the disease), while several recent studies on seroprevalence of antibodies to SARS-CoV-2 in the general population [35, 36] have used IFR. These IFR cannot be directly compared with the CFR of seasonal flu. If, say, 50% of the infected population is asymptomatic, this implies that CFR $= 2$ IFR.

It is not realistic to wait for all the reliable statistics before we start to model and understand the progression of the COVID-19 epidemic. In the following section, we will use the existing statistics with appropriate transformation to extract some information about the status of the epidemic stage in different countries, keeping in mind all the caveats mentioned above and in the next subsection when drawing conclusions.

## Analysing key factors of international mortality rates

### Caveats

There are two main time series available for a wide range of countries, namely number of daily confirmed cases and the daily number of deaths. The former has the advantage of being a leading indicator but suffers from selective testing, time-dependent and often unknown number of tests and testing policies that vary significantly across countries. Mortality data, on the other hand, are a lagging indicator, which is much more reliable but, as discussed above, is still subject to (i) a lack of comparability due to varying times of outbreak of the epidemic throughout the world, (ii) a significant reporting lag and (iii) under-/overreporting (discussed in the previous section).

In this section, we deal with point (i), which allows us to identify essential drivers of COVID-19 mortality across countries. In this analysis, we assume that (ii) is approximately constant across the countries studied here, while (iii) has to be analysed on a case-by-case basis and is partially^1^ corrected for in Figs. 3 and 5 below. While the tools employed in this section are those of simple data analysis, in a dynamic and complex system such as the evolution of an epidemic, one should try to carefully understand and exploit the data along several dimensions before applying more sophisticated models. Analysing the most influential explanatory factors allows us to identify anomalies and put our prediction results of Sect. 4 in perspective.

Below, we use mortality data from Johns Hopkins University Centre for Systems Science and Engineering [37].

### Transforming mortality data for cross-country comparison

#### Population normalised death rates and rough geographic grouping

**Fig. 2:**
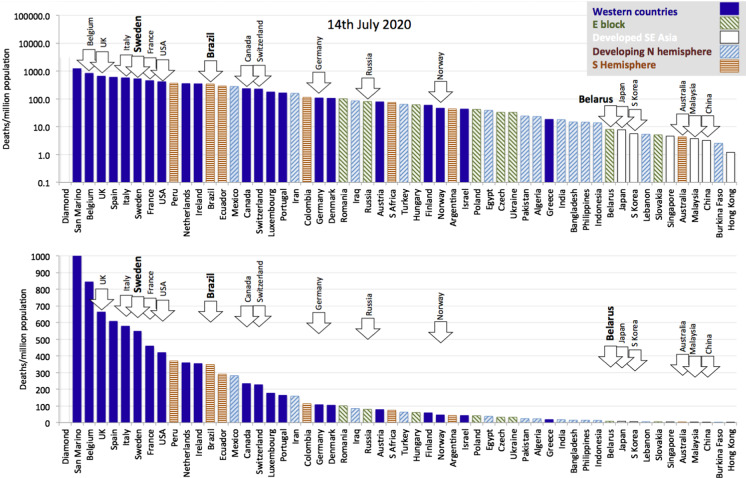
Deaths per million population for 55 countries selected to represent five main socioeconomic and geo-political groups from around the world. Sweden, Brazil and Belarus are highlighted as countries that have pursued a policy of no lockdown. The two graphs differ only in the logarithmic versus linear scale of their vertical axis, allowing us to put in perspective the relative death toll across different countries

To allow for a suitable comparison across countries, we normalise for population size and simply reduce mortality statistics to deaths per million population (deaths/mil). Figure 2 presents population normalised deaths (as of 15 July 2020) across a wide range of countries, which suggests a grouping by geographical factors into

1. Western countries (West Europe and North America) tend to have higher mortality rates and concentrate towards the left in Fig. 2;
2. East block countries tend to occupy the middle ground;
3. Developed SE Asian countries have extremely low mortality rates and are concentrated to the right of the distribution;
4. Developing Northern Hemisphere tends to have low mortality rates and is spread between the middle ground and the right of the distribution;
5. Southern Hemisphere countries, which may have initially benefited from late summer^2^ but have evolved since April, tending to occupy the middle ground trending towards the left with higher and mounting death tolls.

In general terms, the epidemic was halted early in a number of Western countries that have tended to move to the right of the distribution (e.g. Norway, Finland and Austria), and it arrived late in Latin America where Latin American countries are now moving towards the left. As mentioned above, these kinds of comparisons suffer from the fact that the epidemic evolves through highly clustered outbreaks and reaches countries at different times. In the next section, we propose a method to normalise for this variability. There are some notable exceptions to the general distribution. For example, Australia and New Zealand (not shown) are southern hemisphere outliers lying far to the right of the distribution. Australia and New Zealand are special cases being globally isolated islands with low population density that (so far) seem to have managed a highly efficient Southeast Asian style response.

#### Quantitative comparison by defining a suitable reference time

To normalise cumulative mortality trends to the same stage of outbreak, we suggest to align countries once they have reached a certain number of deaths per million (denoted deaths/mil). A larger value of deaths/mil will be more robust towards noise in the early reporting of mortality. On the other hand, if this reference time is too large, it will contain information on country-specific growth rates, which we want to avoid in our analysis below. As discussed in detail in section A.1, we choose 1 death/mil, the largest value that does not lead to a significant correlation of growth rates before and after the reference time. Below we refer to the respective date of alignment as the **datum**, representing the (country-dependent) date where 1 death/mil is reached.

**Fig. 3:**
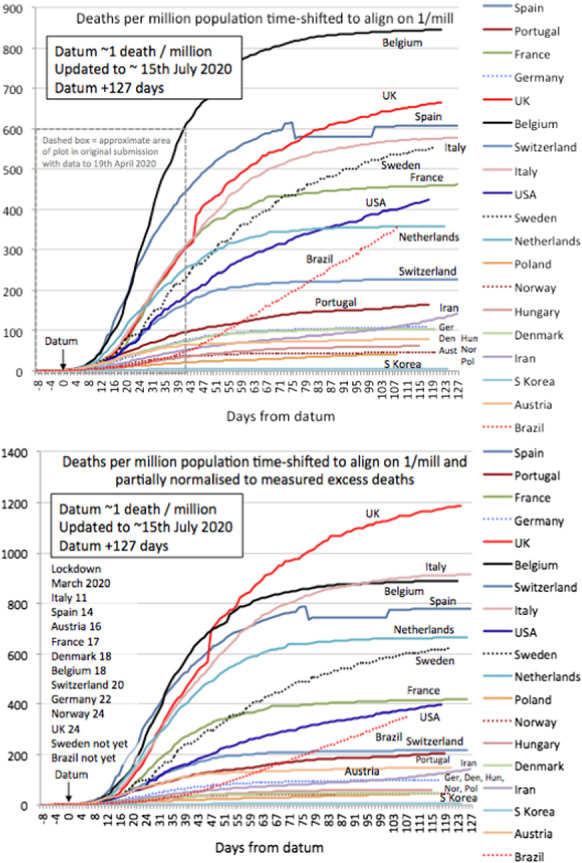
Cumulative mortality curves for selected countries. The datum marks the point where each country reached 1/mil deaths as explained in the text. Lower panel is corrected for potential underreporting according to [33], see text for details. The dashed box in the upper panel represents the data reported in an earlier version of this paper

Figure 3 illustrates this time shift for 18 selected countries. We have included a normalisation for reporting standard using excess mortality statistics as documented by the Financial Times [33].^3^ Note that these numbers (just like officially reported data) should be taken with a grain of salt, as these calculation are influenced by reporting delay, statistical anomalies in expected (“normal”) mortality due to past flu seasons and are likely to change significantly in time. Note that, even within Western countries, the spread in these curves shows a wide range in mortality outcomes, which we discuss in Sect. 3.3.2 below. The trajectories presented in Fig. 3 allow for a comparison of the early stages of the outbreak, a country’s health care performance will ultimately be judged on the final count of deaths per mil. In this respect, we discuss our logistic-based predictions and their performance in Sect. 4.

### Quantifying key factors of COVID-19 mortality

#### Age dependence and life expectancy

For most, the SARS-CoV-2 infection goes unnoticed (stage 1), for some, it leads to mild symptoms (stage 2). For a tiny minority of vulnerable people, it develops into a lethal disease (stages 4– 5). The relatively high $R_0$ produces a flood of hospital admissions giving an amplified sense of seriousness at the whole population level.

**Fig. 4:**
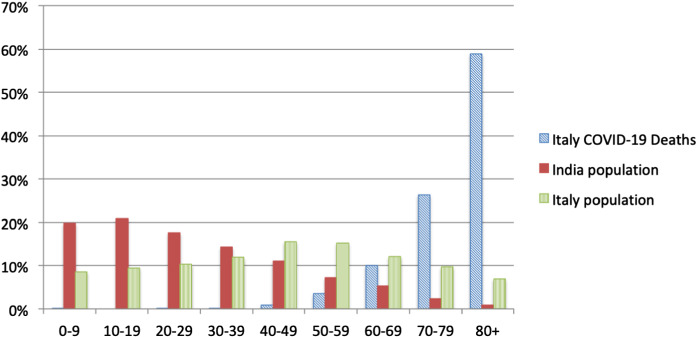
Mortality statistics for Italy until July 12 (Median age: 82 years) and the population structure of Italy and India

**Fig. 5:**
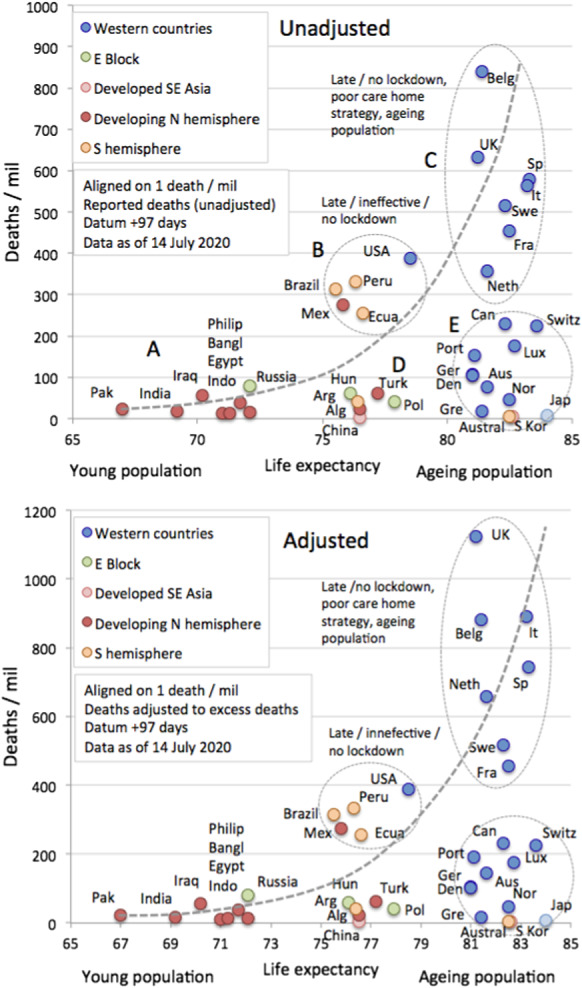
Cross plot showing the relationship between life expectancy and mortality expressed as deaths/mil. As reference time, we use **datum** = 1 death/mil and calculate deaths in an interval of 97 days. Lower panel is corrected for potential underreporting according to [33], see Sect. 3.2.2 for details

As noted in [23, 40] and evident from country-specific mortality statistics, there is clear evidence of those most susceptible to progression to Stage 5. Figure 4 shows the total number of deaths as recorded in Italy and the population structure of India. Mortality is highest in the older cohorts, with a median age of 80 years, while India has a small number of the very old in its population. This observation informs the hypothesis that COVID-19, a disease impacting mostly the old and already sick, is most marked in countries that have the largest quantities of very old people in their populations. We use life expectancy [41] as a readily available proxy for population structure and stratification. Figure 5 shows a cross plot for 38 countries of deaths/mil versus life expectancy. As reference time, we use **datum** = 1 death/mil and calculate deaths in an interval of 97 days. Certain countries, e.g. Japan, Norway and Switzerland may have managed to override the disadvantage of ageing populations with efficient intervention measures (group E). Similar results hold for countries in group D as compared to group B. The countries in group A seem to have relatively low mortality despite significant outbreaks, which we are arguing may be due to the paucity of old vulnerable targets in their populations.

**Fig. 6:**
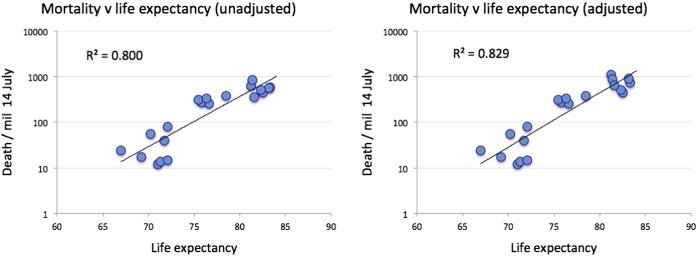
Regressions of the data shown in figure 5 for groups A–C using an exponential fit

Figure 6 shows the data for groups A-C in figure 5 plotted on log scale with exponential fit.

Based on the trends we identified here, we are inclined to draw the following conclusions (mind the special cases of Australia, South Korea and Japan identified above). We hypothesise that a key driver behind the large COVID-19 epidemic in the Western countries is because these rich countries have spent large amounts on healthcare with a focus on extending the lives of the elderly, through a range of medical and pharmaceutical interventions. Moreover, if there is a relation of climate/temperature to the outbreak severity of COVID-19, it is rather weak and does not seem significant as compared to the demographic structure. Finally, there is a substantial variation even within the Western countries that is unexplained by (minor) demographic, climatic or geographical differences. We analyse these countries in the next section.

#### Lockdown policies and mortality in Europe and the USA

As outlined above, looking at population, normalised mortality statistics allows us to compare the progression of the epidemic across countries: in particular, we can quantify what is the shift in time needed to best make comparable the mortality curves of various countries. The analysis here can be understood as top-down complementary to studies such as [42–45], which are subject to additional assumptions and do not allow for a straightforward comparison across countries. The value of this time-shifted dataset is to allow us, for instance, to analyse the potential efficiency of intervention and lockdown policies across a number of Western countries.

**Stringency index**

The authors in [46] collect and update an exhaustive list of government responses to COVID-19. In particular, they calculate a *Stringency Index* as a daily time series of numbers in [0, 100], based on an average of the following policies:

1. School closure,
2. Workplace closure,
3. Canceling public events,
4. Closing public transport,
5. Public info campaigns,
6. Restrictions on internal movement,
7. International travel controls.

For a detailed explanation and weighting of various policies, we refer to [46]. Note that, this index does not cover personal non-pharmaceutical interventions such as increased personal hygiene or voluntary social distancing.

**Cumulative mortality and lockdown**

**Fig. 7:**
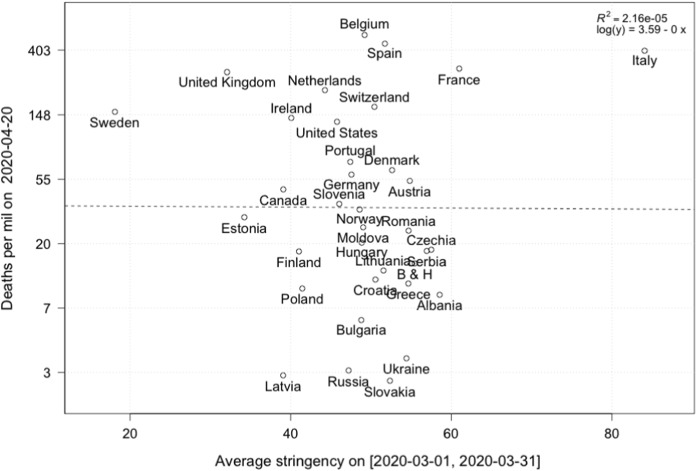
Cumulative deaths (log scale) on 2020/04/20 and average stringency of lockdown over a time horizon [2020/03/01,2020/03/26]

A first pass examination of this data is to look at cumulative mortality in a range of countries and their relation to the stringency index. For this, we fix a date $t=2020/04/20$ and compare the cumulative deaths per 1 million population on this date. Using the results from [47, 48], who report an average time from infection to death of roughly 20  days,^4^ we calculate the average Stringency Index on a time interval $[2020/03/01,t-20]$ and plot against total deaths at *t*, see Fig. 7. Here, the initial time point is chosen ad hoc. It would seem that the stringency index has little influence on the number of deaths in a country. However, as we have discussed above, the epidemic arrived in various countries at different times, so one needs to carefully account for this time lag.

To get a more robust result on efficiency of lockdown strategies, we conduct the following steps to transform the data, as recorded in Table 1:

1. For each country, we choose the beginning of the epidemic to be at
  1 $$\begin{aligned} \text {datum} {=} \text {1 deaths per million} \end{aligned}$$
  and record for each country the time where this number was reached (to be precise, we choose the day where the reported number was closest to 1 deaths per million)
2. Next, we choose some time frame *T*, restricted by the country being the latest to have reached 1 deaths per million. In our example below, we set $T=20$ days.
3. For each country, we calculate the number of deaths in a time interval $[\text {datum}, \text {datum} + T]$.
4. Finally, we calculate the average stringency index in a time interval shifted backwards by 20 days to account for the average time of infection to death [47, 48].

**Fig. 8:**
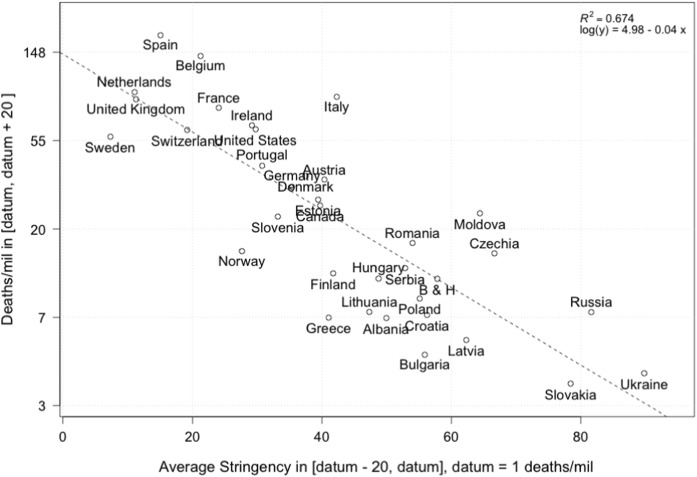
Cumulative deaths (log scale) in an interval aligned at 1 deaths per million and lasting 20 days plotted and regressed against average stringency index

Figure 8 shows the number of cumulative deaths with respect to lockdown strategies in European countries, using the methodology outlined above. We note a rather convincing negative correlation between stringency during the beginning of the epidemic and the logarithm of the number of deaths in a subsequent time interval. To complement Fig. 8, we present a country-specific analysis in “appendix A.2”, which shows a significant reduction in mortality growth rates around the time we expect to see an impact of intervention measures.

**Table 1:** First column: country names; second column: datum defined as the date of reaching 1 death per million (**datum**); third column: cumulative deaths at datum; fourth column: cumulative deaths in a time interval of size $T=20$; fifth column: average stringency index on a time interval shifted by $-20$ days

Country	Datum	Deaths on datum	Deaths in [datum.datum+20]	Average stringency
Albania	2020-03-22	0.7	7.3	50.0
Austria	2020-03-21	0.9	35.2	40.4
Belgium	2020-03-17	0.9	141.9	21.3
Bosnia & Herzegovina	2020-03-26	0.9	11.4	57.8
Bulgaria	2020-03-28	1.0	4.8	55.9
Canada	2020-03-26	1.0	26 2	39.7
Croatia	2020-03-28	1.2	7.6	56.3
Czechia	2020-03-28	1.0	15.2	66.7
Denmark	2020-03-19	1.0	35.6	37.5
Estonia	2020-03-28	0.8	28.0	39.4
Finland	2020-03-26	0.9	12.1	41.8
France	2020-03-13	1.2	79.3	24.1
Germany	2020-03-21	1.0	324	35.4
Greece	2020-03-21	1.2	7.4	41.0
Hungary	2020-03-25	1.0	11.5	48.8
Ireland	2020-03-22	0.8	64.9	29.2
Italy	2020-03-02	0.9	89.5	42.3
Latvia	2020-04-07	1.0	5.7	62.3
Lithuania	2020-03-24	0.7	7.9	47.4
Moldova	2020-03-30	0.7	24.0	64.4
Netherlands	2020-03-16	1.2	94 7	11.1
Norway	2020-03-18	1.1	15.6	27.6
Poland	2020-03-31	0.9	9.1	55.1
Portugal	2020-03-21	1.2	41.1	30.8
Romania	2020-03-25	0.9	17.2	54.0
Russia	2020-04-13	1.0	7.8	81.6
Serbia	2020-03-26	0.6	12.9	52.9
Slovakia	2020-04-16	1.1	3.5	78.4
Slovenia	2020-03-22	1.0	23.1	33.2
Spain	2020-03-11	1.2	179.7	15.1
Sweden	2020-03-18	1.0	57.1	7.3
Switzerland	2020-03-13	1.3	61.7	19.2
Ukraine	2020-04-07	1.0	3.9	89.8
United Kingdom	2020-03-16	1.0	87.3	11.3
United States	2020-03-20	1.1	62.1	29.3

We can thus take our analysis in the last section a step further to arrive at the following classification along two axes:

- To explain the vast differences in severity of the epidemic, which seems to have taken its most devastating course in Western developed countries, we noted that demographics is a key driver, with potential (but minor) geographic, cultural and climatic influences (Sects. 3.2.1 and 3.3.1), while
- within Europe, the (comparably) smaller differences in per-capita mortality can, to some extend, be explained by severity and timing of intervention measures (which could either be effective in itself or lead to increased awareness and personal non-pharmaceutical interventions).
  Some countries seem to have been hit exceptionally hard, which could potentially be related to an inadequate dealing with the spread of the epidemic in elderly care homes [49] and calls for a further investigation. In [50], the authors collect early evidence on the number of care home deaths.^5^ Recent information from the UK office for national office statistics (as documented in [50]) suggests a significant amount of uncounted deaths in care homes.

### A short discussion of other factors

In addition to the factors we have identified above, there have been a range of reports and articles that note other elements influencing mortality.

Co-morbidities have been identified as a significant factor for both older and younger COVID-19 patients [5–7, 53, 54], where coronary heart disease, cancer, diabetes and respiratory disorders like asthma were most common. Underlying health conditions reported among patients with COVID-19 and admitted to ICU include hypertension, diabetes, cardiovascular disease, chronic respiratory disease and immune compromised status. Data from the European Surveillance System (TESSy) for 21 European countries show that out of 5378 deaths, only 7.3% cases do not have underlying known health conditions [8]. Out of 1890 deaths in Italy, 3.7% of the sample presented no comorbidities, 14.4% with a single comorbidity, 21.2% with 2, and 60.7% with 3 or more. [55]. Additionally, obesity has been noted to have a high prevalence among patients with COVID-19 admitted to ICU [56–58], especially severe obesity (BMI $>35$). The effect of obesity was reported to be even more significant in younger patients [59]. A recent study [60] confirms many of these factors in a large study with COVID-19 patients in the UK.

Studies document that daily smokers have a lower likelihood of developing symptomatic or severe SARS-CoV-2 infection compared to the general population [61, 62]. But smokers, if infected, may have a greater risk of complications from COVID-19 disease and thus smoking is suspected to be a risk factor in developing serious illness from COVID-19, see the meta-study [63] and references therein.

Finally, a series of preprints has identified a potential link between BCG vaccination practice for Tuberculosis and COVID-19 mortality; see, for example, [64, 65]. However, accounting for confounding factors such as age or testing policies, this correlation seems to be rather weak [66, 67]; cf. a recent overview [68].

## Logistic projections of ultimate mortality in selected countries

### Models

Three ingredients are necessary for an epidemic to develop: 1) Source: pathogens and their reservoirs; 2) Susceptible persons with a way for the virus to enter the body; 3) Transmission: a path or mechanism by which viruses moved to other susceptible persons. Numerous mechanistic models have been utilised to study the COVID-19 epidemic, based on different assumptions about these three types of variables, including some that have broken down the population by age cohorts in particular to account for the age-dependence of epidemic characteristics, as well as adding the risk management policies such as lockdown strategies discussed in Sect. 3.3.2 (e.g. [42]).

Although mechanistic models are useful in understanding the effect of different factors on the transmission process, they are highly sensitive to the assumptions on the many often subtle microscopic processes. Giving an illusion of precision, mechanistic models are in general quite fragile and require an in-depth understanding of the dominating processes, which are likely to be missing in the middle and confusion of the pandemic, with often inconsistent and unreliable statistics and studies performed under strong time pressure. There is thus space for simpler and, we argue, more robust phenomenological models, which have low complexity but enjoy robustness. This is the power of coarse-graining, a well-known robust strategy to model complex system [69–71].

In this section, we thus use a basket of phenomenological models to describe the dynamics of the daily deaths and provide predictions for different future scenarios, as we have done for the confirmed cases in [72] and the real-time daily predictions in [73]. This simple and top-down approach can provide transparent interpretation and straightforward insights regarding the status of the epidemic and future scenarios of the outbreak, by simply calibrating the phenomenological models to the empirical reported data.

Usually, an epidemic follows an exponential or quasi-exponential growth at an early stage (following the law of proportional growth with multiplier equal to the basic reproduction number $R_0$), then the growth rate decays as fewer susceptible people are available to be infected and countermeasures are introduced to hinder the transmission of the virus. Therefore, an exponential or generalised exponential model can be used to describe the data at the early stage of an outbreak, which is intuitive and easy to calibrate. We use a generalised growth model (GGM) to describe the data in this stage:

2 $$\begin{aligned} \mathrm{d}C(t)/\mathrm{d}t=rC^p(t), \end{aligned}$$

where *C*(*t*) represents the cumulative number of cases (confirmed or deaths) at time *t*, $p\in [0,1]$ is an exponent that allows the model to capture different growth profiles including constant incidence ($p=0$), sub-exponential growth ($0<p<1$) and exponential growth ($p=1$). In the later case for which the solution is $C(t) =C_0 e^{rt}$, *r* is the growth rate. For $0<p<1$, the solution of equation (2) is $C(t) = C_0 (1+{rt \over A})^b$, where $b = {1\over 1-p}$ and $A= {C_0^{1-p} \over 1-p}$, so that *r* controls the characteristic time scale of the dynamics. The (quasi) exponential model essentially provides an upper bound for the future scenario by assuming that the outbreak continues to grow following the same process as in the past. However, an outbreak will slow down and reach its limit with decaying transmission rate in the end.

When the growth rate gradually decays and the daily incidence curve approaches its inflection point, the trajectory usually departs from a simple exponential growth, and a logistic-type model could have a better performance. We thus use three types of logistic models when the outbreak is leaving the early growth stage:

- Classical logistic growing model:
  3 $$\begin{aligned} \mathrm{d}C(t)/\mathrm{d}t=rC(t)\left( 1-{C(t) \over K}\right) \end{aligned}$$
- Generalised logistic model (GLM):
  4 $$\begin{aligned} \mathrm{d}C(t)/\mathrm{d}t=rC^p(t)\left( 1-{C(t) \over K}\right) \end{aligned}$$
- Generalised Richards model (GRM):
  5 $$\begin{aligned} \mathrm{d}C(t)/\mathrm{d}t=rC^p(t)\left( 1-\left( {C(t) \over K}\right) ^\alpha \right) \end{aligned}$$

The pure logistic equation (3) has the same number of parameters as the pure growth model (2), trading the exponent *p* for the final capacity *K*, which is the asymptotic total number of infections over the whole epidemic. In the generalised logistic model (4), the exponent $p\in [0,1]$ is introduced to capture different growth profiles, similar to in the generalised growth model (2). In the generalised Richards model, the exponent $\alpha$ is introduced to measure the deviation from the symmetric S-shaped dynamics of the simple logistic curve. The GRM recovers the original Richards model for $p=1$, and reduces to the classical logistic model for $\alpha =1$ and $p=1$. Therefore, the GRM is more pertinent when calibrating data from a region that has entered the after-peak stage, to better describe the after-peak trajectory that may have deviated from the classical logistic decay. However, this more flexible model leads to more unstable calibrations if used on early stage data.

### Methodology

Scenarios. As we have demonstrated in [72], logistic-type models tend to under-estimate the final capacity *K* and thus could serve as lower bounds for the future scenarios. We define the **positive scenario** as the model with the second lowest predicted final total deaths *K* among the three logistic models, and the **medium scenario** as the model with the highest predicted final total deaths among the three logistic models. Both positive and medium scenarios could underestimate largely the final capacity. The **negative scenario** is described by the generalised growth model, which should only describe the early stage of the epidemic outbreak and is therefore least reliable for countries in the more mature stage as it does not include a finite population capacity.

Calibration. For the calibration, we use the standard Levenberg-Marquardt algorithm to solve the non-linear least square optimisation. To estimate the uncertainty of our model estimates, we use a bootstrap approach with a negative binomial error structure $NB(\mu ,\sigma ^2)$, where $\mu$ and $\sigma ^2$ are the mean and variance of the distribution, estimated from the empirical data.

Data. The reported death data are from the European Centre for Disease Prevention and Control (ECDC)[8] up to July 17.

### Results

**Fig. 9:**
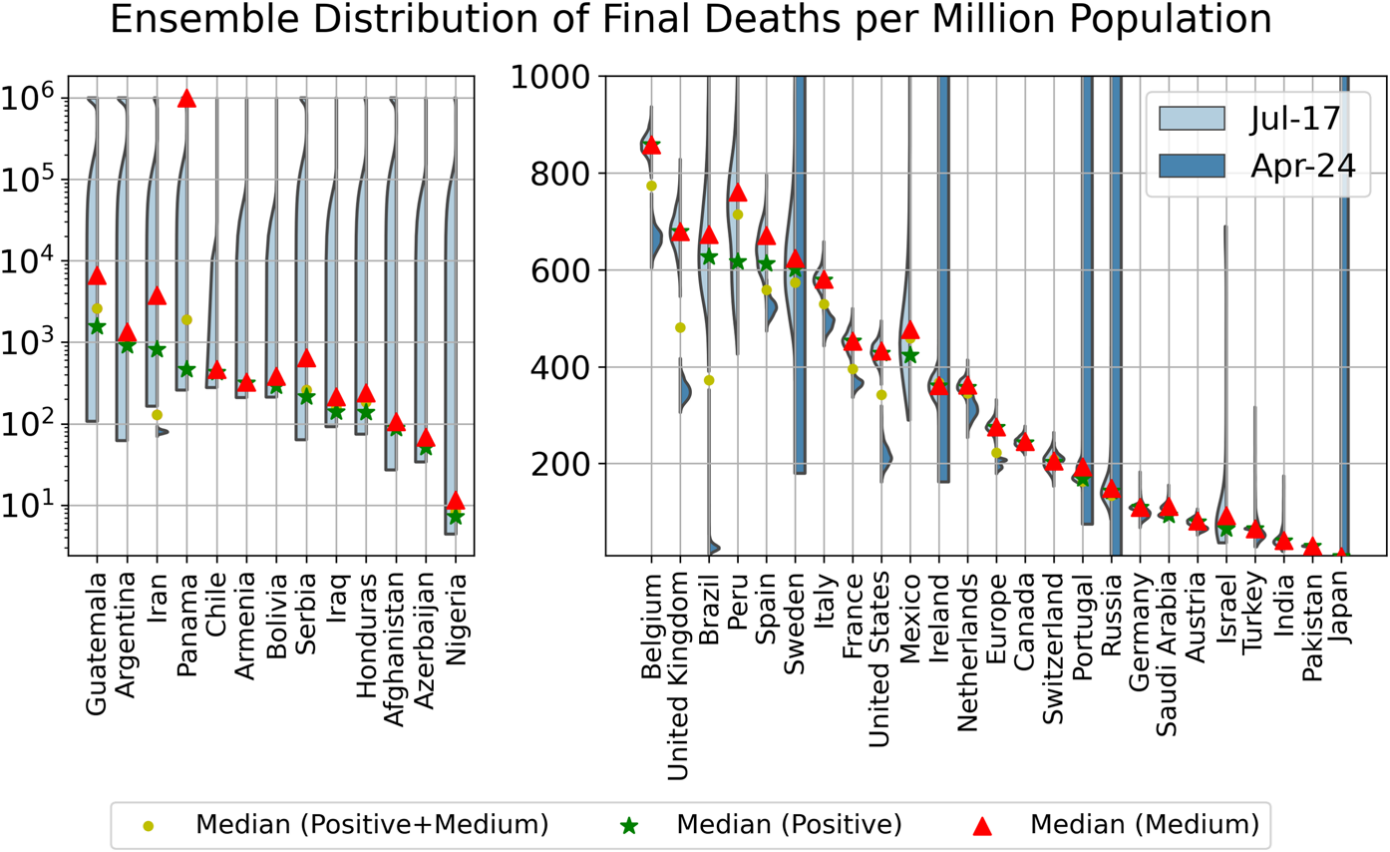
Violin plot of the distributions of the final total number of deaths per million derived by combining the distributions of the positive and medium scenarios. The left side of each violin in cyan is based on data up to July 17, while the right side of each violin in grey is based on data up to April 24, when the first version of this paper submitted. The left panel shows countries that have a distribution that has not converged, and the right panel presents countries that have converged. The model setup in the negative scenario does not incorporate a maximum saturation number and thus cannot be used. The yellow dots indicate the median prediction for the combined distribution, while the green and red dots indicate the median of the positive and of the medium scenarios, respectively

**Table 2:** As of July 17, 2020, current deaths per million population and estimated outbreak progress in positive and medium scenarios (today’s deaths divided by the estimated total final deaths in positive and medium scenario)

Country	Deaths per million population (Jul-17)	Outbreak progress in positive scenario	Outbreak progress in medium scenario
Italy	579	100.0%	100.0%
		(93.9%, 100.0%)	(97.3%, 100.0%)
Ireland	360	100.0%	100.0%
		(92.7%, 100.0%)	(92.9%, 100.0%)
Germany	110	100.0%	100.0%
		(92.7%, 100.0%)	(93.9%, 100.0%)
Austria	80	100.0%	100.0%
		(90.8%, 100.0%)	(91.2%, 100.0%)
Turkey	66	100.0%	100.0%
		(93.8%, 100.0%)	(97.0%, 100.0%)
Belgium	858	100.0%	99.9%
		(97.8%, 100.0%)	(95.8%, 100.0%)
United Kingdom	679	100.0%	99.9%
		(95.0%, 100.0%)	(91.5%, 100.0%)
Europe	274	100.0%	99.7%
		(97.1%, 100.0%)	(92.3%, 100.0%)
France	450	99.7%	99.4%
		(94.4%, 100.0%)	(94.8%, 100.0%)
Netherlands	356	100.0%	99.4%
		(93.9%, 100.0%)	(94.9%, 100.0%)
Spain	608	99.2%	99.2%
		(92.9%, 100.0%)	(93.0%, 100.0%)
United States	423	100.0%	98.9%
		(90.1%, 100.0%)	(92.5%, 100.0%)
Canada	238	100.0%	98.0%
		(94.1%, 100.0%)	(92.1%, 100.0%)
Switzerland	198	97.8%	97.7%
		(86.6%, 100.0%)	(86.7%, 100.0%)
Portugal	163	97.3%	96.4%
		(92.2%, 100.0%)	(91.3%, 100.0%)
Japan	8	99.1%	96.4%
		(90.0%, 100.0%)	(87.6%, 100.0%)
Sweden	549	100.0%	91.3%
		(83.1%, 100.0%)	(75.0%, 100.0%)
Chile	389	92.4%	90.8%
		(78.2%, 100.0%)	(74.0%, 100.0%)
Pakistan	26	91.5%	88.3%
		(86.7%, 96.6%)	(83.7%, 93.2%)
Saudi Arabia	70	81.0%	74.9%
		(74.5%, 87.3%)	(70.0%, 78.8%)
Mexico	298	78.3%	70.3%
		(70.7%, 85.9%)	(45.2%, 90.5%)
Iraq	92	76.5%	65.6%
		(61.7%, 87.7%)	(60.1%, 70.1%)
Israel	43	82.1%	65.4%
		(73.1%, 93.0%)	(46.6%, 78.9%)
Azerbaijan	34	77.3%	65.4%
		(31.4%, 94.1%)	(39.2%, 81.5%)
Armenia	206	72.3%	64.7%
		(42.3%, 88.5%)	(35.8%, 78.3%)
Peru	394	83.6%	64.0%
		(79.1%, 87.8%)	(47.6%, 77.4%)
Honduras	87	92.5%	63.3%
		(79.4%, 100.0%)	(28.7%, 84.9%)
Bolivia	175	93.1%	60.5%
		(83.4%, 99.7%)	(47.5%, 71.1%)
Brazil	366	92.3%	58.4%
		(87.1%, 98.6%)	(48.8%, 67.0%)
Russia	83	73.8%	57.7%
		(67.5%, 80.7%)	(46.9%, 66.8%)
Nigeria	4	72.5%	54.3%
		(14.5%, 94.5%)	(20.3%, 73.3%)
Panama	239	73.7%	51.5%
		(45.4%, 93.3%)	(9.1%, 83.3%)
India	19	68.3%	46.9%
		(61.0%, 74.5%)	(34.8%, 57.2%)
Afghanistan	30	63.7%	Not reliable
		(10.9%, 100.0%)	
Serbia	63	30.4%	Not reliable
		(4.8%, 85.2%)	
Iran	166	58.6%	20.4%
		(14.0%, 84.2%)	(7.0%, 83.7%)
Argentina	47	46.3%	Not reliable
		(12.8%, 100.0%)	
Guatemala	81	25.6%	Not reliable
		(8.9%, 89.5%)	

The ranking is in terms of outbreak progress in the medium scenario. Numbers in brackets are 80% prediction intervals. As positive scenarios predict a smaller final number of deaths, the outbreak progress is thus larger in the positive scenario. Note that, the estimated final death toll tends to underestimate the final results, thus the estimated outbreak progress serves both as a lower bound for future developments and as a guide of the dynamics of the evolution of the epidemic

We define the outbreak progress as the latest cumulative number of deaths per million divided by the predicted final total death toll. As the epidemic progresses, the outbreak progress increases and finally saturates to 1 when the epidemic ends. Note that, in a classical logistic curve, an outbreak progress of 50% indicates that the total number of deaths has reached its inflection point, which is also the time of the peak of the daily incidence curve. If the inflection point has been passed, the worst of the outbreak is over. The fitted trajectory, and thus the position of the inflection point and the predicted final death toll depends on country-specific factors identified in Sect. 3, most notably demographics and (early) intervention measures. Therefore, the outbreak progress can measure how mature the outbreak is in a country, and is more conservative than the same analysis based on confirmed cases, as the number of deaths is a lagging indicator behind confirmed cases.

In Table 2, we list the cumulative numbers of deaths per million population as of July 17, 2020, and the outbreak progress (death) in positive and medium scenarios. In Fig. 9, we plot the ensemble distribution of the final total number of deaths per million population for each country, which are based on the aggregation of the simulations in positive and medium scenarios. Those countries with a non-converged distributions are displayed in the left panel, while those with a converged distribution are in the right panel. The left side of each violin in cyan is based on data up to July 17, while the right side of each violin in grey is based on data up to April 24, when the first version of this paper was submitted. Note that, the logistic-type models are usually useful for understanding the short-term dynamics extending over a few days, but may become inadequate for long-term predictions due to the change of the fundamental dynamics resulting from government interventions, a second wave of outbreaks or other factors, as showed by large shifts of distributions in several countries.

**Fig. 10:**
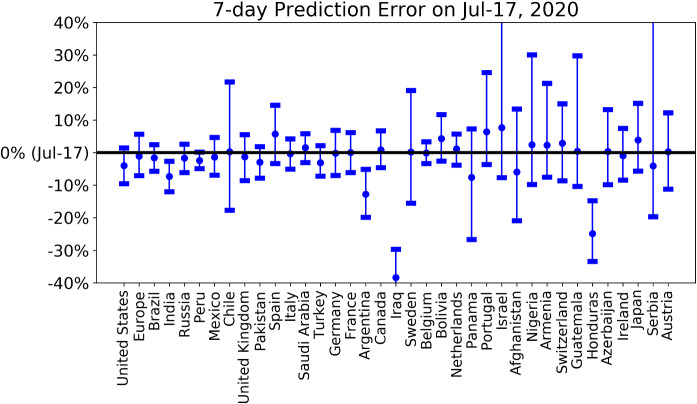
7-day prediction error of the forecast performed on July 10 for the total number of deaths for the various countries/regions. The horizontal line corresponds to empirical data on July 17. The errorbars are 80% prediction intervals and the middle dots are the median predictions based on the predictions from the positive and medium scenarios. A negative value corresponds to a prediction that underestimated the true realised value

To have a view of the performance of short-term predictions, we present the latest 7-day prediction errors for the total number of deaths in Fig. 10, based on positive and medium scenarios. One can see that our 7-day predictions based on data up to July 10th are correct in all matured countries and enjoy narrow prediction intervals. In contrast, our 7-day predictions underestimate the true numbers in immature countries, including India, Argentina, Iraq and Honduras. Until May 24, we uploaded a daily update of our projections and an analysis of forecasting errors online, and then shifted to a weekly update until July 3 [73]. We have now discontinued it as the epidemic enters second waves and other regimes with highly dependent country-specific characteristics.

As of July 17, 2020, the epidemic in Italy, Ireland, Germany, Austria, Turkey, Belgium, the United Kingdom, France, Netherlands, Spain, the USA, Canada, Switzerland, Portugal and Japan have almost ended, with the outbreak progress approaching 100%. Most of these countries have the earliest starts of the outbreaks, with Italy and Spain being the first two hotspots in Europe. However, the USA has started a second wave of outbreak with the daily confirmed cases keeping breaking records recently. This is likely due to the easing of the lockdown and street demonstrations where large numbers of people gather. This is not yet reflected significantly in the daily death data as there is usually a two to four weeks lag between the data of confirmed cases and deaths. Our model, which assumes a single “wave” by construction, is unable to characterise the second wave dynamics as shown in the supplementary figure. Resurgences of cases are also found in several matured countries including Germany, France, Austria, Portugal and others (mostly West European). Sweden, Chile and Pakistan are also in a matured stage with strong signs that inflection points have been passed and an outbreak progress of 85% to 95% in the medium scenario.

The next less matured group of countries are Saudi Arabia, Mexico, Iraq, Israel, Azerbaijan, Armenia, Peru, Honduras and Bolivia, which have their outbreak progress in the range of 60%-80%. They just have confirmed signals that their inflection points have been passed, and the shapes of their distribution are settling to the after-peak trajectory. Saudi Arabia and Israel are also in a second wave of outbreaks, which may change the previous inflection points and reduce the outbreak progress.

Brazil, Russia, Nigeria and Panama are in the third most matured group of countries, which are just at the inflection points and have higher uncertainties regarding their future scenarios. This is confirmed by the non-converged (Nigeria and Panama) or highly dispersed (Brazil and Russia) distribution of final deaths per million as shown in Fig. 9.

The remaining group includes India, Afghanistan, Serbia, Iran, Argentina and Guatemala, whose mortality curves have not significantly departed from the exponential or sub-exponential growth trajectory, indicating the early stage of the outbreak and high uncertainties for the future projections, as evidenced by their non-converged distributions of the final deaths in Fig. 9.

## Discussion

The SARS-CoV-2 / Covid-19 pandemic began in Wuhan, China, in January 2020. By 25 April 2020, when we submitted the present paper, it had spread to every country in the world and killed an estimated $\sim$ two hundred thousand people worldwide. As of 19 July 2020, 14.3 million cases of COVID-19 have been reported, including approximately 602,000 deaths. Mortality trends showed that it has been killing more people (normalised for population size) in Western countries than anywhere else outside of Wuhan. We set out to solve the riddle of why Western countries with their lavish healthcare systems were hardest hit. We also set out to understand why the impacts as measured by deaths per million population varied so much between the various Western countries.

Contrary to many early media reports, COVID-19 is quite specific about the individuals who are most susceptible and die. The largest group of casualties is found in the elderly, specifically the over 65-year-old cohorts. The mean age of the dead in the UK is about 80 similar to the mean age of the dead in Italy. Many of the dead are aged 80 or more and arguably were close to end of life, exhibiting comorbidities such as respiratory disorders, cancer and heart disease. In the UK, the 65+ group comprises 87% of all deaths. In the under 65 group that comprises 13% of all deaths, numerous reports suggest that it is the clinically obese who are most at risk. The deceased obese are also described as having comorbidities of diabetes, high blood pressure and atherosclerosis.

We have presented results on the mortality statistics of the COVID-19 epidemic in a number of countries. After drawing attention to many data quality issues, we have proposed a classification of countries in five groups, 1) Western countries, 2) East Block , 3) developed Southeast Asian countries, 4) Northern Hemisphere developing countries and 5) Southern Hemisphere countries. Comparing the number of deaths per million inhabitants, a pattern emerges in which the Western countries exhibit the largest mortality rate. Furthermore, comparing the running cumulative death tolls at the same level of outbreak progress in different countries reveals several subgroups within the Western countries and further emphasises the difference between the five groups. Rationalising the drastic differences in performance goes beyond the present article, but visualising these differences calls for in-depth investigations of causative or correlated factors such as preparedness, development and organisation of the health-care system (public-private, governance structure, etc), culture (close physical contacts versus social distance, hygiene, etc), stringency of the reactions to control the epidemic, temperature, geography, population density, general health and age distribution and so on.

Inspired by a number of reports, we presented a synthetic plot of the relationship between deaths per million and life expectancy in different countries, taken as a proxy of the preponderance of elderly people in the population. Our analysis strongly suggests that a main reason behind the relatively more severe COVID-19 epidemic in the Western countries is their larger population of elderly people, with the exceptions such as Norway and Japan, for which other factors seem to dominate. Following the outcomes of the epidemic in these countries and extending the comparative analysis that we present will provide important insights to learn and implement as much as possible the procedures that have been successful.

Within the Western countries, we report a large range of outcomes, despite similar demographics. Our comparison between countries at the same level of outbreak progress also allowed us to identify and quantify a measure of efficiency of the level of stringency of confinement measures. This delicate and controversial subject finds here an objective analysis, which confirms that stronger stringency on confinement measures during the early stages of the epidemic are significantly negatively correlated with deaths per million. We found a correlation between mortality and a stringency metric that quantifies 7 different measures such as closure of schools, bans on large public meetings and locking down populations.

Looking at Fig. 8 (and extending the time window) shows that increasing the stringency from 20 to 60 during the onset of the epidemic decreases the death count by about 50 lives per million within two weeks, or roughly 350 lives per million until July, i.e. about 20’000-25’000 lives for the UK. Thus, unsurprisingly, preventing people from meeting and moving around provides a barrier against the propagation of the virus. But the effect up to date is arguably small, and largely depends on when the confinement measures were put in place. As argued by the epidemiologist behind Sweden’s trust-based approach to tackling the epidemic, closedown, lockdown, closing borders, none of these measures may have historical scientific basis when the epidemic is already well advanced^6^. Moreover, the lockdown strategy faces the paradoxical desires of, on the one hand, having the least number of infected people. On the other hand, the governments of locked down countries worry that only a tiny percentage of the population has been exposed to the virus (at the time of writing, estimations vary from a few percent to 10 percent), so that any deconfinement may lead immediately to a second epidemic wave, barring achieving a fraction of about 60% of the population^7^ being protected by previous infections or vaccination. Recent studies suggest that the herd immunity threshold could be lower, perhaps even as low as 20% as a result of partial preexisting immunity and strong heterogeneity of $R_0$ [74, 75].

We have also performed logistic equation analyses of deaths as a means of tracking the maturity of outbreaks and estimating ultimate mortality. We use four different models to identify model error and robustness of results. This quantitative analysis allowed us to assess the outbreak progress in different countries, differentiating between those that are at a quite advanced stage and close to the end of the epidemic from those that are still in the middle of it.

Western governments will be judged on two metrics. First, they will be judged on the ultimate number of deaths per million people. Second, they will be judged on the economic and social costs of their actions. With only one exception (Sweden), Western governments have taken extreme actions to combat COVID-19, with different levels of stringency across countries (with the case of Asian countries needing a different discussion). These actions include confining whole populations at home, shutting down large sectors of their economies, throwing tens of millions out of work and running up massive debts. The common estimation of the economic cost of these measures is widely estimated around 10% of GDP, and is likely to grow as time passes. Strict confinement can also have serious consequences in terms of mental illness and neglect of other conditions. The breakdown of supply chains threatens famines of ‘biblical proportions’, according to a recent UN report.

Given these conclusions, and with the perspective and experience of the epidemics of the past, we have to ask whether the extraordinary response levels, with no equivalent in history, are justified by the threats posed by the SARS-CoV-2 virus. Was it worth putting the prosperity of whole nations at risk in this way? In a column entitled “Coronavirus, watch out for danger, but not the one you think”, Professor Gilbert Deray, from the Pitié-Salpêtrière Hospital in Paris summarises the problem as follows: For 30  years, from my hospital observatory, I have lived through many health crises, HIV, SARS, MERS, resurgence of tuberculosis, multi-resistant bacteria, we have managed them calmly and very effectively. None of them have given rise to the current panic. I have never experienced such a level of concern for an infectious disease.

This is the first time that people’s health has been put ahead of economic interests at such a global level. But was this well thought out? A society cannot save every life, it has to make reasonable choices. A wise policy requires intelligent calculations that arbitrate and balance medical, social, economic, equity and intergenerational considerations. Have we been collectively blinded by short-sighted medical considerations and been overwhelmed by a pandemic of fear [76]?

What could have been done better? Without doubt, many studies will be published on this question. It seems to us that the well-conceived Pandemic Influenza Plan elaborated by nations in collaboration with the WHO have been superseded by a pandemic of fear. For example, translating from the latest Swiss Pandemic Influenza Plan (2018), we read that the pandemic management strategies are designed to reduce at the very least deleterious consequences of the pandemic and the priority objectives are: (i) to protect and preserve the life, well-being and health of the population; (ii) keep casualties to a minimum and (iii) prevent the occurrence of subsequent economic damage. Further, the Swiss-WHO based plan continues with the following statement (www.bag.admin.ch/bag/fr/home/das-bag/publikationen/broschueren/publikationen-uebertragbare-krankheiten/pandemieplan-2018.html): “Preventing annfluenza pandemic by means of containment measures seems, according to current knowledge, unrealistic both nationally and internationally. Application of selective measures as part of containment interventions can be used to prevent the spread of disease, limit local outbreaks during the initial phase and thus reduce transmission, and thus providing targeted protection for vulnerable people. These measures will not prevent the spread of the pandemic, but they will eventually help to slow it down and thus gain time. Containment measures therefore have local operational objectives and contribute to the mitigation strategy”. These clear instructions have the further benefit of removing uncertainty, which has been a major cause of stress in affected population [77]. The science of epidemics is well-established and dictates that at the very beginning of an epidemic, stringent measures must been put in place to isolate the local clusters and to protect the vulnerable. It thus seems to us that the undifferentiated and unprecedented global and complete lockdown in many countries has not been rooted in sound scientific thinking based on the calm assessment based on all previous knowledge, but has fallen trap to quickly cooked models that catalysed an atmosphere of fear amplified by the social media and media machine as sellers of attention [78].

### Electronic supplementary material

Below is the link to the electronic supplementary material.

Supplementary material 1 (pdf 169 KB)

Supplementary material 2 (pdf 7179 KB)

## Figures and Tables

**Fig. 11:**
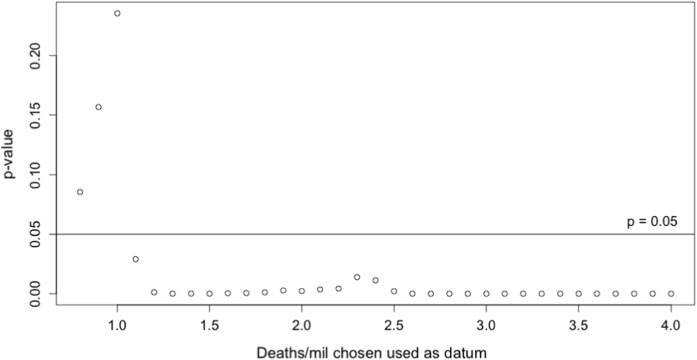
Significance of non-zero linear relationship of average weekly growth rates (6) before and after the reference times $T_x$ when *x* deaths/mil was reached; using all countries in Table 1 with $C_{T_{0.8}-7}>0$ ($n=20$)

**Fig. 12:**
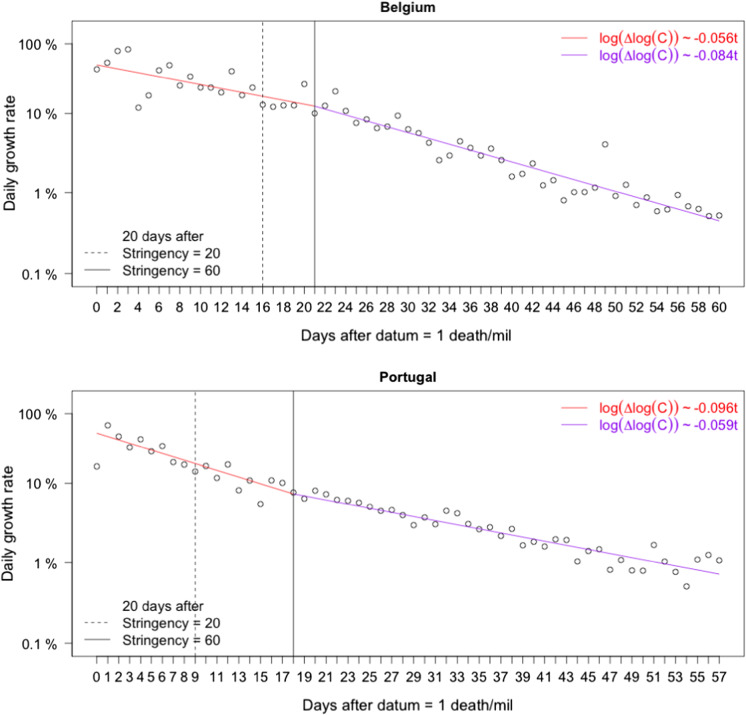
Daily growth rate of mortality in Belgium and Portugal (log scale) and linear fits according to 7. The solid and dashed lines indicate the date where increasing levels of stringency were reached, shifted by 20  days (average time lag of infection to death)

**Fig. 13:**
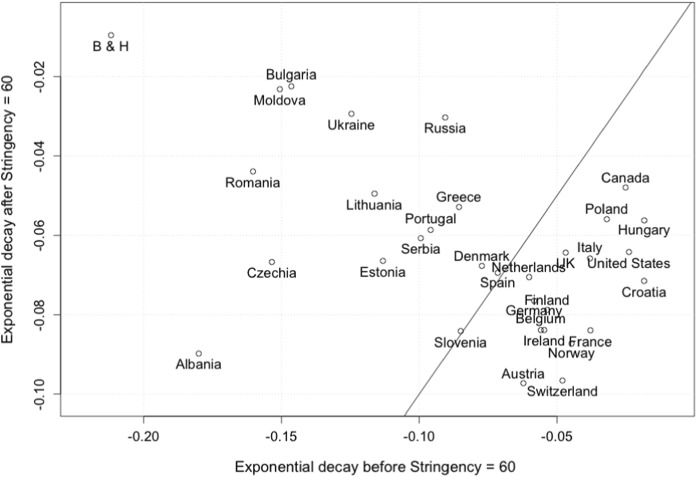
Exponential decay parameters $\gamma$ in (7) before and after $t^*$ defined in (8). The solid line indicates a constant rate of decay

**Fig. 14:**
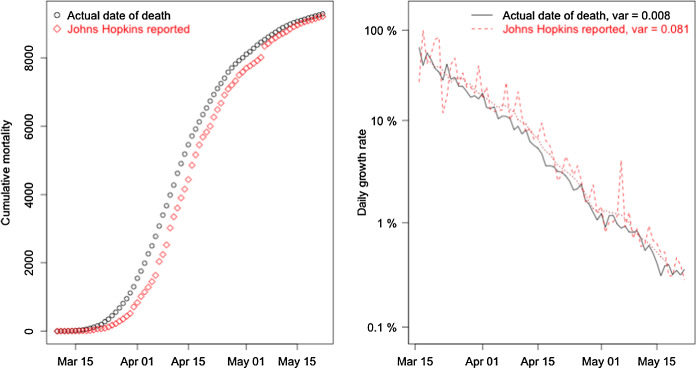
Reported versus actual mortality in Belgium. Cumulative mortality *C*(*t*) shows a reporting delay of around 3  days (left) and significant reduction in noise of the daily log growth rate $\log \left( \log \left( {C(t+1)}/{C(t)}\right) \right)$ (right)

## A Appendix

### A.1 Choice of reference time

As discussed in Sect. 3.2.2, to allow for a suitable comparison of mortality curves for countries of different population, we align countries at a suitable reference time of reaching a certain number of deaths/mil. Here, we need to take the following into account.

1. Due to the considerable variance reported in time of infection to death and potential early imported cases, we expect a larger value of deaths/mil to be more robust towards noise in the early reported data of COVID-19 deaths.
2. If the reference time is too large, it may be influenced considerably by country-specific growth rates, which can lead to spurious results in Sect. 3.3.2 on lockdown policies.

To address this, we look at growth rates before and after a range of reference times $T^*\in \{T_{0.8},T_{0.9},T_{1},T_{1.1},\cdots ,T_{4}\}$, where $T_x$, for each country, denotes the time when *x* deaths/mil was reached. We then calculate average weekly growth rates for cumulative mortality *C*(*t*),

6 $$\begin{aligned} \frac{1}{7}\log \left( \frac{C({T^*})}{C({T^*-7})}\right) \quad \text {and}\quad \frac{1}{7}\log \left( \frac{C({T^*+7})}{C({T^*})}\right) , \end{aligned}$$

and test for a significant linear relationship across countries, see Fig. 11. We exclude reference times with a significant correlation and choose 1 death/mil for our analysis.^8^

### A.2 Impact of lockdown policies in individual countries.

To complement our analysis in Sect. 3.3.2 above, we study the potential impact of intervention and lockdown policies on mortality growth rates. To this end, we denote by *C*(*t*) the cumulative number of COVID-19 deaths in a fixed country. As discussed in Sect. 4, the growth rate of *C* can be adequately described by logistic-type models, which capture the (combined) influence of lockdown policies, increased awareness and personal hygiene, a reducing number of susceptible individuals and other potential factors. While these models are suitable to capture a full epidemic outbreak, it has been noted in [72] for the number of confirmed cases that the reduction in the daily growth rate can be approximated by a simpler exponential decay on shorter time intervals.^9^. More specifically, on an interval $[\underline{t},\overline{t}]$, we fit a segmented linear regression

7 $$\begin{aligned} \begin{aligned} \log \left( \log \left( \dfrac{C(t+1)}{C(t)}\right) \right)&=a-\gamma _1(t-\underline{t}),&t\in [\underline{t},t^*]\\ \log \left( \log \left( \dfrac{C(t+1)}{C(t)}\right) \right)&=a-\gamma _1(t^*-\underline{t})-\gamma _2(t-t^*),\quad&t\in [t^*,\overline{t}] \end{aligned} \end{aligned}$$

with exponential decay parameters $\gamma _{1}$ and $\gamma _2$. To quantify the potential impact of lockdown policies, we compare the decay parameter before and after the time we expect to see a reduction in mortality growth rates. In particular, using the Stringency Index of [46] as above, we set

8 $$\begin{aligned} t^*= & {} \text {[time Stringency Index of 60 was first reached]}\nonumber \\&+\text {20\,days}, \end{aligned}$$

and compare the exponential decay parameters before and after $t^*$.

As an example, Fig. 12 shows exponential decay rates for Belgium and Portugal. For Belgium, we find a significant increase in $\gamma$, indicating a decrease in infectivity around the time lockdown policies were implemented. For Portugal, in comparison^10^, we first note a substantially larger initial rate of decay, which could be due to increased awareness and voluntary social distancing, efficiency of early intervention measures or other country-specific factors. Moreover, we see a decrease of the decay parameter at $t^*$, which one would expect to see from the evolution of an epidemic with constant infectivity (as predicted by a standard SIR model), and we cannot deduce that additional lockdown measures were particularly effective. Figure 13 shows the result of this analysis for the countries analysed in Sect. 3.3.2. We note that a majority of countries show an increasing or approximately constant rate of exponential decay $\gamma$ around the time of lockdown (lower right corner of the figure), indicating a decrease in infectivity. Plots for individual countries can be found in the supplements. This analysis would benefit from data collected with actual date of death, see section A.3.

### A.3 Reporting delay and additional noise in daily reported data.

The extensive data base of Johns Hopkins University collects mortality data as reported by governments and health agencies worldwide. In most cases, death from COVID-19 gets reported with several days delay, and this reporting introduces additional noise to the data. In Fig. 14, we show cumulative mortality and its growth rate in the first two months of the epidemic in Belgium, using both reported data from JHU [37] as well as daily update data sorted by actual date of death from press conferences and reports of Belgian officials [80]. We note a significant delay of roughly 3 days in the reporting of deaths, as well as a significant reduction of noise in the data, even after accounting for weekly seasonality in both time series. Availability of this data for a wide range of countries would significantly improve modelling and statistical analysis, for example, detecting change points in the log growth rate of Fig. 12 to confirm the choice of partition at $t^*$ in (8).

## Footnotes

1 Because the dynamic range between countries is so large, corrections for reporting standard tend not to have a material impact on interpretations.

2 The potential beneficial influence of warmer temperatures has been noted in [38], but has been questioned to suffice in reducing the transmission rate in the case of a novel virus [39].

3 In particular, we corrected countries with indication of underreporting: Spain (factor 0.78), Portugal (0.8), UK (0.56), Belgium (0.95), Italy (0.63), Netherlands (0.54) and Austria (0.53).

4 Below we consider averages on intervals, and the exact distribution is not essential here.

5 In Belgium, 46% of the general practitioners-patient encounters are home visits. This is the highest percentage in the world [51]. Most of these general practitioners are mobile entrepreneurial nurses who do home visits via several rounds a day. Thus, they have high incentives to do what their clients/patients at home demand, whether or not it is reasonable from a professional point of view [52].

6 https://www.nature.com/articles/d41586-020-01098-x.

7 This fraction of 60% is derived from an assumed average infection factor $R_0 =2.5$, so that the effective infection factor $R_\mathrm{eff}$ when a fraction *p* of the population is immune becomes $R_\mathrm{eff} = R_0 (1-p)$. Then, solving for *p* such that $R_\mathrm{eff} \le 1$ gives $p \ge 60\%$.
