# Supplementary material for: Interpreting, analysing and modelling COVID-19 mortality data
Source: Nonlinear Dyn. 2020 Oct 1;101(3):1751–76. doi: 10.1007/s11071-020-05966-z (PMC7527427; doi:10.1007/s11071-020-05966-z)
Supplement: Supplementary file 1 — Supplementary material 1 (pdf 169 KB) [file 11071_2020_5966_MOESM1_ESM.pdf]

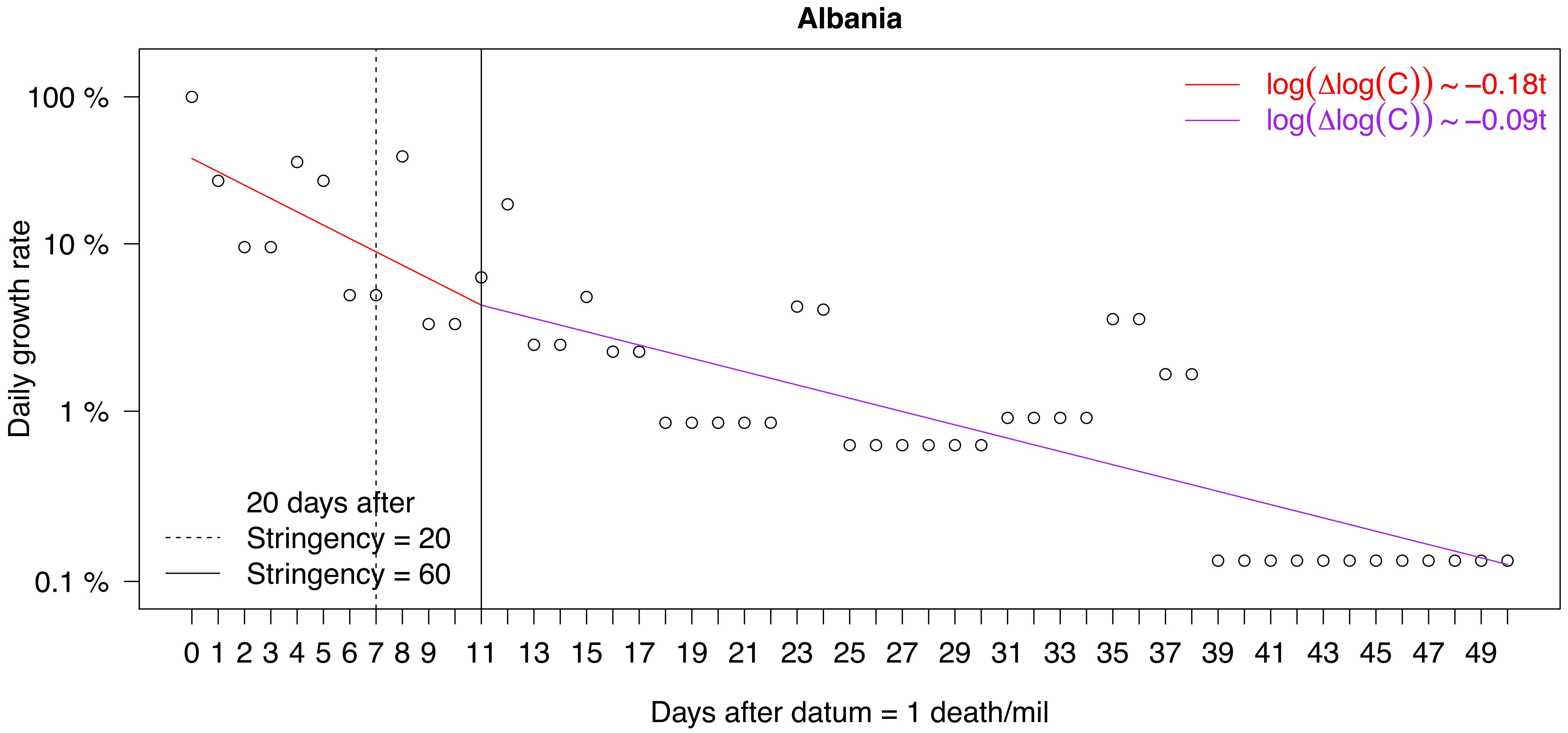

Albania
log(Δlog(C)) ~ −0.18t
log(Δlog(C)) ~ −0.09t
Daily growth rate
100 %
10 %
1 %
0.1 %
20 days after
Stringency = 20
Stringency = 60
0 1 2 3 4 5 6 7 8 9 11 13 15 17 19 21 23 25 27 29 31 33 35 37 39 41 43 45 47 49
Days after datum = 1 death/mil

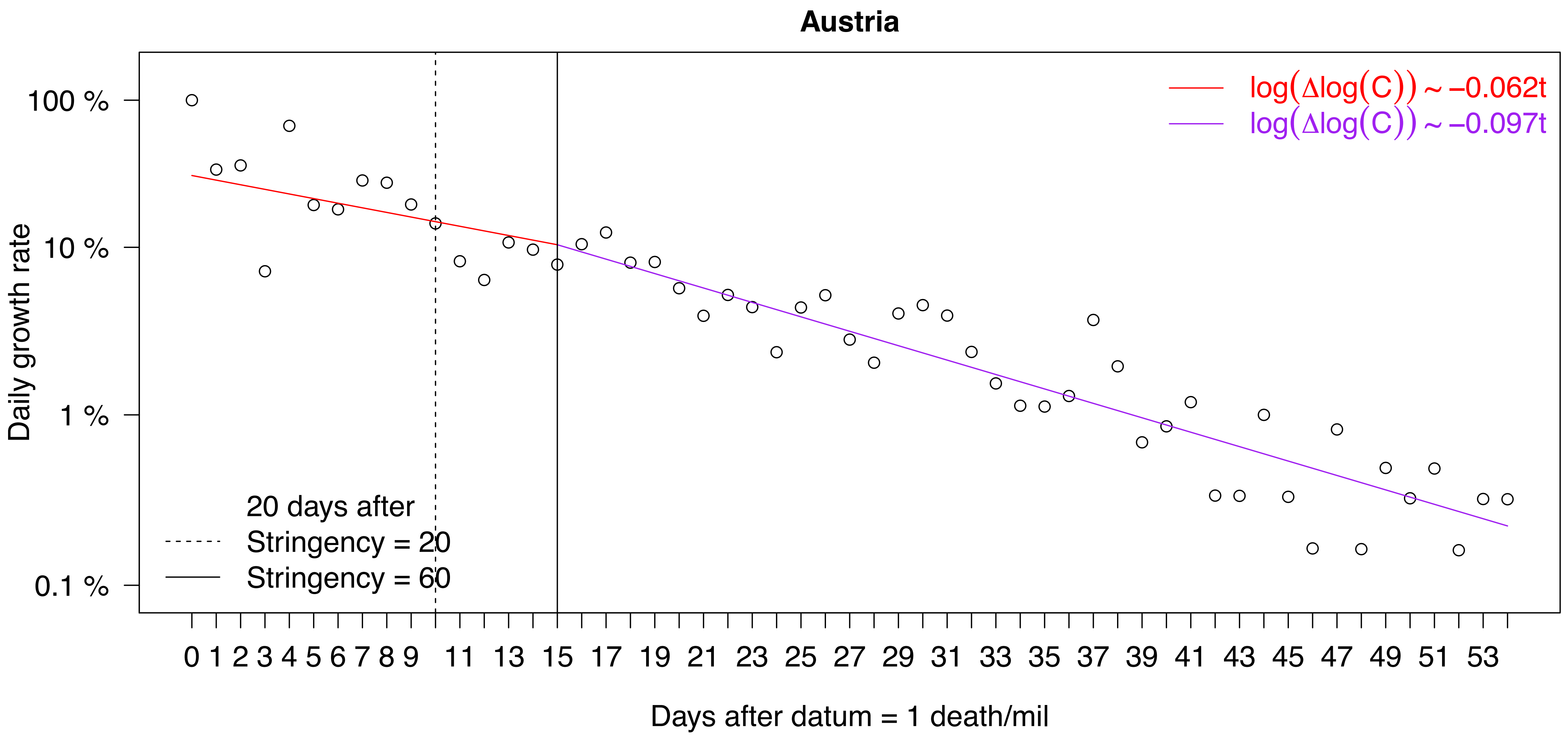

Austria
log(Δlog(C)) ~ −0.062t
log(Δlog(C)) ~ −0.097t
100 %
10 %
1 %
0.1 %
Daily growth rate
20 days after
Stringency = 20
Stringency = 60
0 1 2 3 4 5 6 7 8 9 11 13 15 17 19 21 23 25 27 29 31 33 35 37 39 41 43 45 47 49 51 53
Days after datum = 1 death/mil

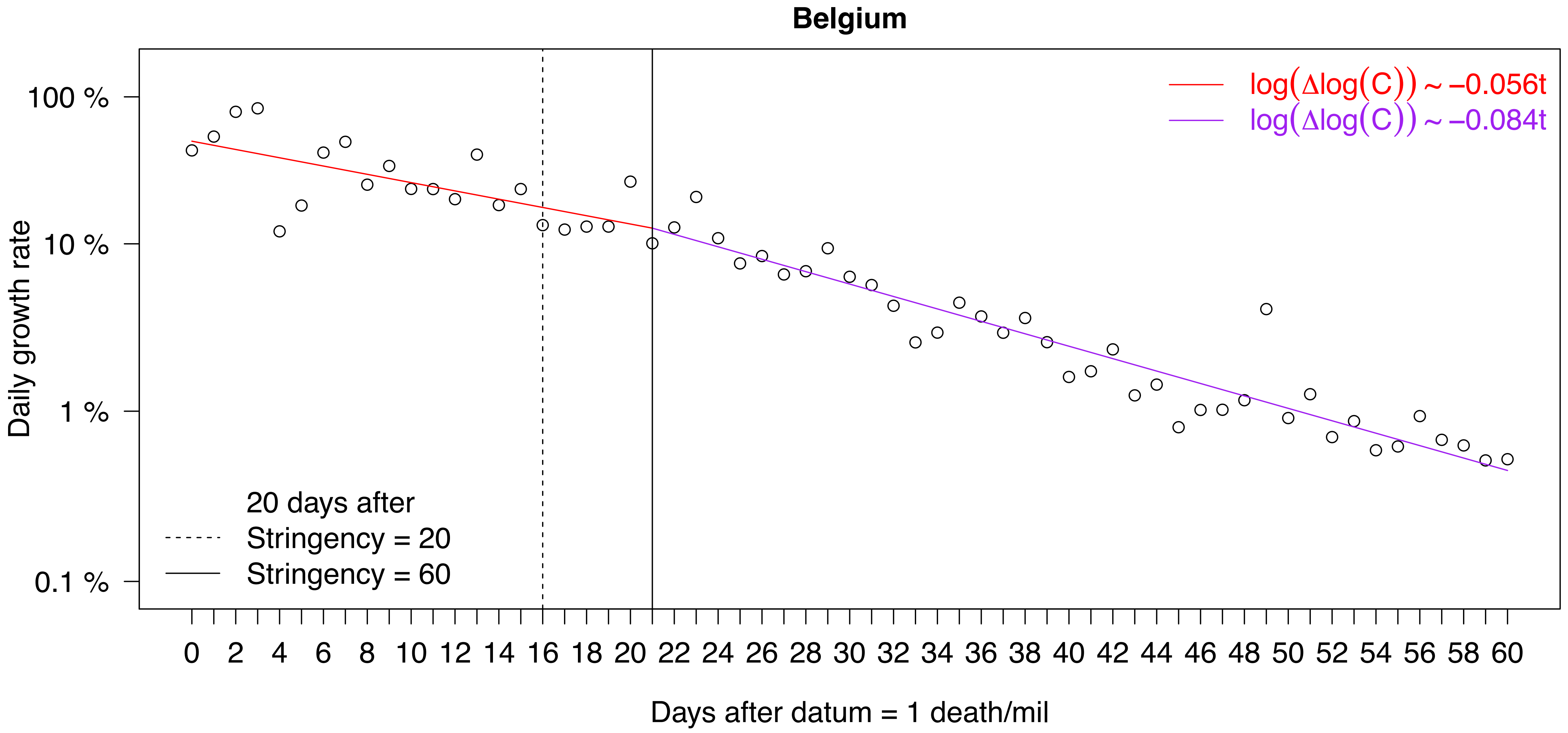

Belgium
log(Δlog(C)) ~ −0.056t
log(Δlog(C)) ~ −0.084t
100 %
10 %
1 %
0.1 %
Daily growth rate
20 days after
Stringency = 20
Stringency = 60
0 2 4 6 8 10 12 14 16 18 20 22 24 26 28 30 32 34 36 38 40 42 44 46 48 50 52 54 56 58 60
Days after datum = 1 death/mil

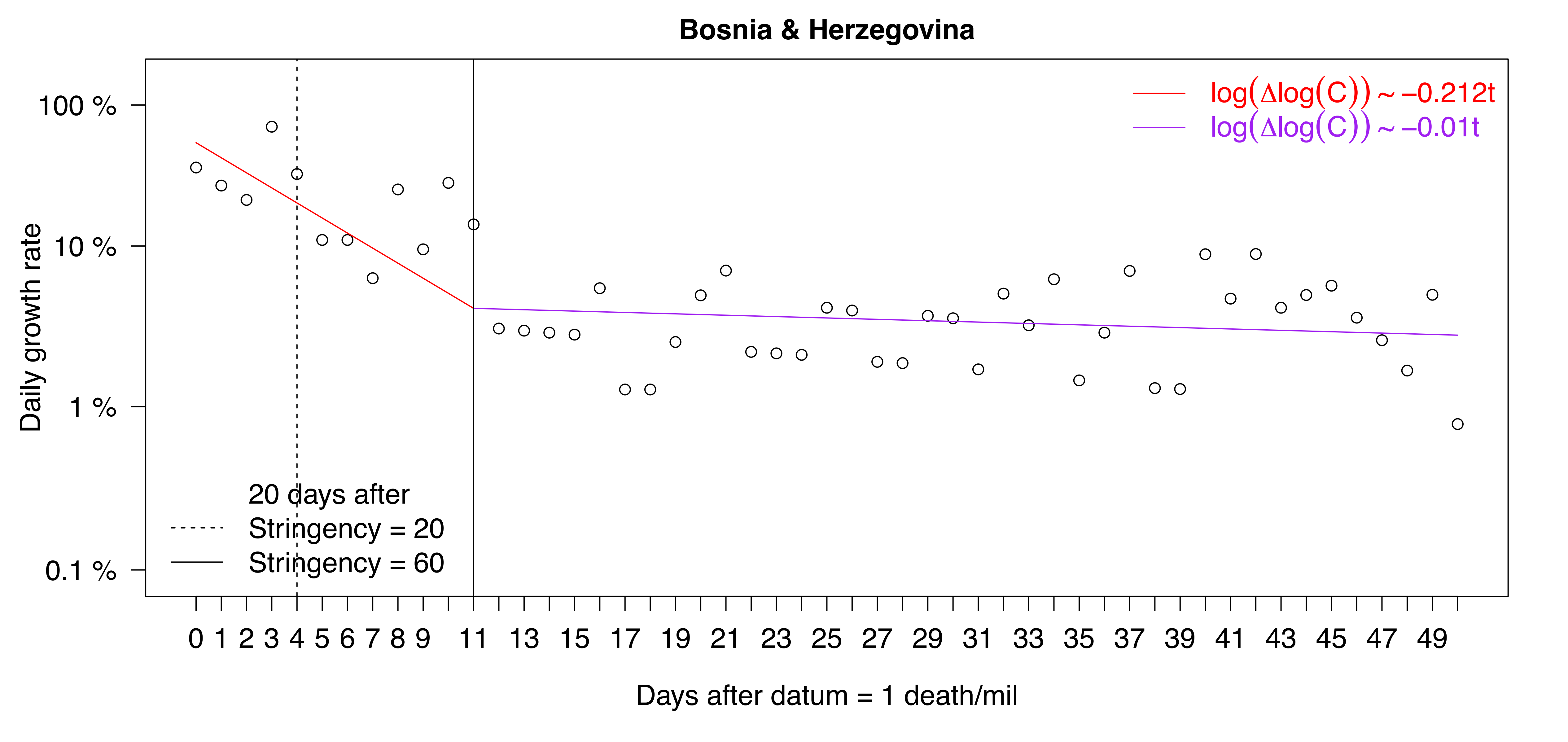

Bosnia & Herzegovina
log(Δlog(C)) ~ −0.212t
log(Δlog(C)) ~ −0.01t
100 %
10 %
1 %
0.1 %
Daily growth rate
20 days after
Stringency = 20
Stringency = 60
0 1 2 3 4 5 6 7 8 9 11 13 15 17 19 21 23 25 27 29 31 33 35 37 39 41 43 45 47 49
Days after datum = 1 death/mil

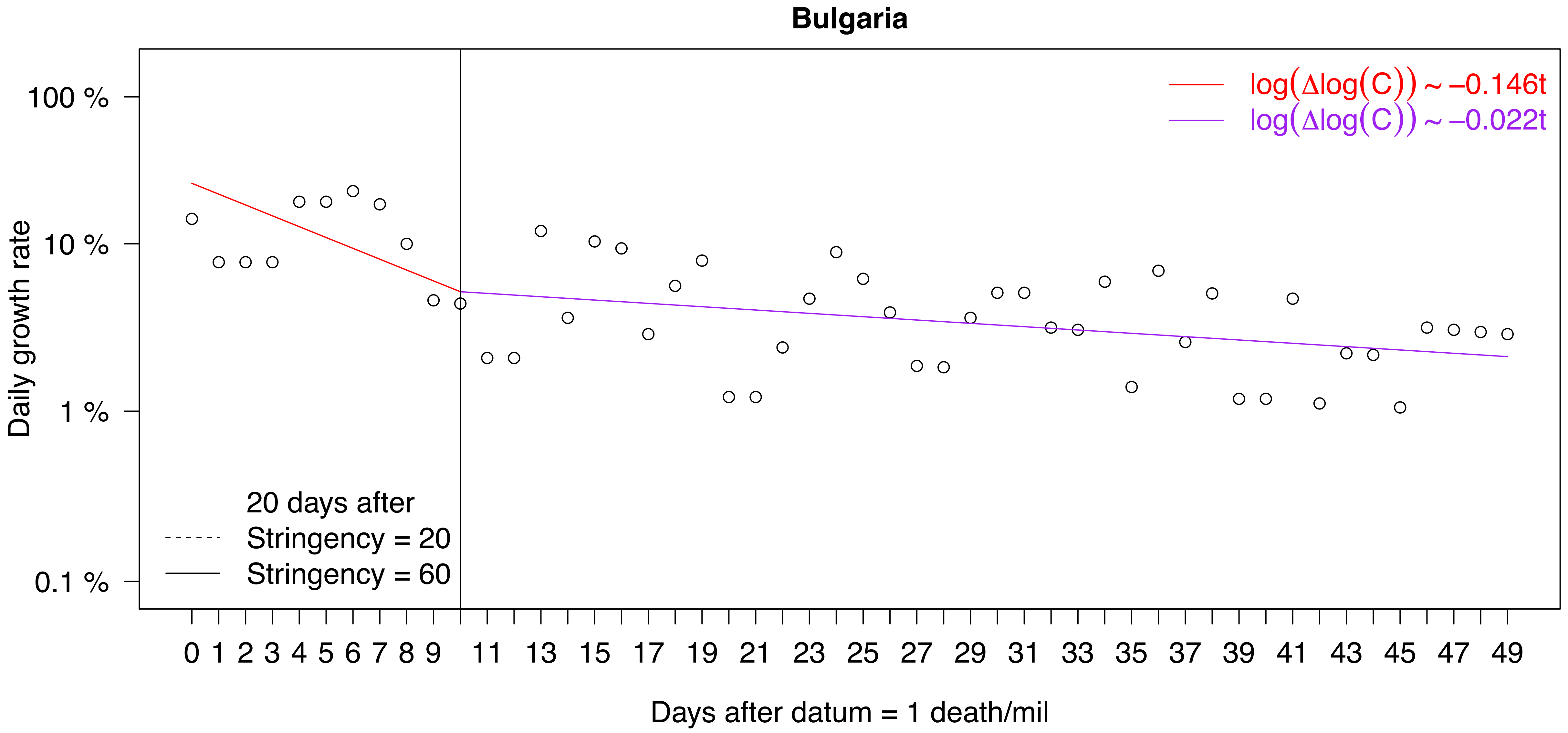

Bulgaria
log(Δlog(C)) ~ −0.146t
log(Δlog(C)) ~ −0.022t
Daily growth rate
100 %
10 %
1 %
0.1 %
20 days after
Stringency = 20
Stringency = 60
0 1 2 3 4 5 6 7 8 9 11 13 15 17 19 21 23 25 27 29 31 33 35 37 39 41 43 45 47 49
Days after datum = 1 death/mil

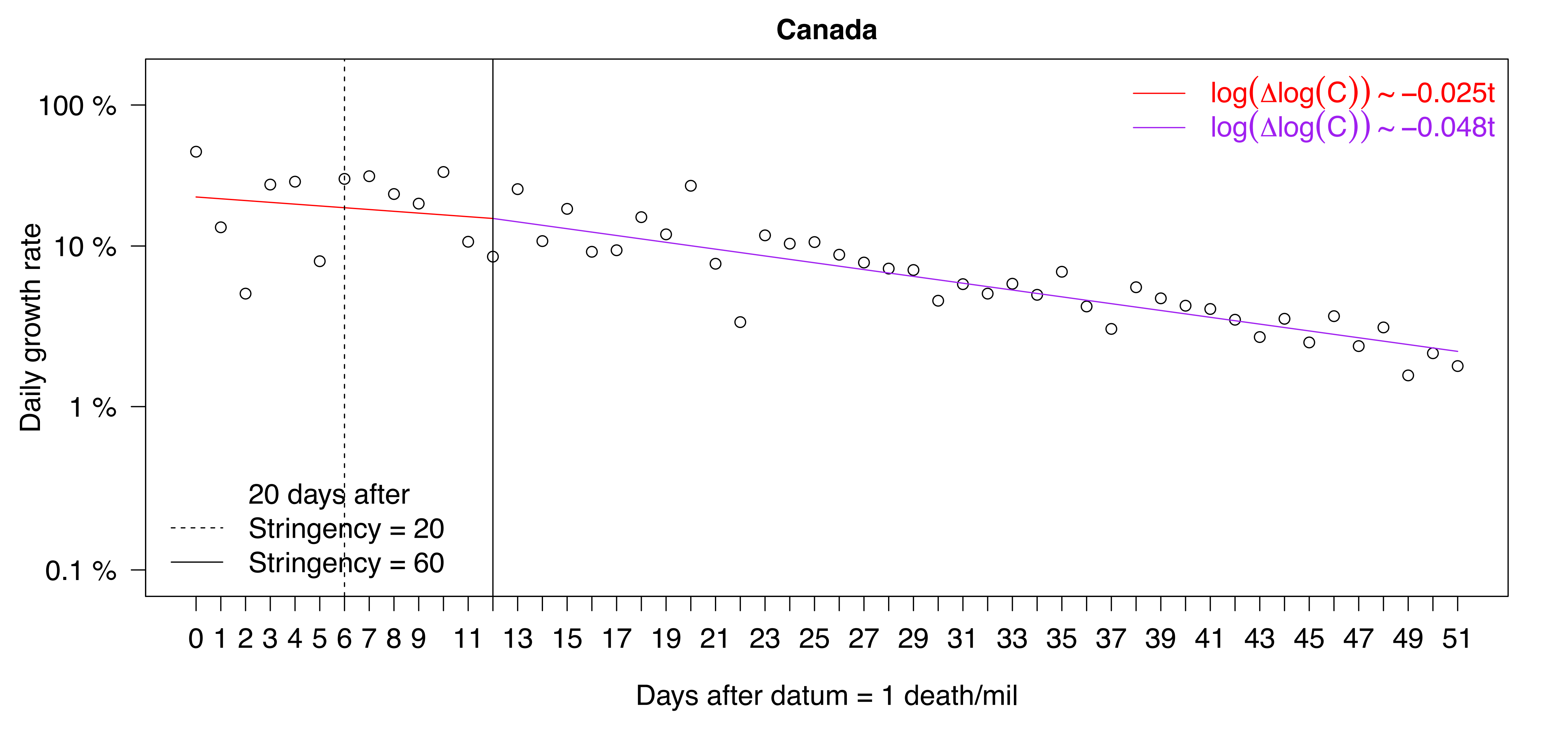

Canada
log(Δlog(C)) ~ −0.025t
log(Δlog(C)) ~ −0.048t
100 %
10 %
1 %
0.1 %
Daily growth rate
20 days after
Stringency = 20
Stringency = 60
0 1 2 3 4 5 6 7 8 9 11 13 15 17 19 21 23 25 27 29 31 33 35 37 39 41 43 45 47 49 51
Days after datum = 1 death/mil

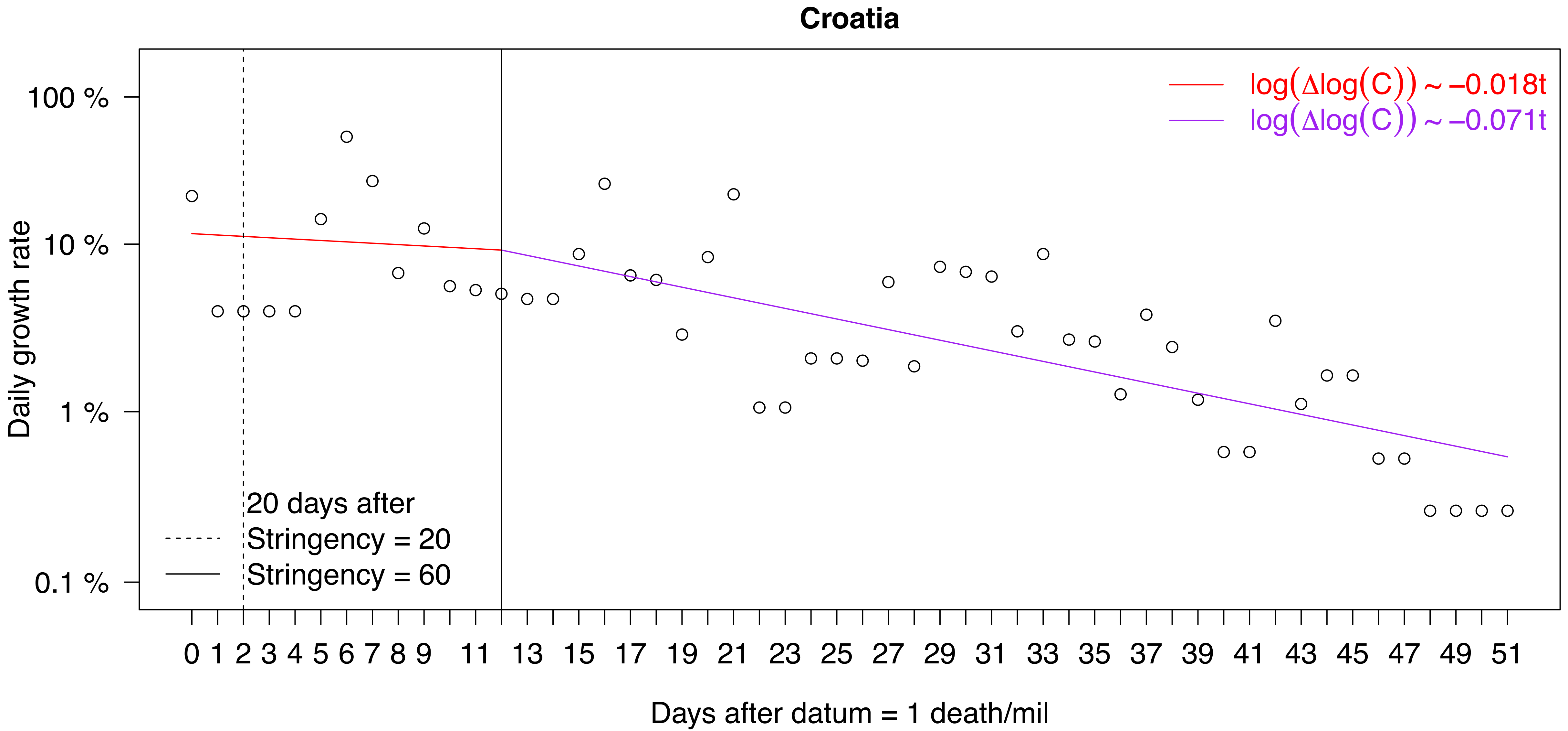

Croatia
log(Δlog(C)) ~ −0.018t
log(Δlog(C)) ~ −0.071t
100 %
10 %
1 %
0.1 %
Daily growth rate
20 days after
Stringency = 20
Stringency = 60
0 1 2 3 4 5 6 7 8 9 11 13 15 17 19 21 23 25 27 29 31 33 35 37 39 41 43 45 47 49 51
Days after datum = 1 death/mil

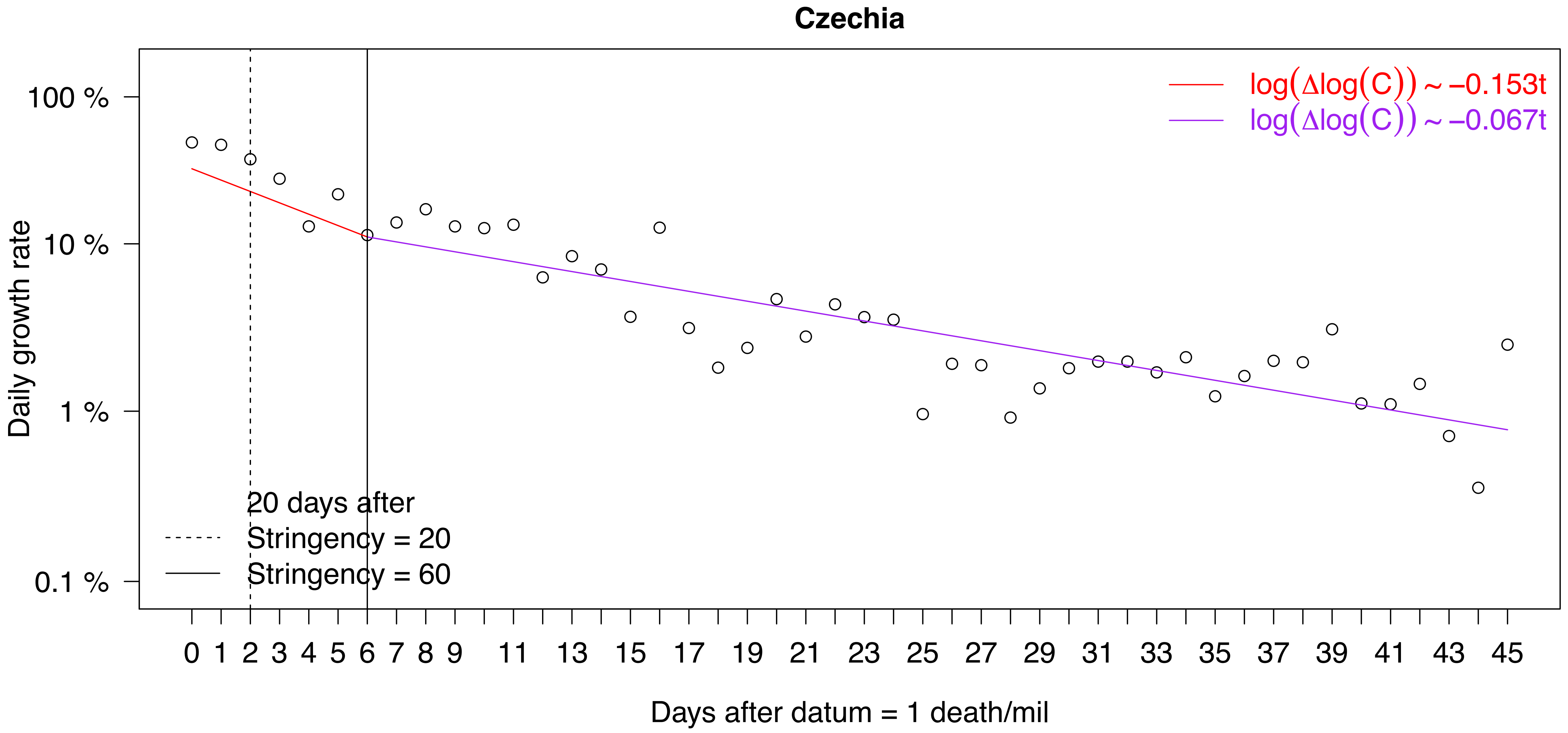
Czechia
log(Δlog(C)) ~ −0.153t
log(Δlog(C)) ~ −0.067t
100 %
10 %
1 %
0.1 %
Daily growth rate
20 days after
Stringency = 20
Stringency = 60
0 1 2 3 4 5 6 7 8 9 11 13 15 17 19 21 23 25 27 29 31 33 35 37 39 41 43 45
Days after datum = 1 death/mil

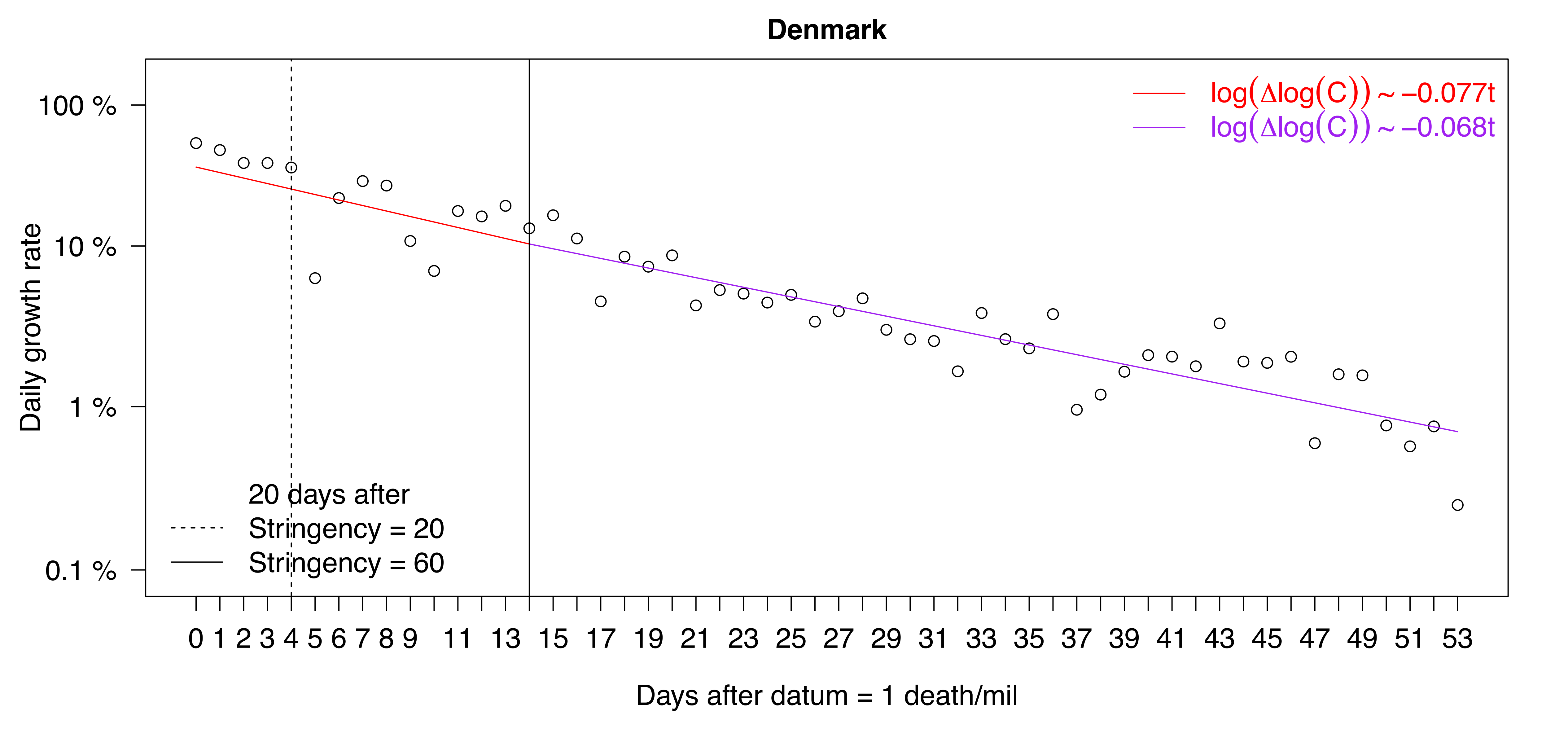

Denmark
$\log(\Delta\log(C)) \sim -0.077t$
$\log(\Delta\log(C)) \sim -0.068t$
100 %
10 %
1 %
0.1 %
Daily growth rate
20 days after
Stringency = 20
Stringency = 60
0 1 2 3 4 5 6 7 8 9 11 13 15 17 19 21 23 25 27 29 31 33 35 37 39 41 43 45 47 49 51 53
Days after datum = 1 death/mil

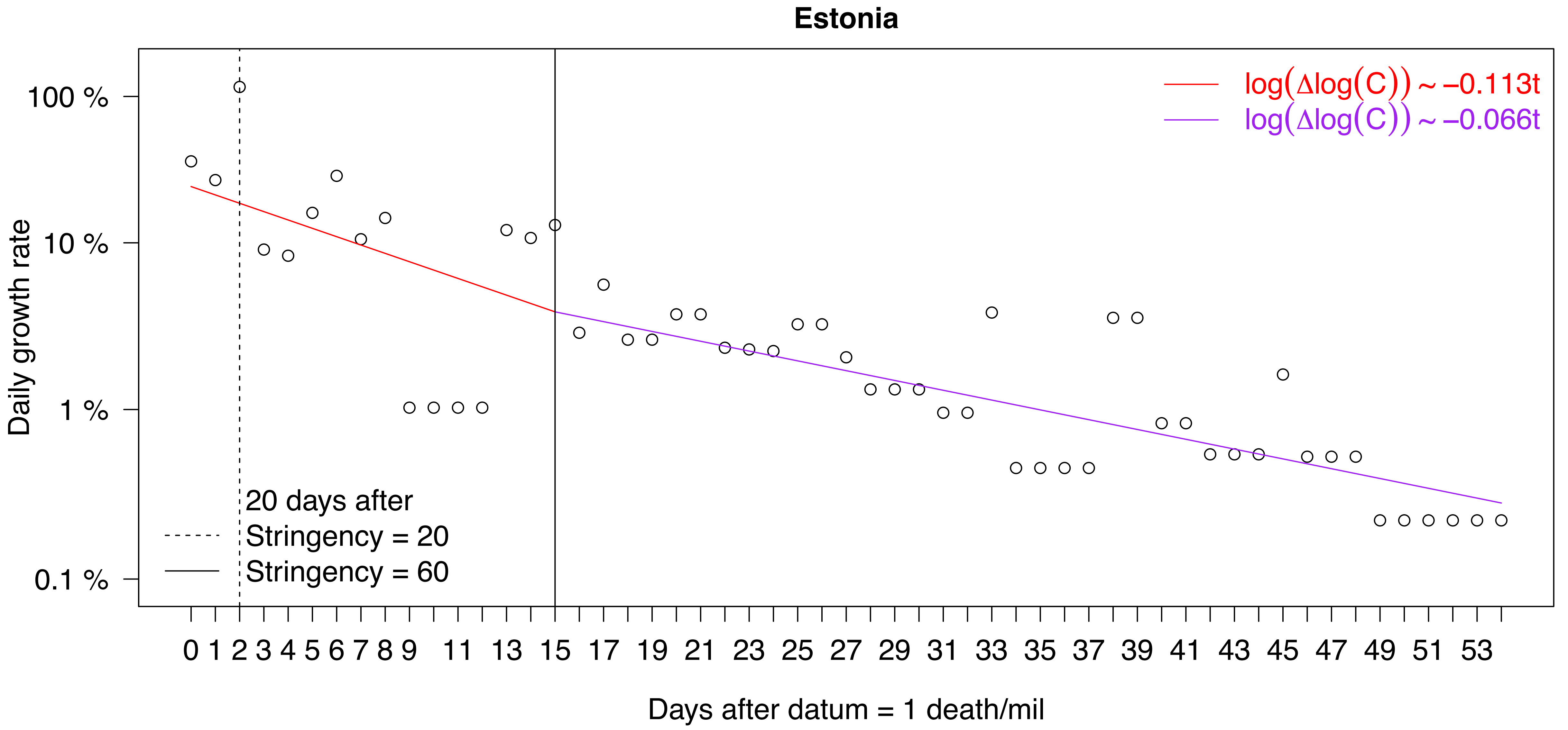

Estonia
log(Δlog(C)) ~ −0.113t
log(Δlog(C)) ~ −0.066t
100 %
10 %
1 %
0.1 %
Daily growth rate
20 days after
Stringency = 20
Stringency = 60
0 1 2 3 4 5 6 7 8 9 11 13 15 17 19 21 23 25 27 29 31 33 35 37 39 41 43 45 47 49 51 53
Days after datum = 1 death/mil

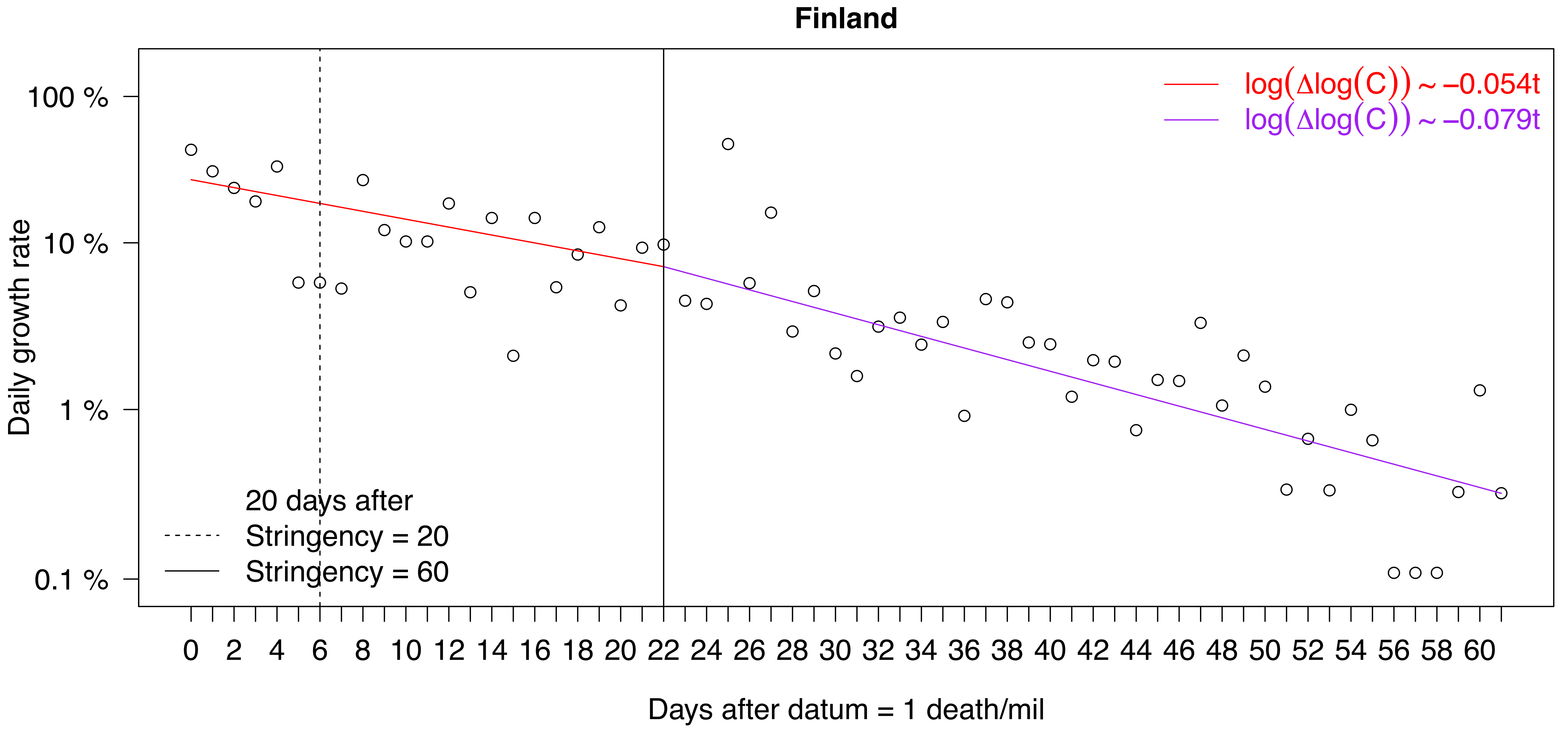

Finland
100 %
10 %
1 %
0.1 %
Daily growth rate
log(Δlog(C)) ~ −0.054t
log(Δlog(C)) ~ −0.079t
20 days after
Stringency = 20
Stringency = 60
0
2
4
6
8
10
12
14
16
18
20
22
24
26
28
30
32
34
36
38
40
42
44
46
48
50
52
54
56
58
60
Days after datum = 1 death/mil

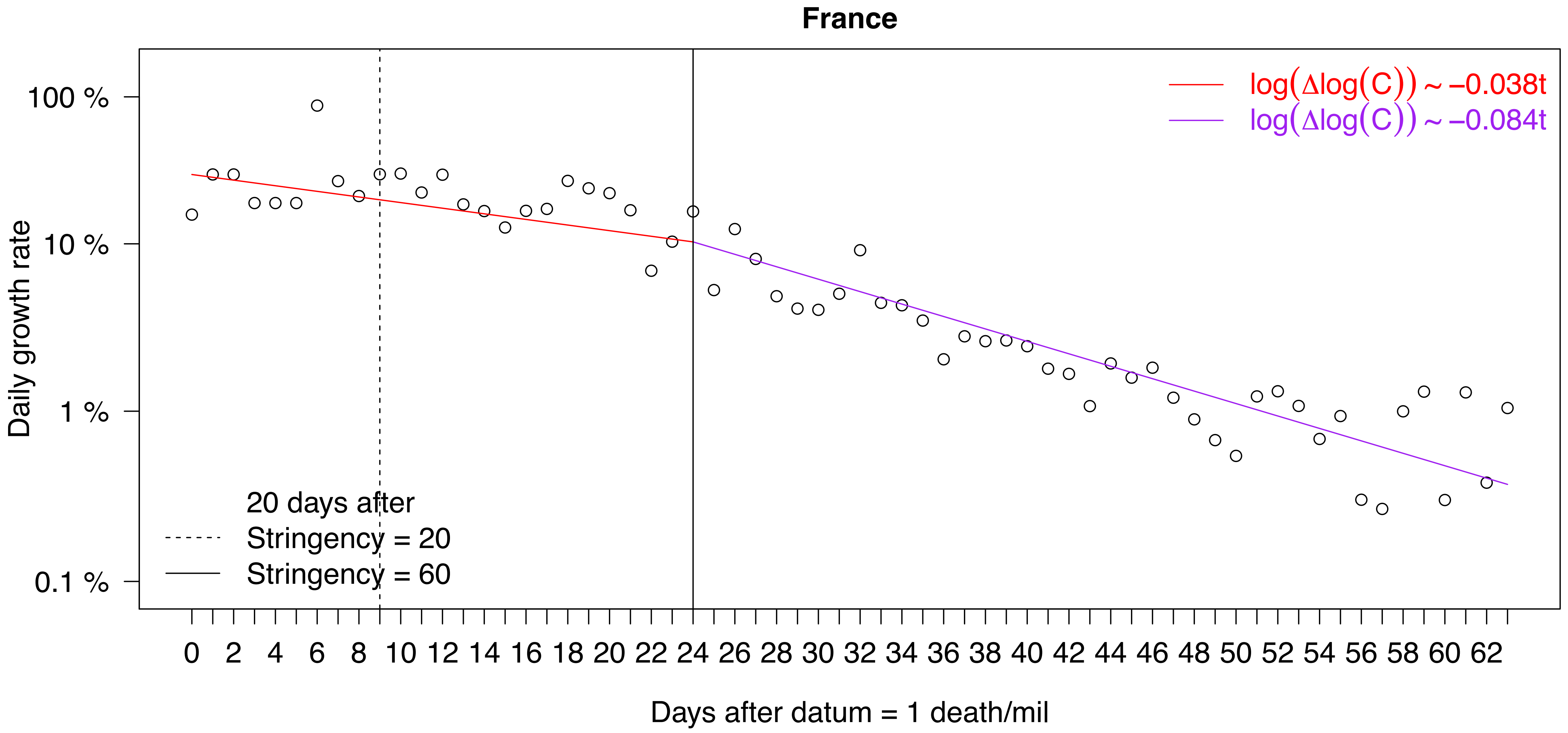
France
log(Δlog(C)) ~ −0.038t
log(Δlog(C)) ~ −0.084t
Daily growth rate
100 %
10 %
1 %
0.1 %
20 days after
Stringency = 20
Stringency = 60
0 2 4 6 8 10 12 14 16 18 20 22 24 26 28 30 32 34 36 38 40 42 44 46 48 50 52 54 56 58 60 62
Days after datum = 1 death/mil

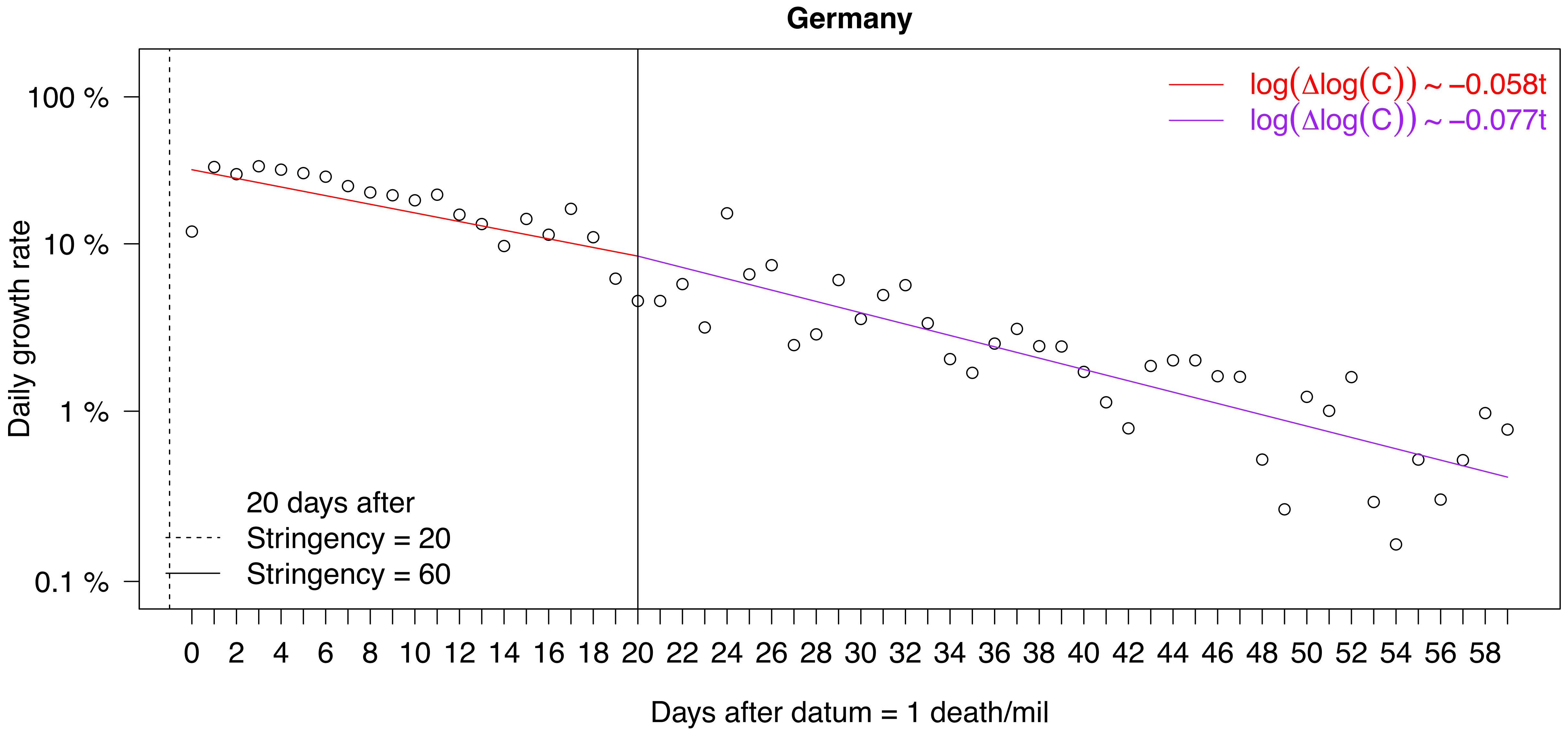

Germany
log(Δlog(C)) ~ −0.058t
log(Δlog(C)) ~ −0.077t
Daily growth rate
100 %
10 %
1 %
0.1 %
20 days after
Stringency = 20
Stringency = 60
0
2
4
6
8
10
12
14
16
18
20
22
24
26
28
30
32
34
36
38
40
42
44
46
48
50
52
54
56
58
Days after datum = 1 death/mil

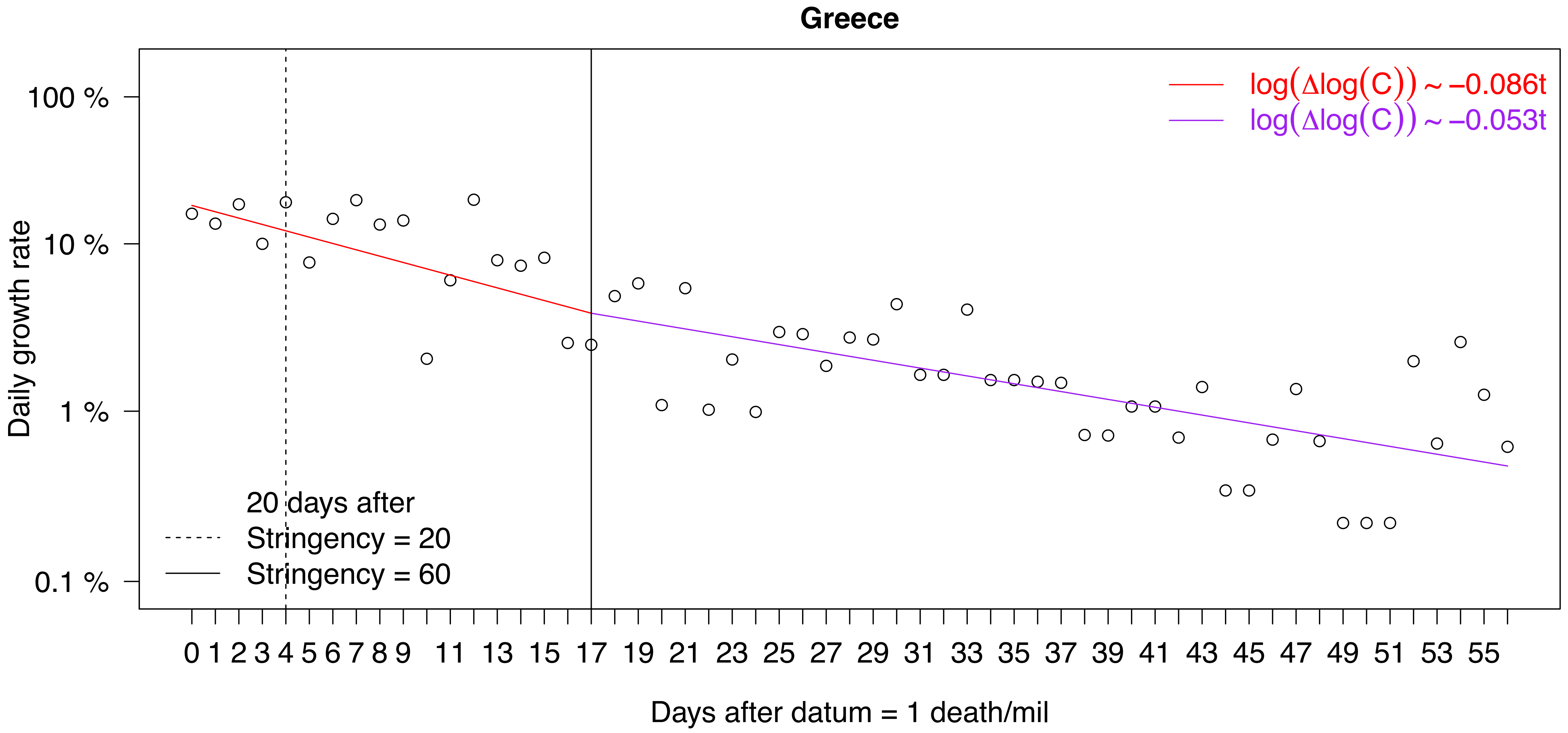

Greece
log(Δlog(C)) ~ −0.086t
log(Δlog(C)) ~ −0.053t
100 %
10 %
1 %
0.1 %
Daily growth rate
20 days after
Stringency = 20
Stringency = 60
0 1 2 3 4 5 6 7 8 9 11 13 15 17 19 21 23 25 27 29 31 33 35 37 39 41 43 45 47 49 51 53 55
Days after datum = 1 death/mil

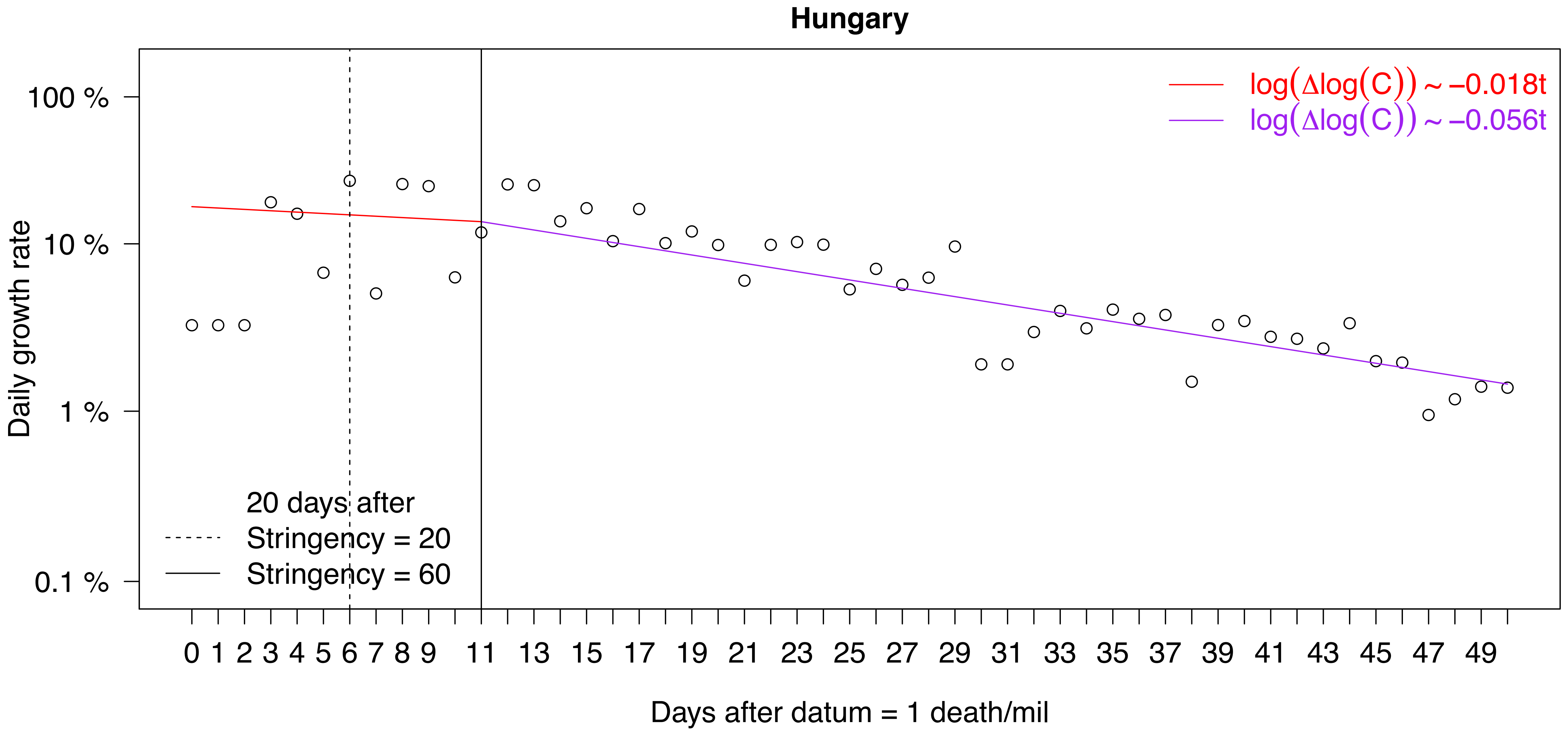

Hungary
log(Δlog(C)) ~ −0.018t
log(Δlog(C)) ~ −0.056t
100 %
10 %
1 %
0.1 %
Daily growth rate
20 days after
Stringency = 20
Stringency = 60
0 1 2 3 4 5 6 7 8 9 11 13 15 17 19 21 23 25 27 29 31 33 35 37 39 41 43 45 47 49
Days after datum = 1 death/mil

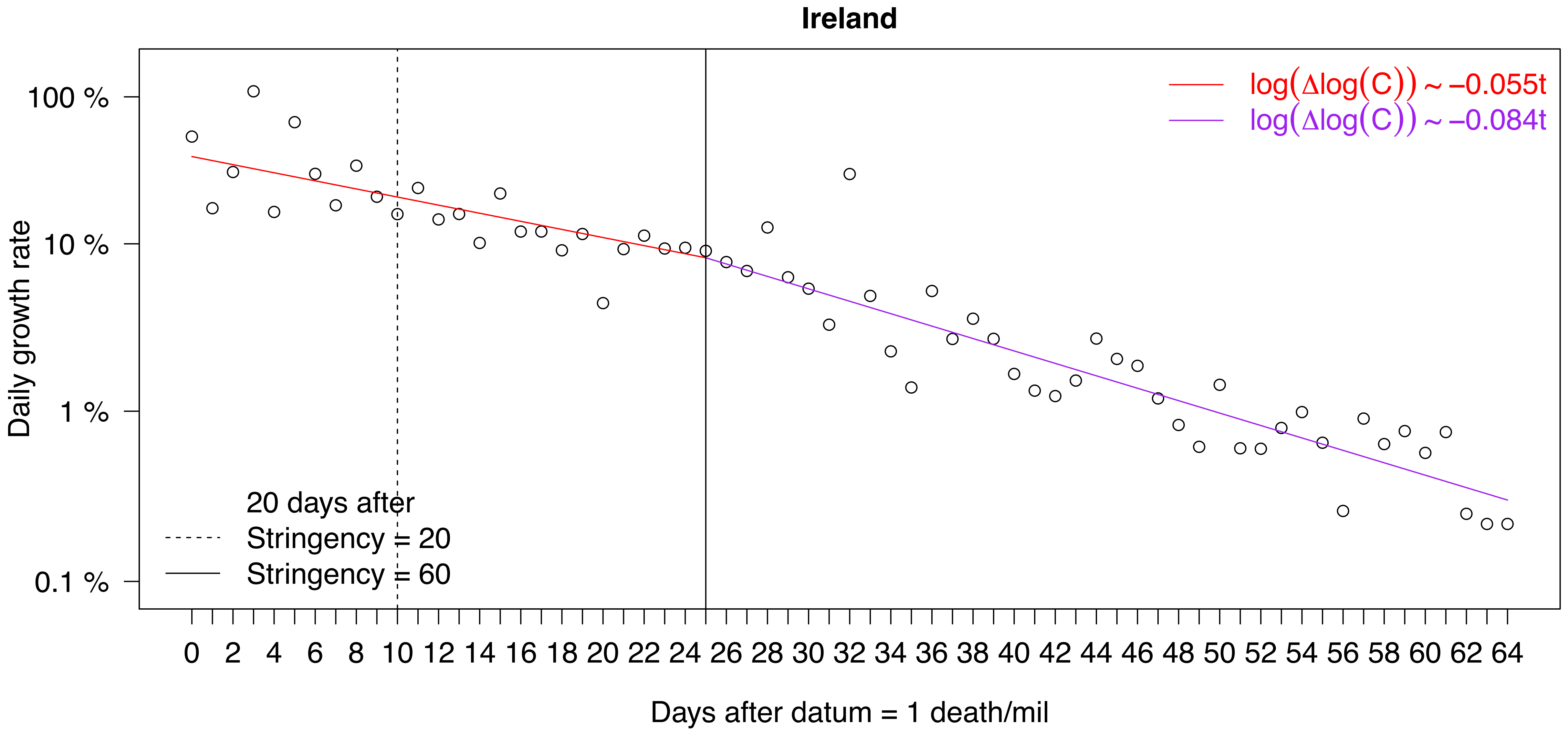
Ireland
log(Δlog(C)) ~ −0.055t
log(Δlog(C)) ~ −0.084t
100 %
10 %
1 %
0.1 %
Daily growth rate
20 days after
Stringency = 20
Stringency = 60
0 2 4 6 8 10 12 14 16 18 20 22 24 26 28 30 32 34 36 38 40 42 44 46 48 50 52 54 56 58 60 62 64
Days after datum = 1 death/mil

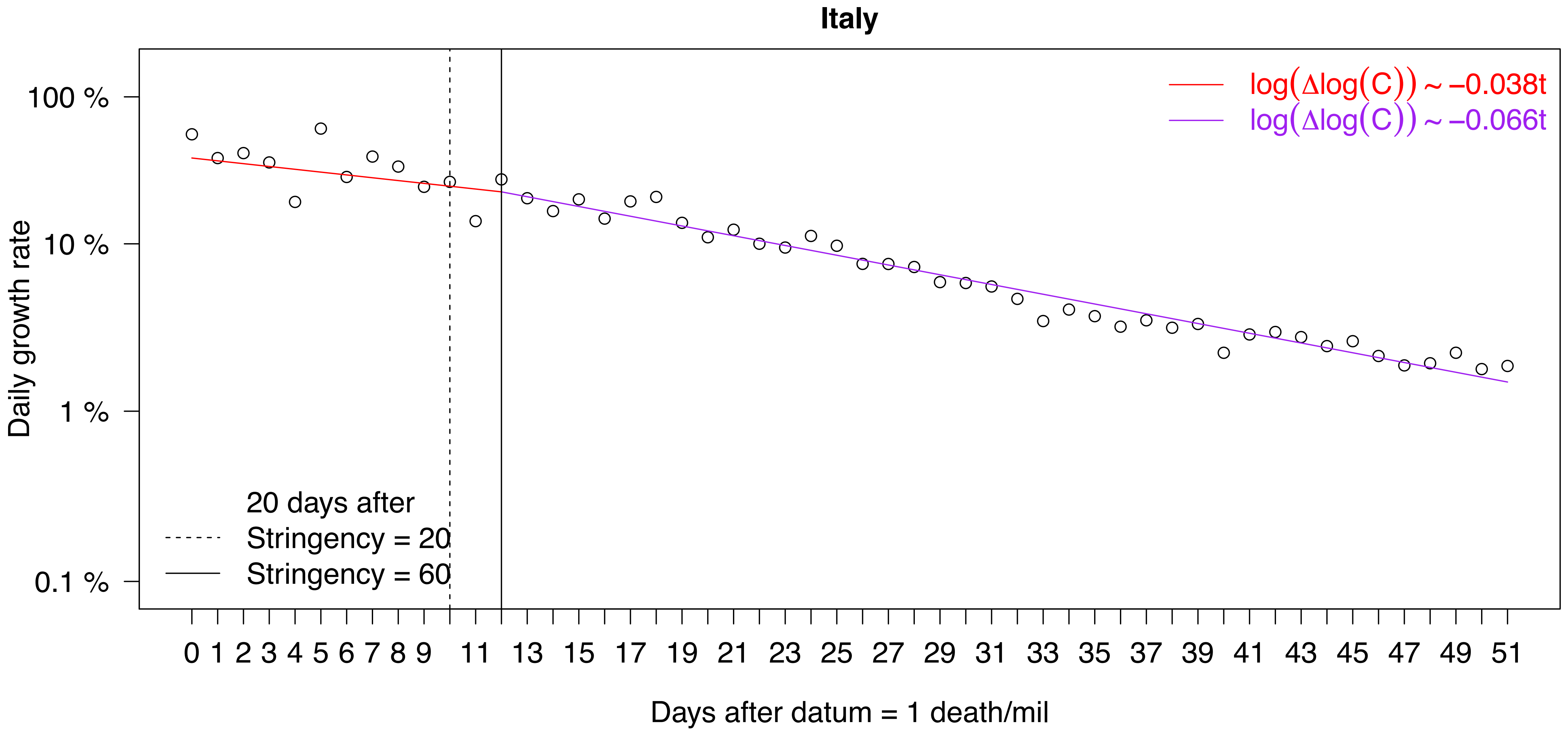

Italy
log(Δlog(C)) ~ −0.038t
log(Δlog(C)) ~ −0.066t
100 %
10 %
1 %
0.1 %
Daily growth rate
20 days after
Stringency = 20
Stringency = 60
0 1 2 3 4 5 6 7 8 9 11 13 15 17 19 21 23 25 27 29 31 33 35 37 39 41 43 45 47 49 51
Days after datum = 1 death/mil

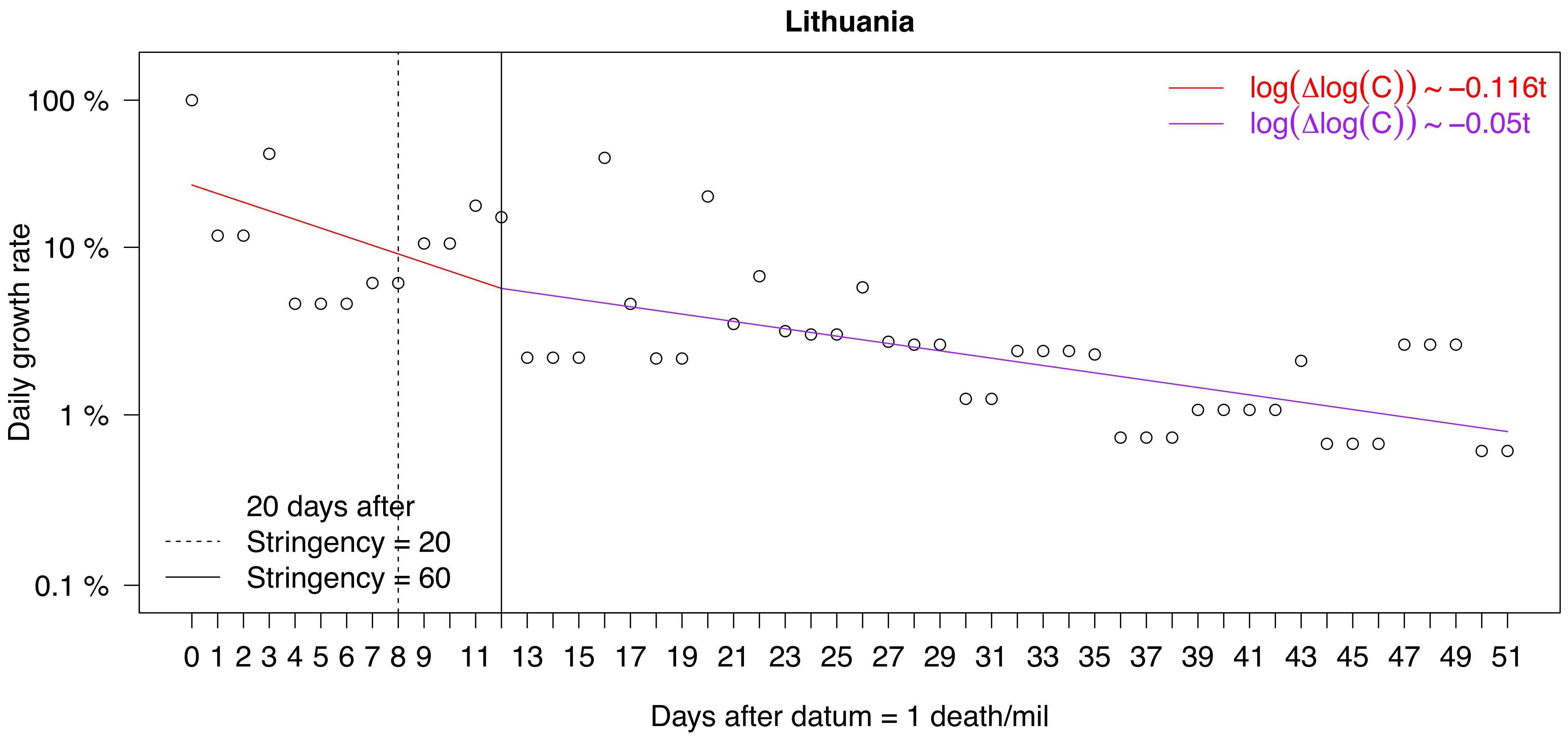

Lithuania
log(Δlog(C)) ~ −0.116t
log(Δlog(C)) ~ −0.05t
100 %
10 %
1 %
0.1 %
Daily growth rate
20 days after
Stringency = 20
Stringency = 60
0 1 2 3 4 5 6 7 8 9 11 13 15 17 19 21 23 25 27 29 31 33 35 37 39 41 43 45 47 49 51
Days after datum = 1 death/mil

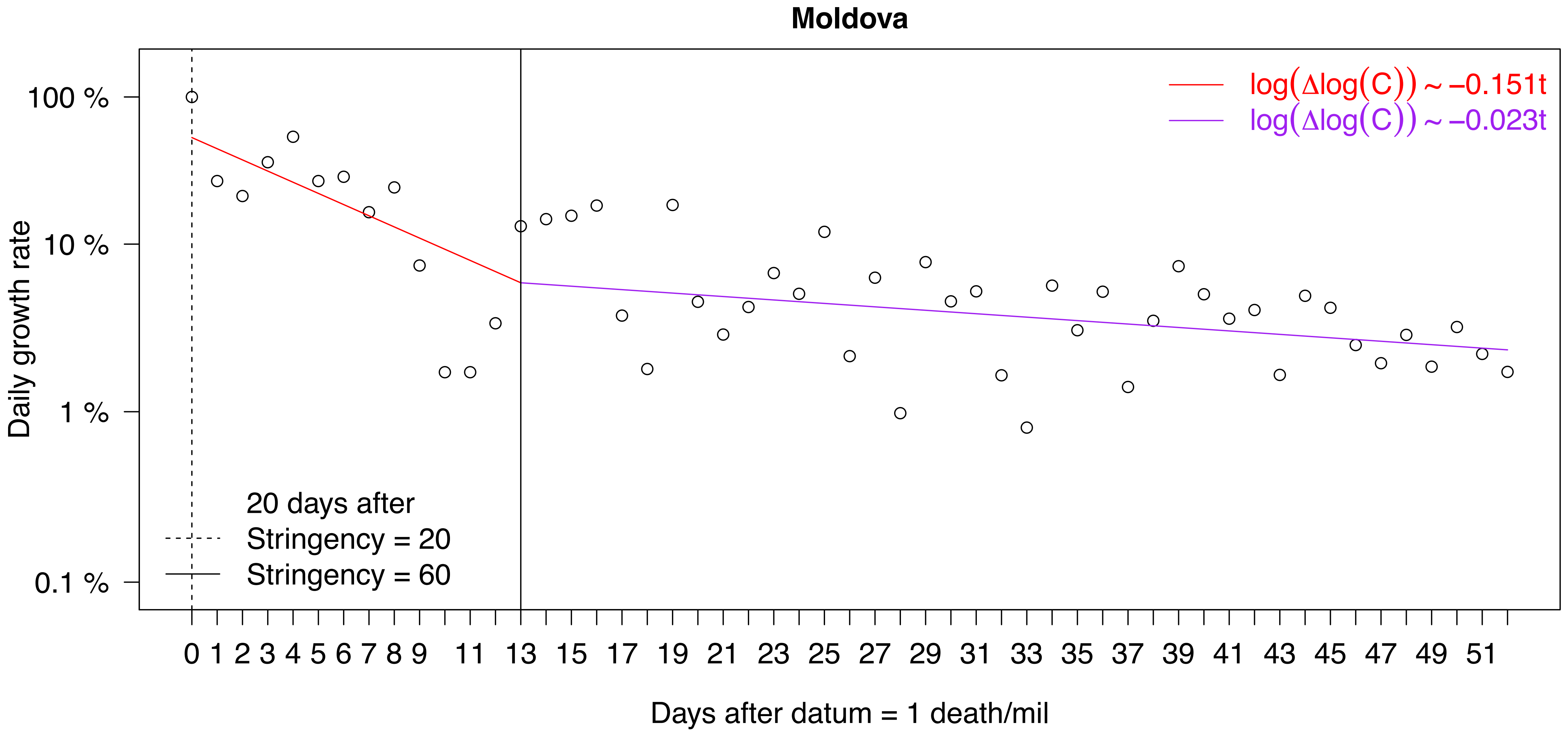

Moldova
log(Δlog(C)) ~ −0.151t
log(Δlog(C)) ~ −0.023t
Daily growth rate
100 %
10 %
1 %
0.1 %
20 days after
Stringency = 20
Stringency = 60
0 1 2 3 4 5 6 7 8 9 11 13 15 17 19 21 23 25 27 29 31 33 35 37 39 41 43 45 47 49 51
Days after datum = 1 death/mil

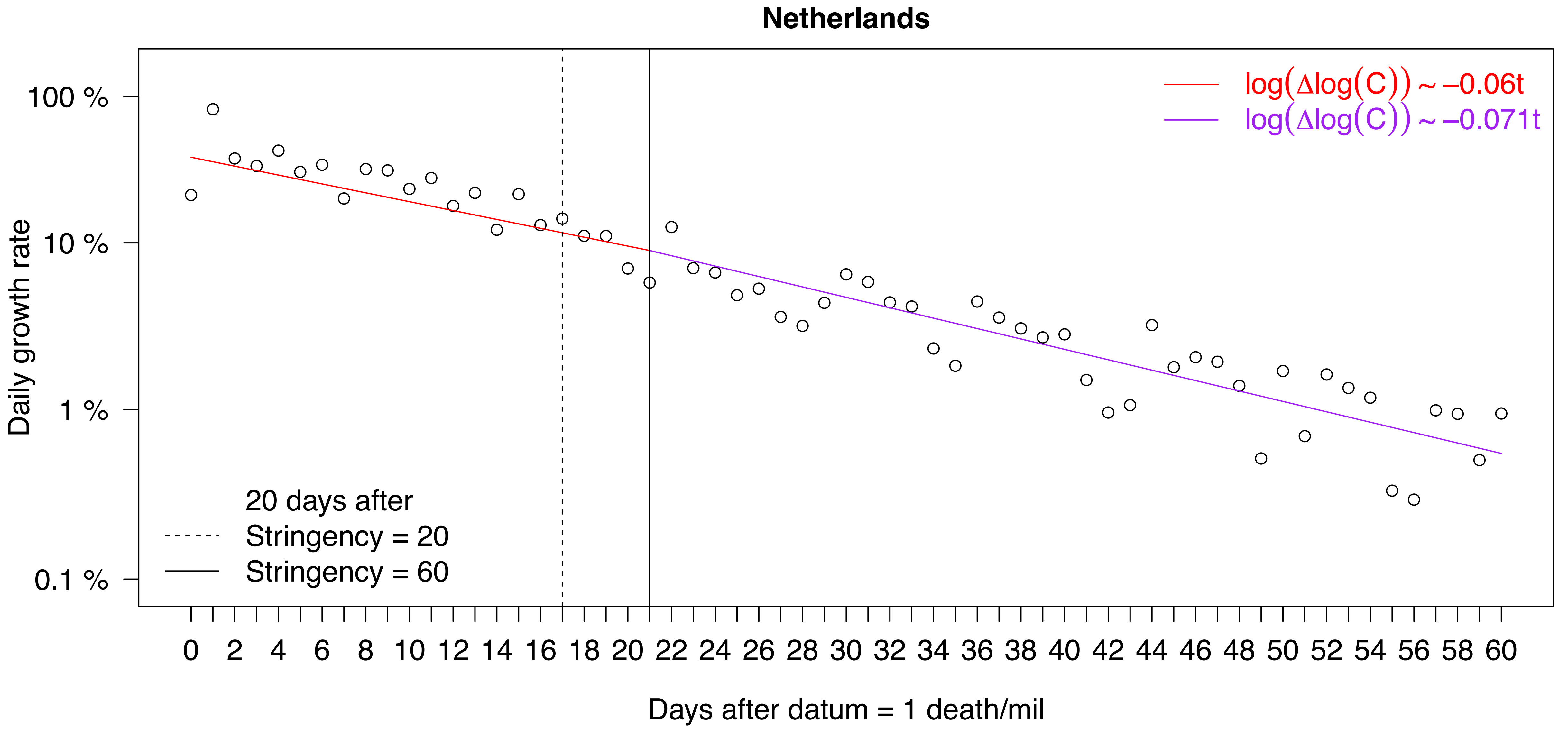

Netherlands
log(Δlog(C)) ~ −0.06t
log(Δlog(C)) ~ −0.071t
100 %
10 %
1 %
0.1 %
Daily growth rate
20 days after
Stringency = 20
Stringency = 60
0
2
4
6
8
10
12
14
16
18
20
22
24
26
28
30
32
34
36
38
40
42
44
46
48
50
52
54
56
58
60
Days after datum = 1 death/mil

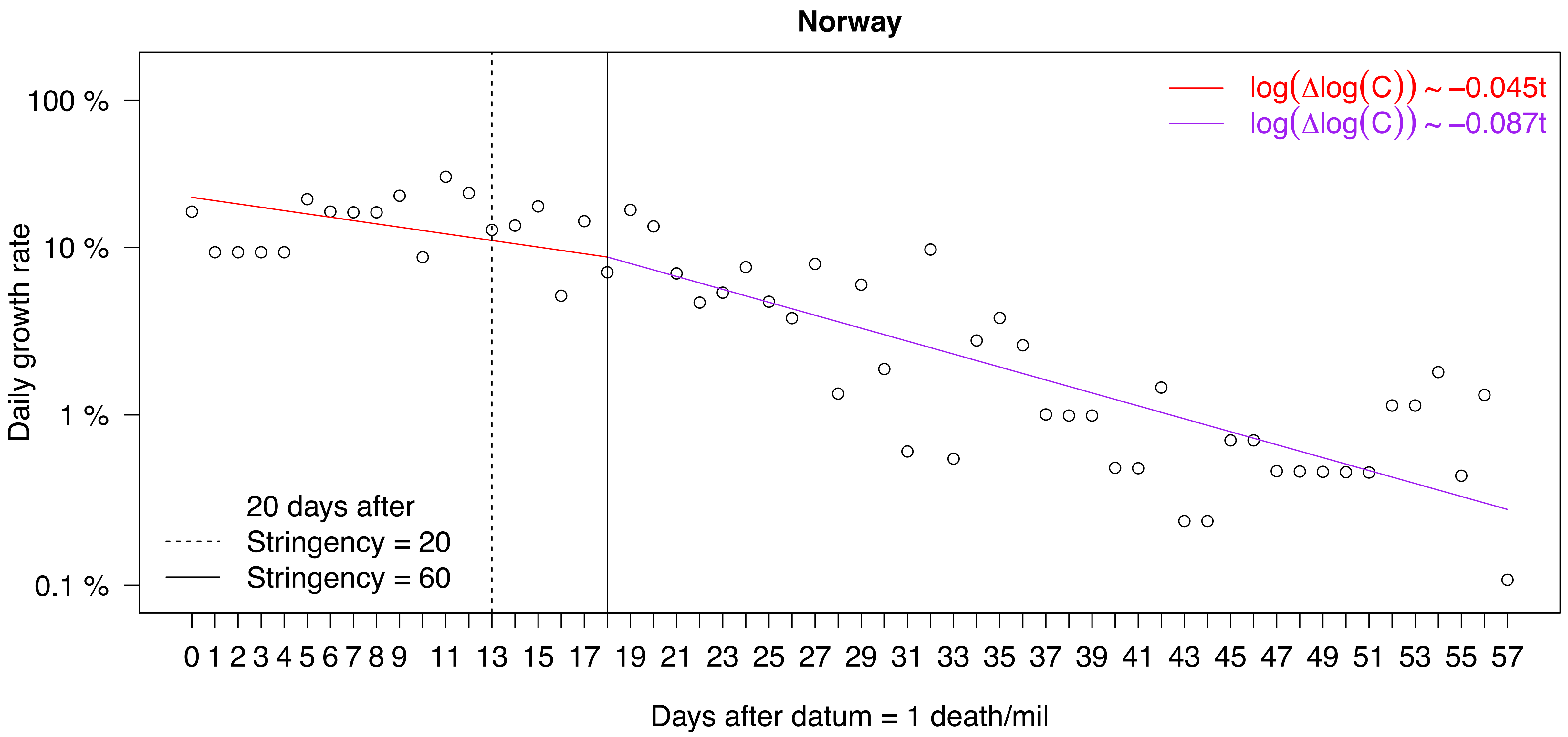

Norway
log(Δlog(C)) ~ −0.045t
log(Δlog(C)) ~ −0.087t
100 %
10 %
1 %
0.1 %
Daily growth rate
20 days after
Stringency = 20
Stringency = 60
0 1 2 3 4 5 6 7 8 9 11 13 15 17 19 21 23 25 27 29 31 33 35 37 39 41 43 45 47 49 51 53 55 57
Days after datum = 1 death/mil

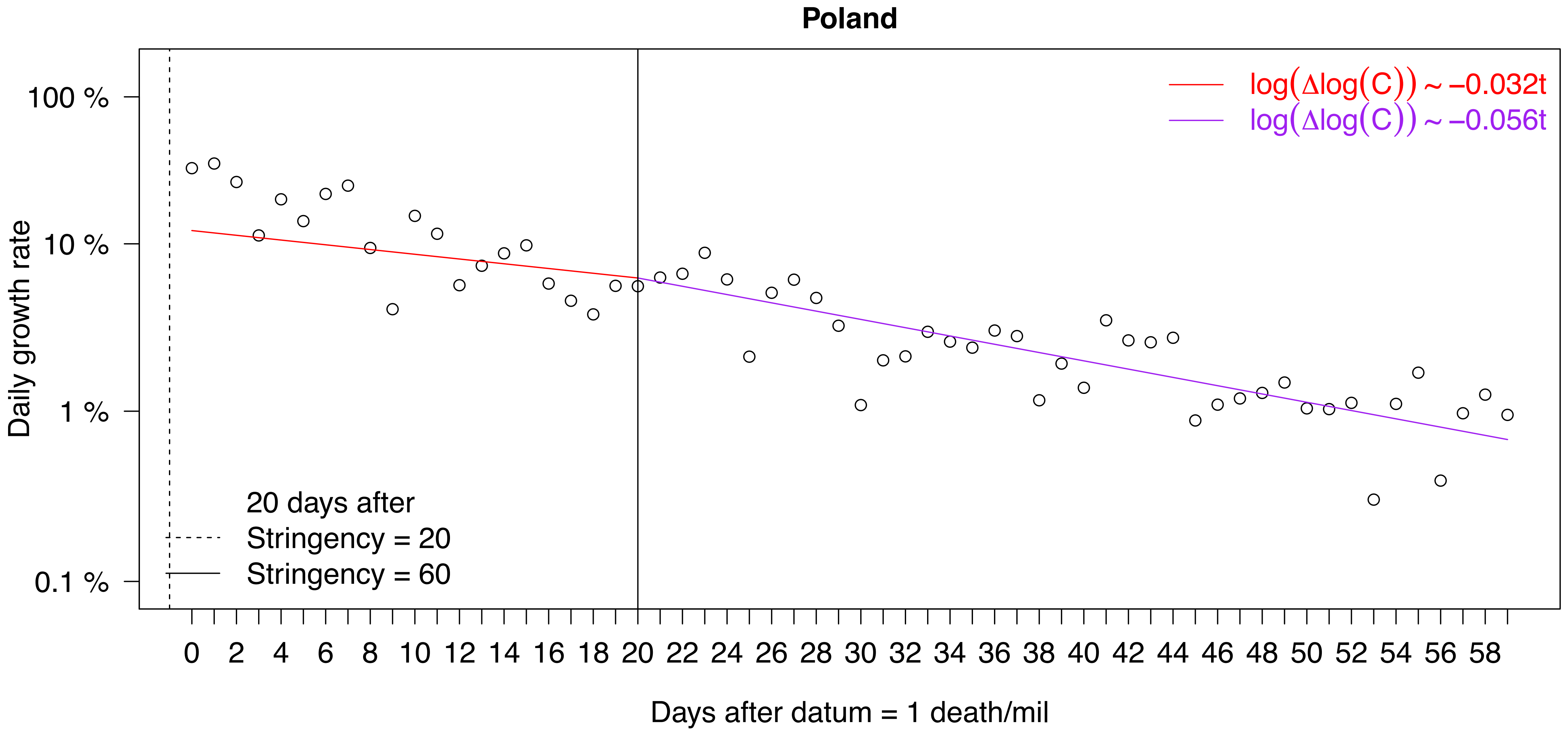
Poland
log(Δlog(C)) ~ −0.032t
log(Δlog(C)) ~ −0.056t
100 %
10 %
1 %
0.1 %
Daily growth rate
20 days after
Stringency = 20
Stringency = 60
0 2 4 6 8 10 12 14 16 18 20 22 24 26 28 30 32 34 36 38 40 42 44 46 48 50 52 54 56 58
Days after datum = 1 death/mil

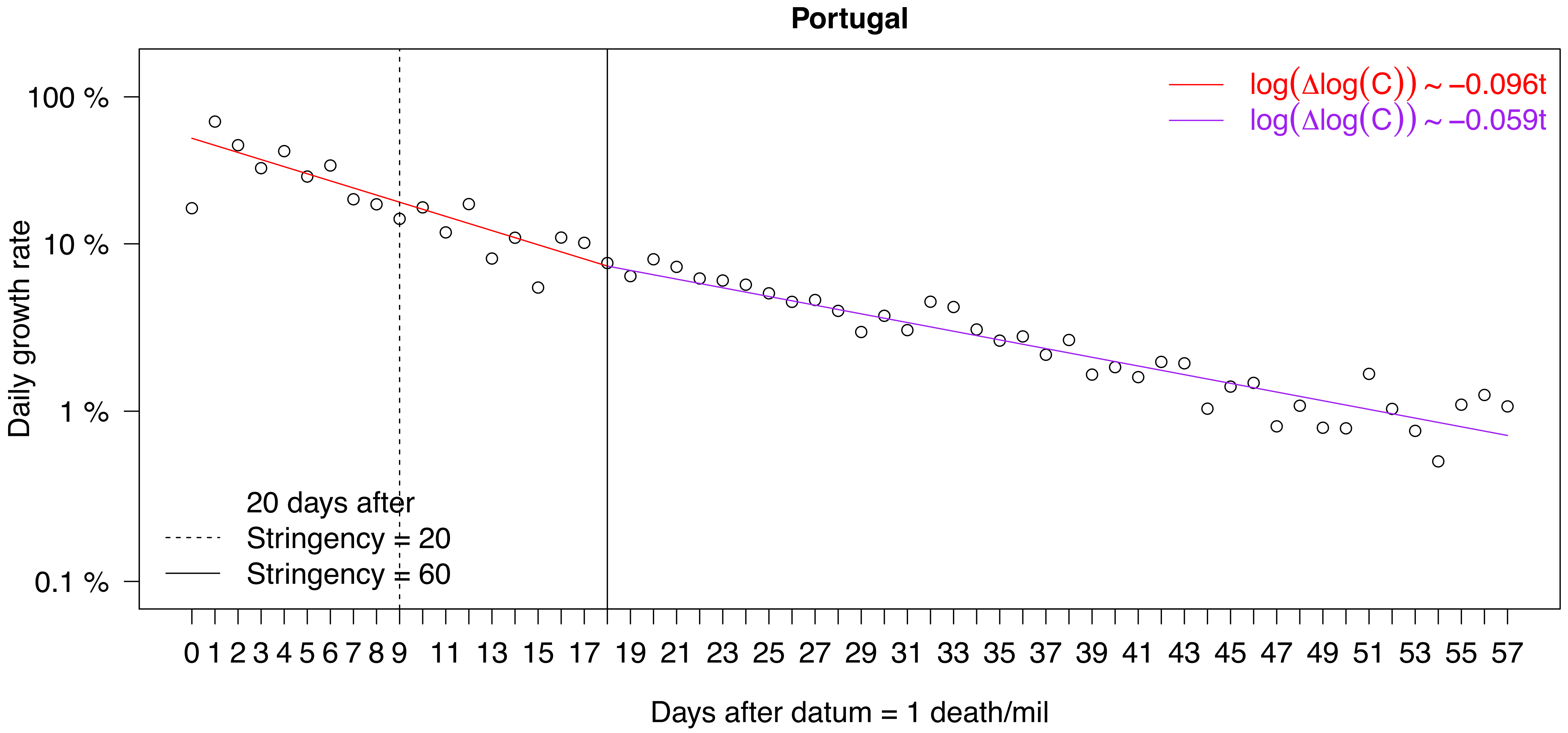

Portugal
Daily growth rate
100 %
10 %
1 %
0.1 %
log(Δlog(C)) ~ −0.096t
log(Δlog(C)) ~ −0.059t
20 days after
Stringency = 20
Stringency = 60
0 1 2 3 4 5 6 7 8 9 11 13 15 17 19 21 23 25 27 29 31 33 35 37 39 41 43 45 47 49 51 53 55 57
Days after datum = 1 death/mil

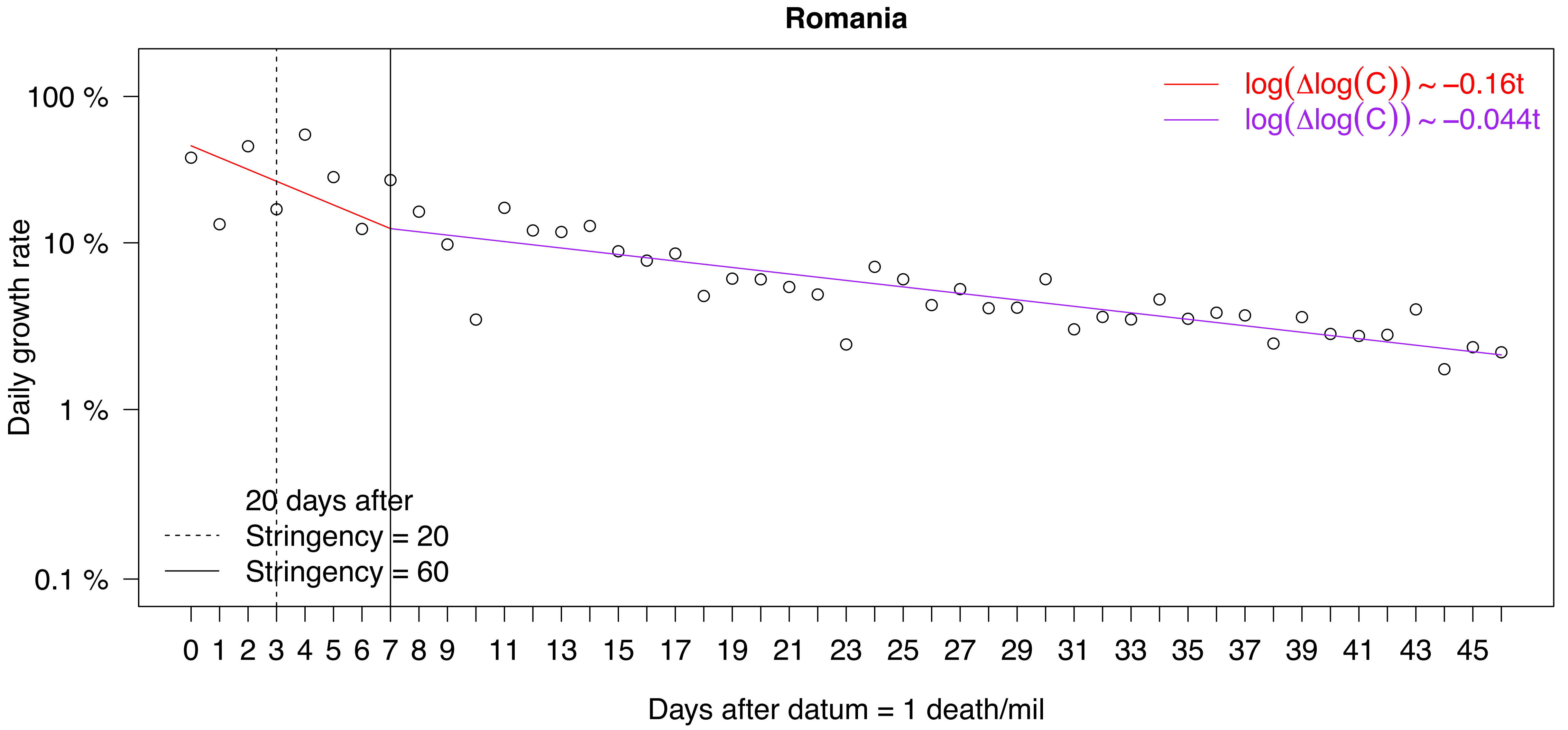

Romania
Daily growth rate
100 %
10 %
1 %
0.1 %
log(Δlog(C)) ~ −0.16t
log(Δlog(C)) ~ −0.044t
20 days after
Stringency = 20
Stringency = 60
0 1 2 3 4 5 6 7 8 9 11 13 15 17 19 21 23 25 27 29 31 33 35 37 39 41 43 45
Days after datum = 1 death/mil

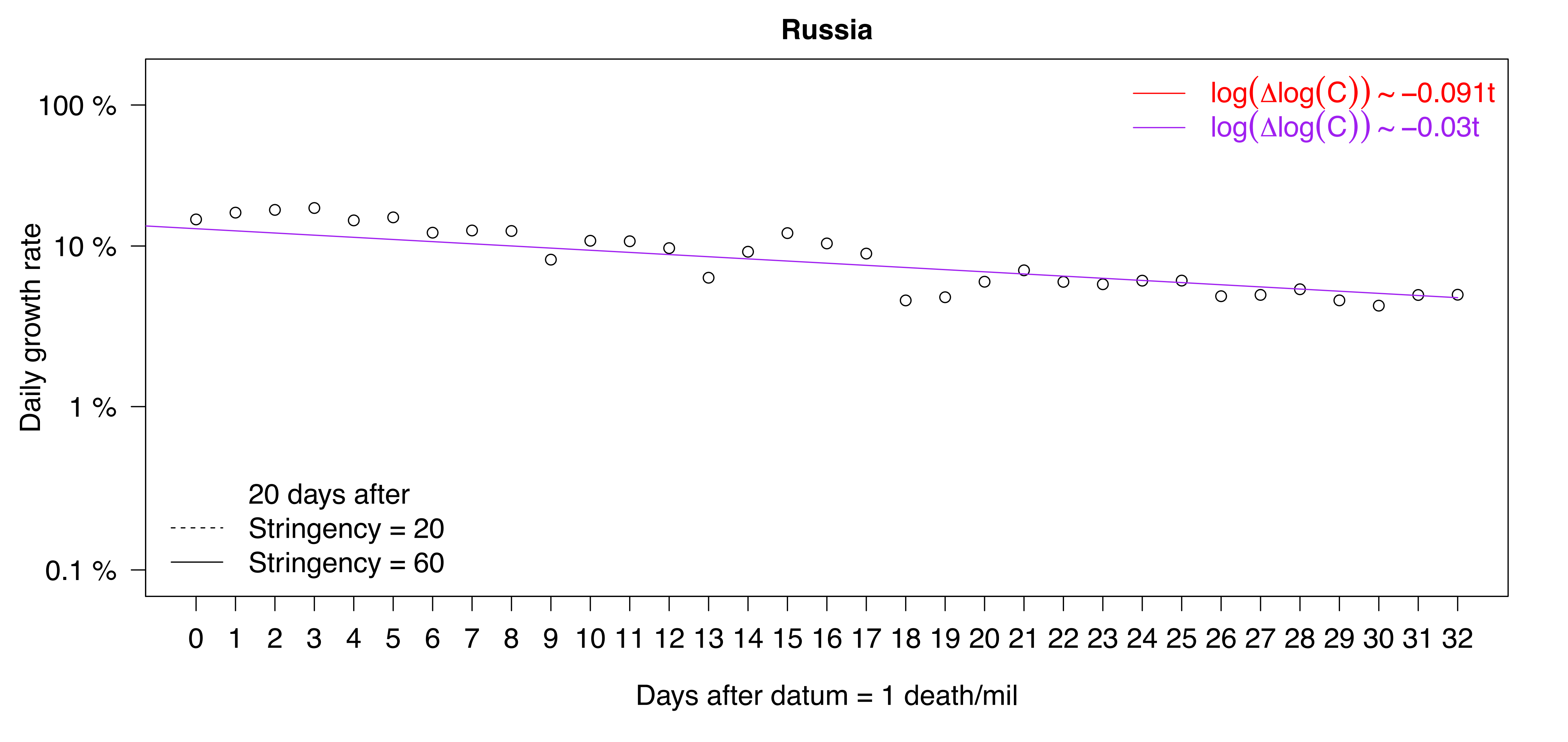
Russia
log(Δlog(C)) ~ −0.091t
log(Δlog(C)) ~ −0.03t
100 %
10 %
1 %
0.1 %
Daily growth rate
20 days after
Stringency = 20
Stringency = 60
0 1 2 3 4 5 6 7 8 9 10 11 12 13 14 15 16 17 18 19 20 21 22 23 24 25 26 27 28 29 30 31 32
Days after datum = 1 death/mil

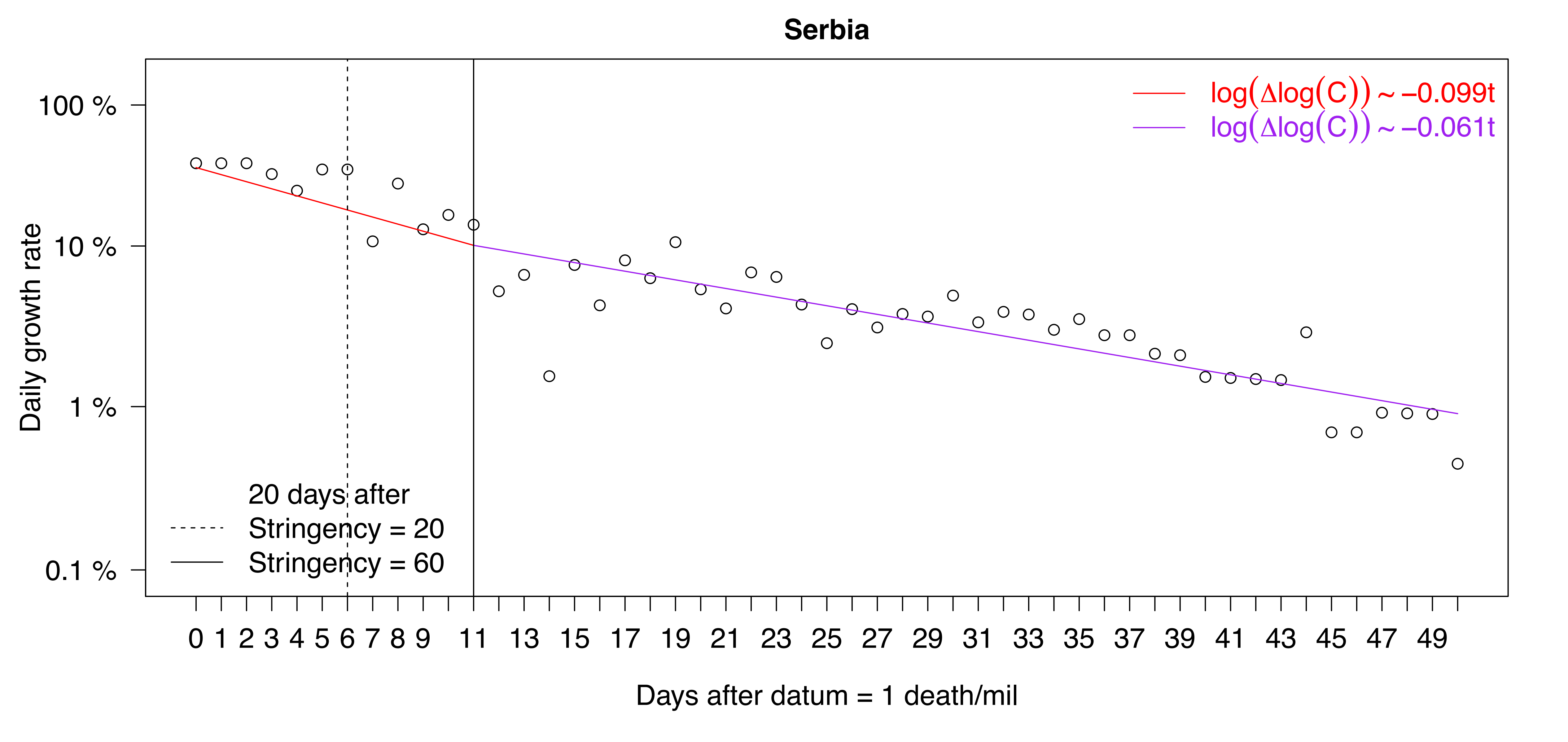
Serbia
log(Δlog(C)) ~ −0.099t
log(Δlog(C)) ~ −0.061t
Daily growth rate
100 %
10 %
1 %
0.1 %
20 days after
Stringency = 20
Stringency = 60
0 1 2 3 4 5 6 7 8 9 11 13 15 17 19 21 23 25 27 29 31 33 35 37 39 41 43 45 47 49
Days after datum = 1 death/mil

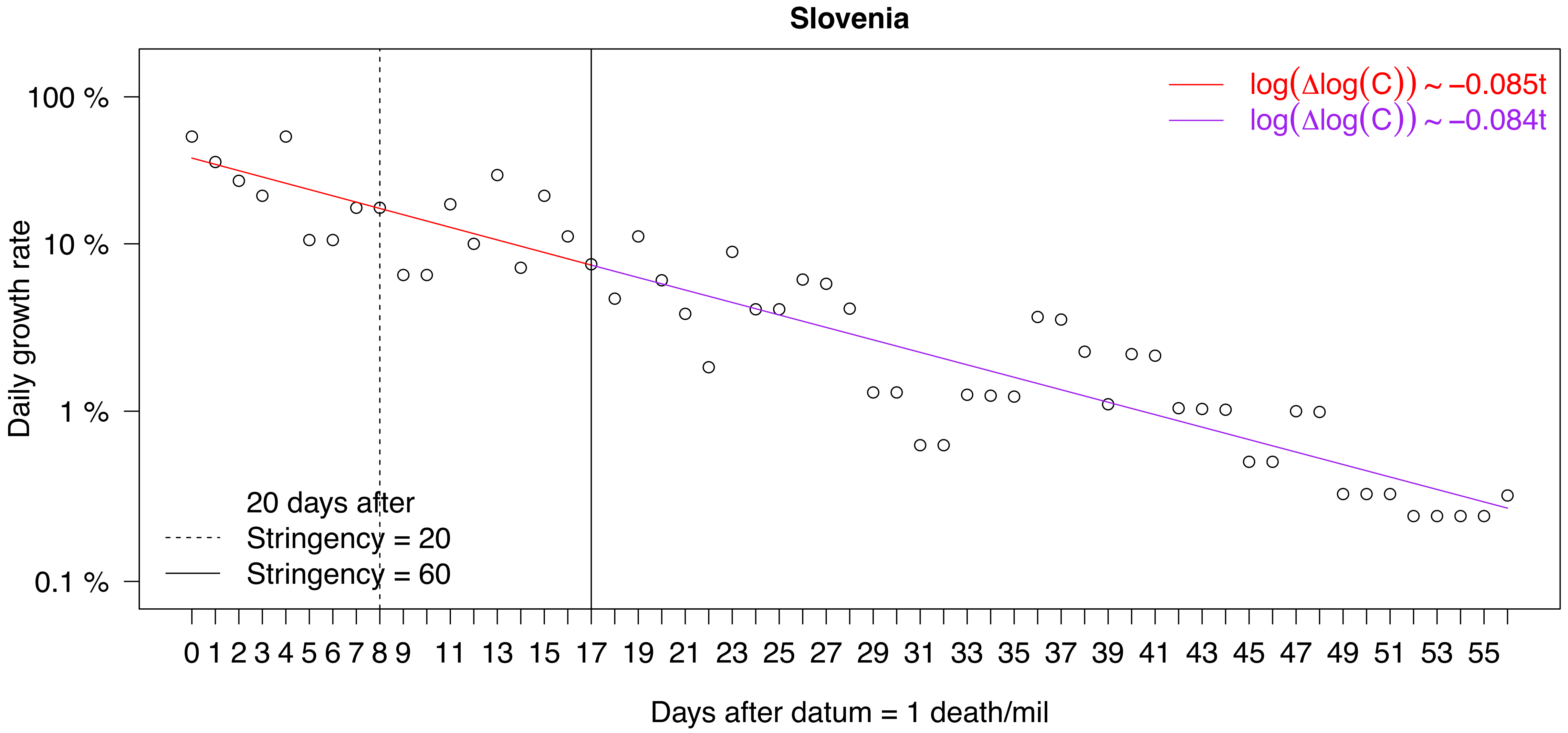

Slovenia
log(Δlog(C)) ~ −0.085t
log(Δlog(C)) ~ −0.084t
100 %
10 %
1 %
0.1 %
Daily growth rate
20 days after
Stringency = 20
Stringency = 60
0 1 2 3 4 5 6 7 8 9 11 13 15 17 19 21 23 25 27 29 31 33 35 37 39 41 43 45 47 49 51 53 55
Days after datum = 1 death/mil

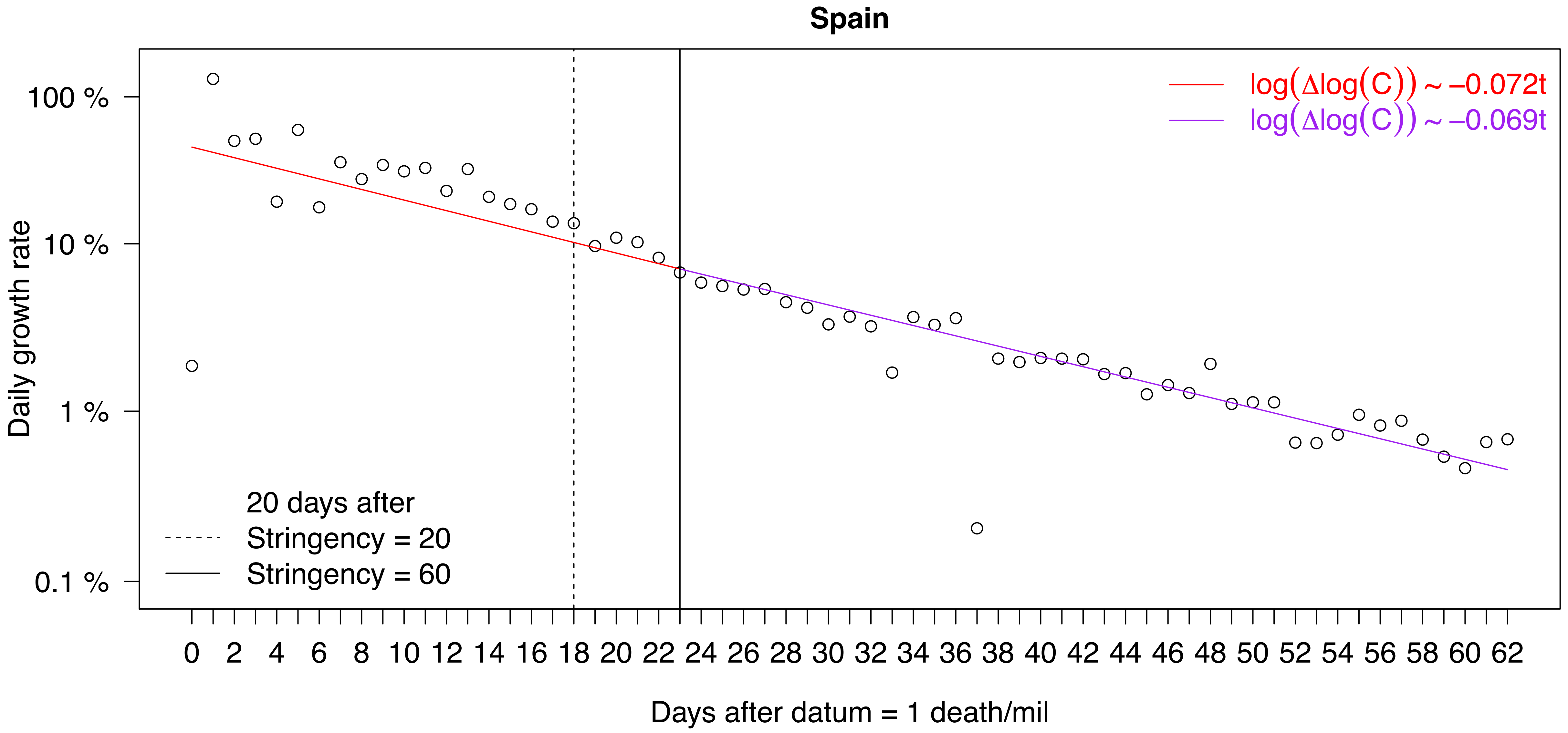

Spain
log(Δlog(C)) ~ −0.072t
log(Δlog(C)) ~ −0.069t
100 %
10 %
1 %
0.1 %
Daily growth rate
20 days after
Stringency = 20
Stringency = 60
0 2 4 6 8 10 12 14 16 18 20 22 24 26 28 30 32 34 36 38 40 42 44 46 48 50 52 54 56 58 60 62
Days after datum = 1 death/mil

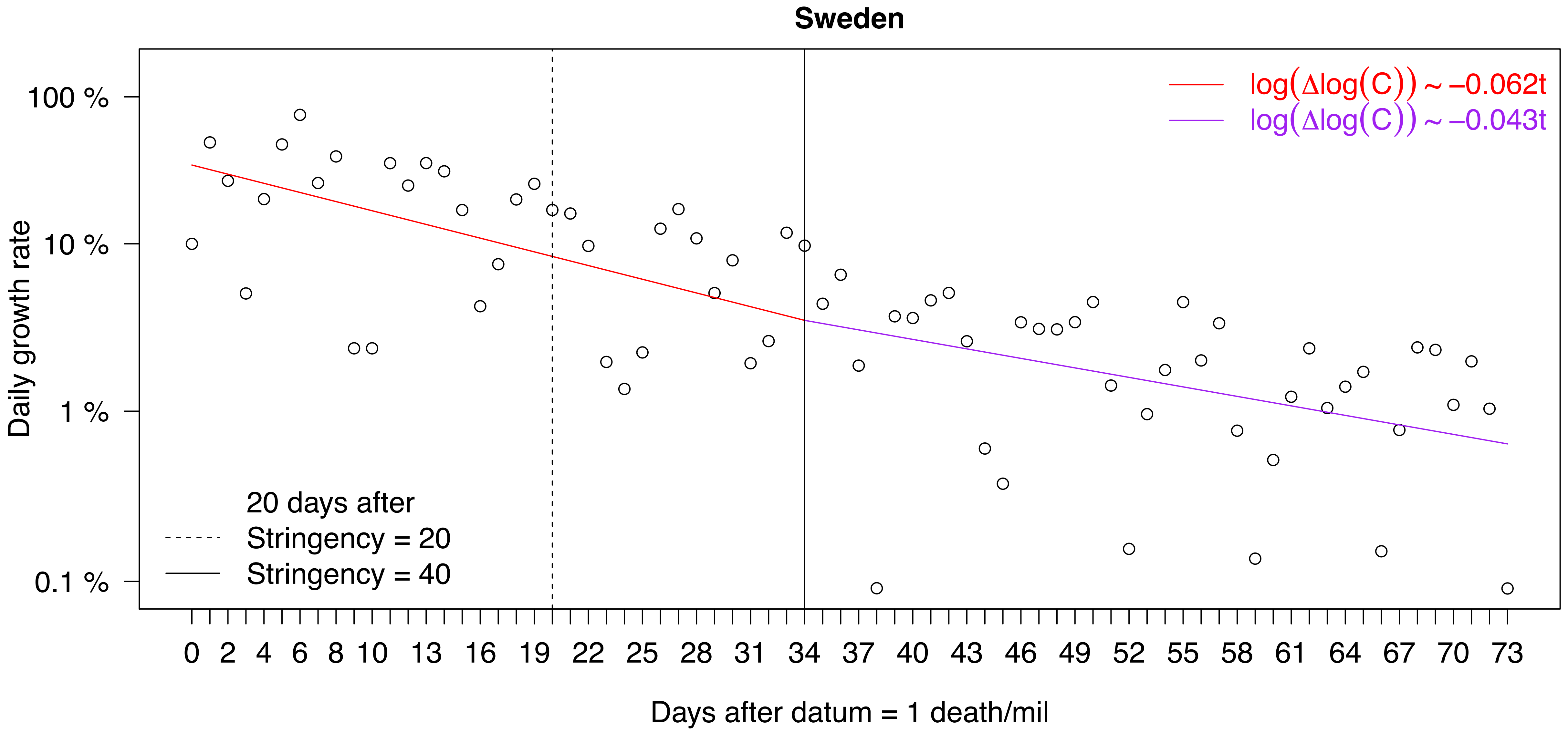
Sweden
Daily growth rate
100 %
10 %
1 %
0.1 %
log(Δlog(C)) ~ −0.062t
log(Δlog(C)) ~ −0.043t
20 days after
Stringency = 20
Stringency = 40
0 2 4 6 8 10 13 16 19 22 25 28 31 34 37 40 43 46 49 52 55 58 61 64 67 70 73
Days after datum = 1 death/mil

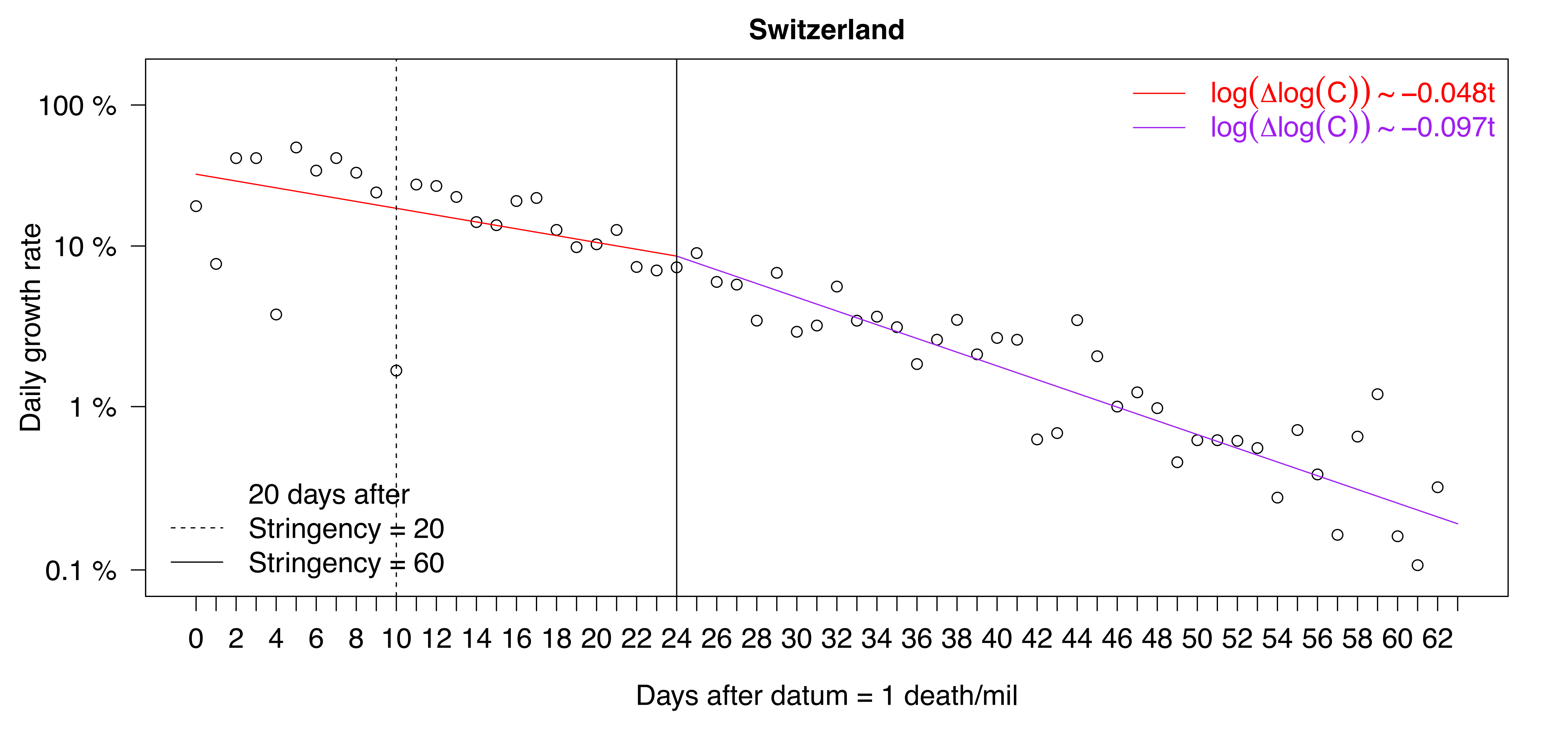
Switzerland
log(Δlog(C)) ~ −0.048t
log(Δlog(C)) ~ −0.097t
100 %
10 %
1 %
0.1 %
Daily growth rate
20 days after
Stringency = 20
Stringency = 60
0 2 4 6 8 10 12 14 16 18 20 22 24 26 28 30 32 34 36 38 40 42 44 46 48 50 52 54 56 58 60 62
Days after datum = 1 death/mil

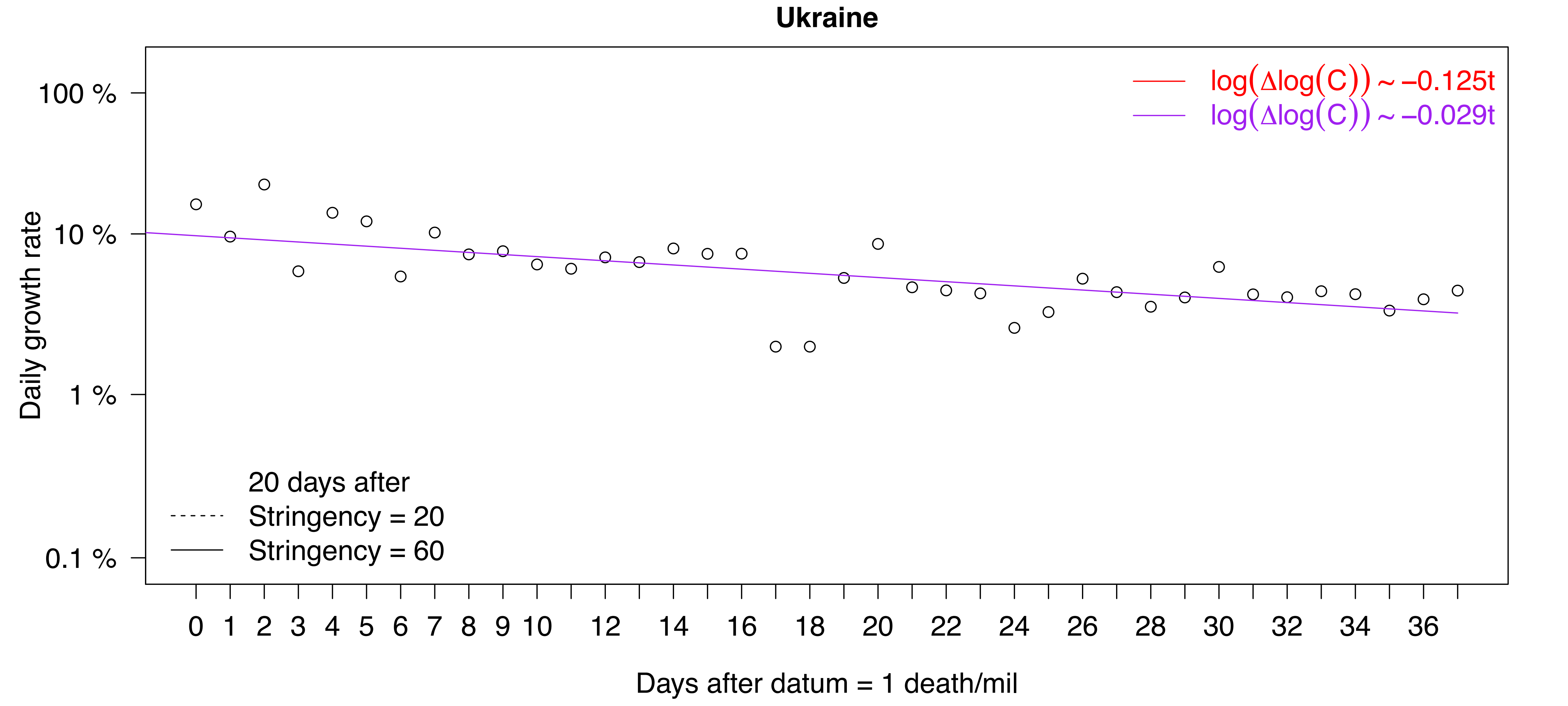

Ukraine
log(Δlog(C)) ~ −0.125t
log(Δlog(C)) ~ −0.029t
100 %
10 %
1 %
0.1 %
Daily growth rate
20 days after
Stringency = 20
Stringency = 60
0 1 2 3 4 5 6 7 8 9 10 12 14 16 18 20 22 24 26 28 30 32 34 36
Days after datum = 1 death/mil

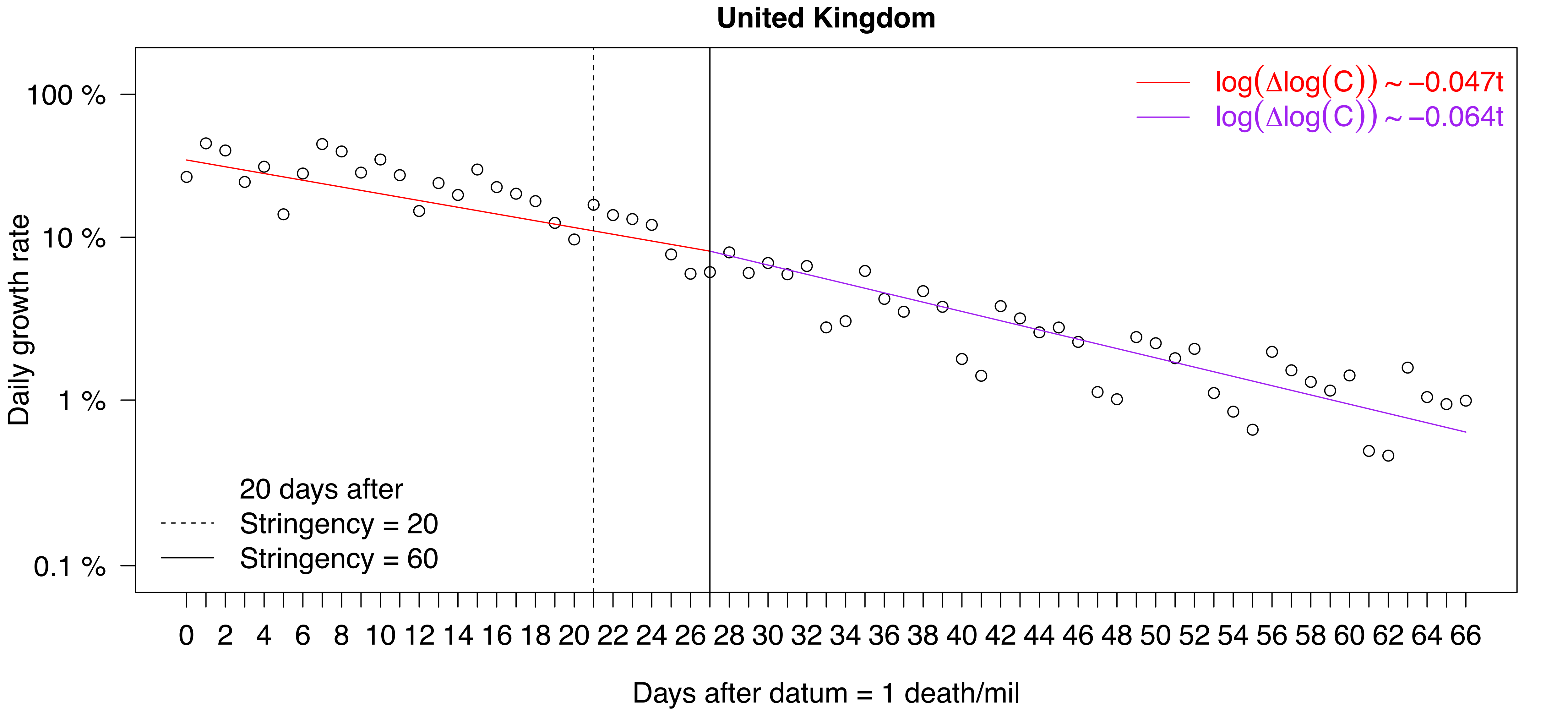
United Kingdom
log(Δlog(C)) ~ −0.047t
log(Δlog(C)) ~ −0.064t
100 %
10 %
1 %
0.1 %
Daily growth rate
20 days after
Stringency = 20
Stringency = 60
0 2 4 6 8 10 12 14 16 18 20 22 24 26 28 30 32 34 36 38 40 42 44 46 48 50 52 54 56 58 60 62 64 66
Days after datum = 1 death/mil

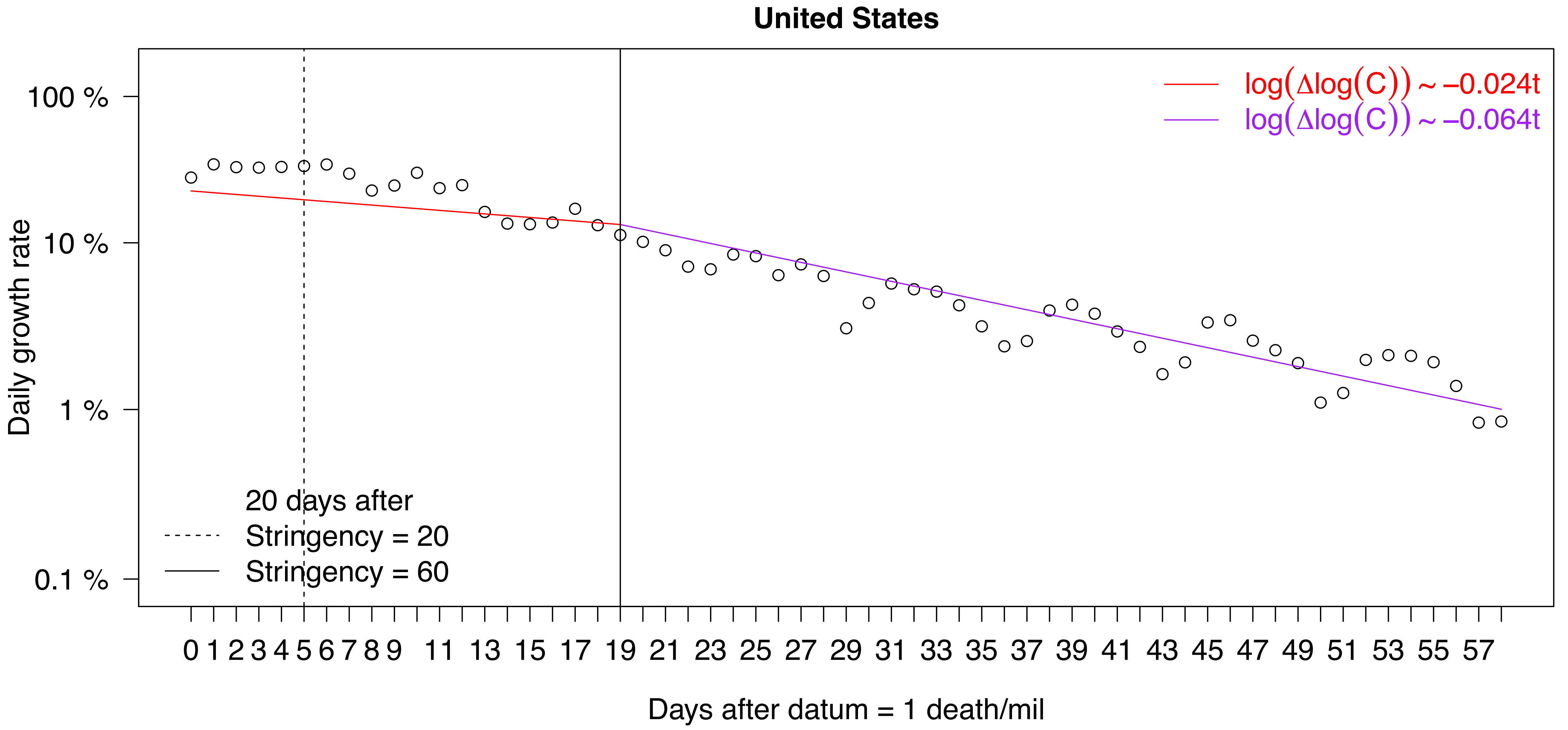
United States
log(Δlog(C)) ~ −0.024t
log(Δlog(C)) ~ −0.064t
Daily growth rate
100 %
10 %
1 %
0.1 %
20 days after
Stringency = 20
Stringency = 60
0 1 2 3 4 5 6 7 8 9 11 13 15 17 19 21 23 25 27 29 31 33 35 37 39 41 43 45 47 49 51 53 55 57
Days after datum = 1 death/mil
