# Supplementary material for: Interpreting, analysing and modelling COVID-19 mortality data
Source: Nonlinear Dyn. 2020 Oct 1;101(3):1751–76. doi: 10.1007/s11071-020-05966-z (PMC7527427; doi:10.1007/s11071-020-05966-z)
Supplement: Supplementary file 2 — Supplementary material 2 (pdf 7179 KB) [file 11071_2020_5966_MOESM2_ESM.pdf]

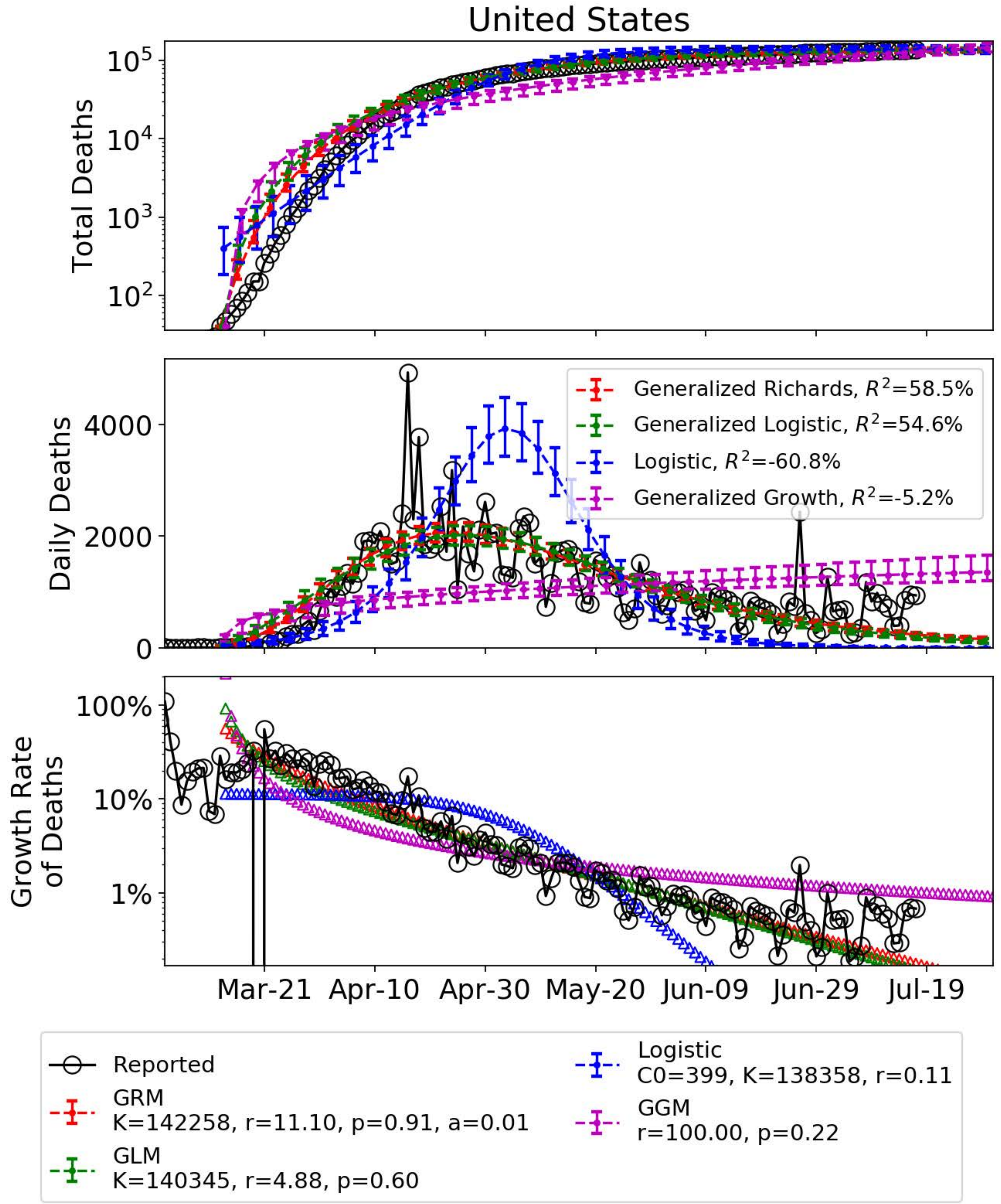

United States
Total Deaths
10^5
10^4
10^3
10^2
Daily Deaths
4000
2000
0
Generalized Richards, R^2=58.5%
Generalized Logistic, R^2=54.6%
Logistic, R^2=-60.8%
Generalized Growth, R^2=-5.2%
Growth Rate of Deaths
100%
10%
1%
Mar-21
Apr-10
Apr-30
May-20
Jun-09
Jun-29
Jul-19
Reported
GRM
K=142258, r=11.10, p=0.91, a=0.01
GLM
K=140345, r=4.88, p=0.60
Logistic
C0=399, K=138358, r=0.11
GGM
r=100.00, p=0.22

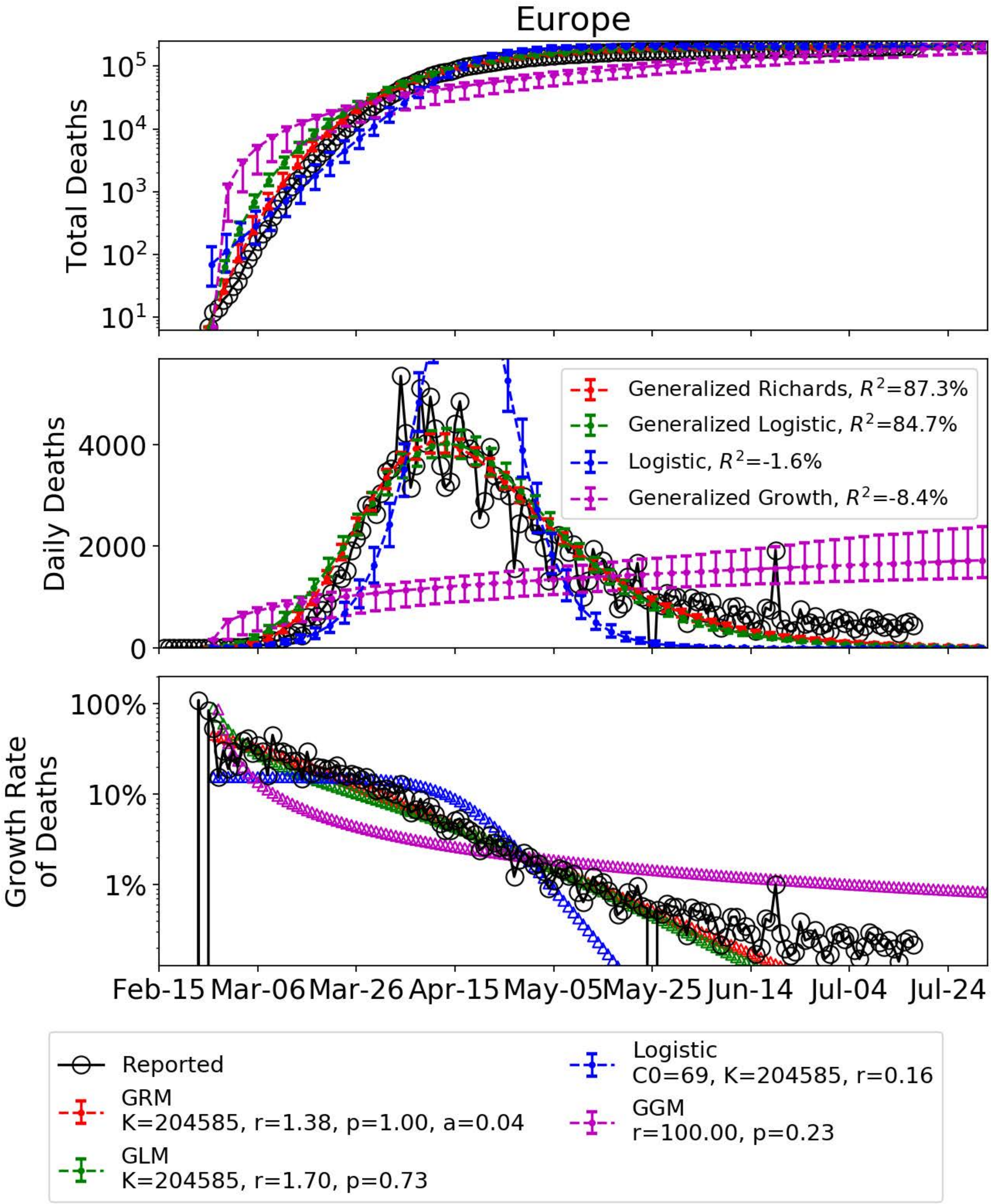

Europe
Total Deaths
10^1
10^2
10^3
10^4
10^5
Daily Deaths
0
2000
4000
Generalized Richards, R^2=87.3%
Generalized Logistic, R^2=84.7%
Logistic, R^2=-1.6%
Generalized Growth, R^2=-8.4%
Growth Rate of Deaths
1%
10%
100%
Feb-15
Mar-06
Mar-26
Apr-15
May-05
May-25
Jun-14
Jul-04
Jul-24
Reported
GRM
K=204585, r=1.38, p=1.00, a=0.04
GLM
K=204585, r=1.70, p=0.73
Logistic
C0=69, K=204585, r=0.16
GGM
r=100.00, p=0.23

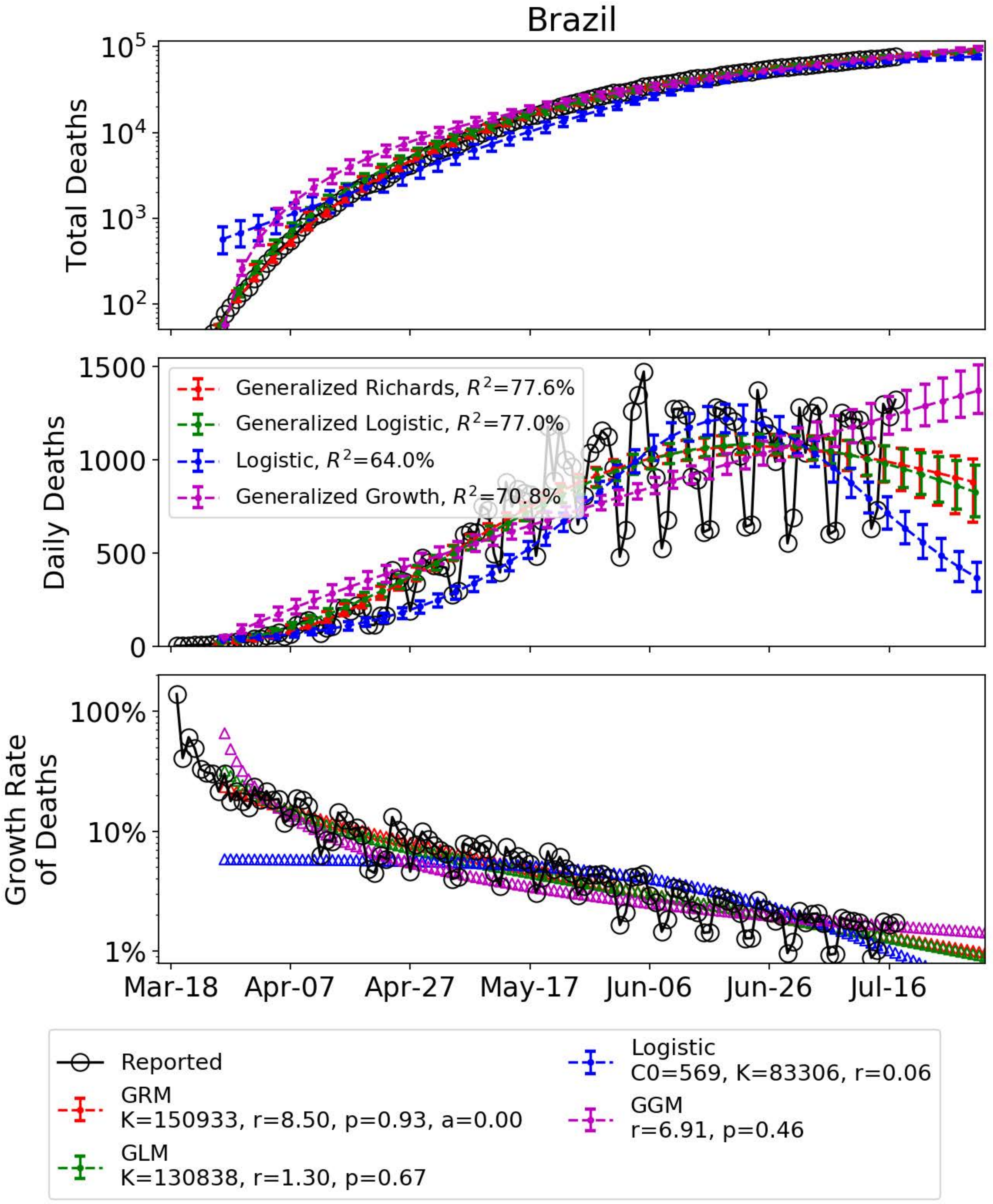
Brazil
Total Deaths
Daily Deaths
Growth Rate of Deaths
Generalized Richards, R^2=77.6%
Generalized Logistic, R^2=77.0%
Logistic, R^2=64.0%
Generalized Growth, R^2=70.8%
Mar-18
Apr-07
Apr-27
May-17
Jun-06
Jun-26
Jul-16
Reported
GRM
K=150933, r=8.50, p=0.93, a=0.00
GLM
K=130838, r=1.30, p=0.67
Logistic
C0=569, K=83306, r=0.06
GGM
r=6.91, p=0.46

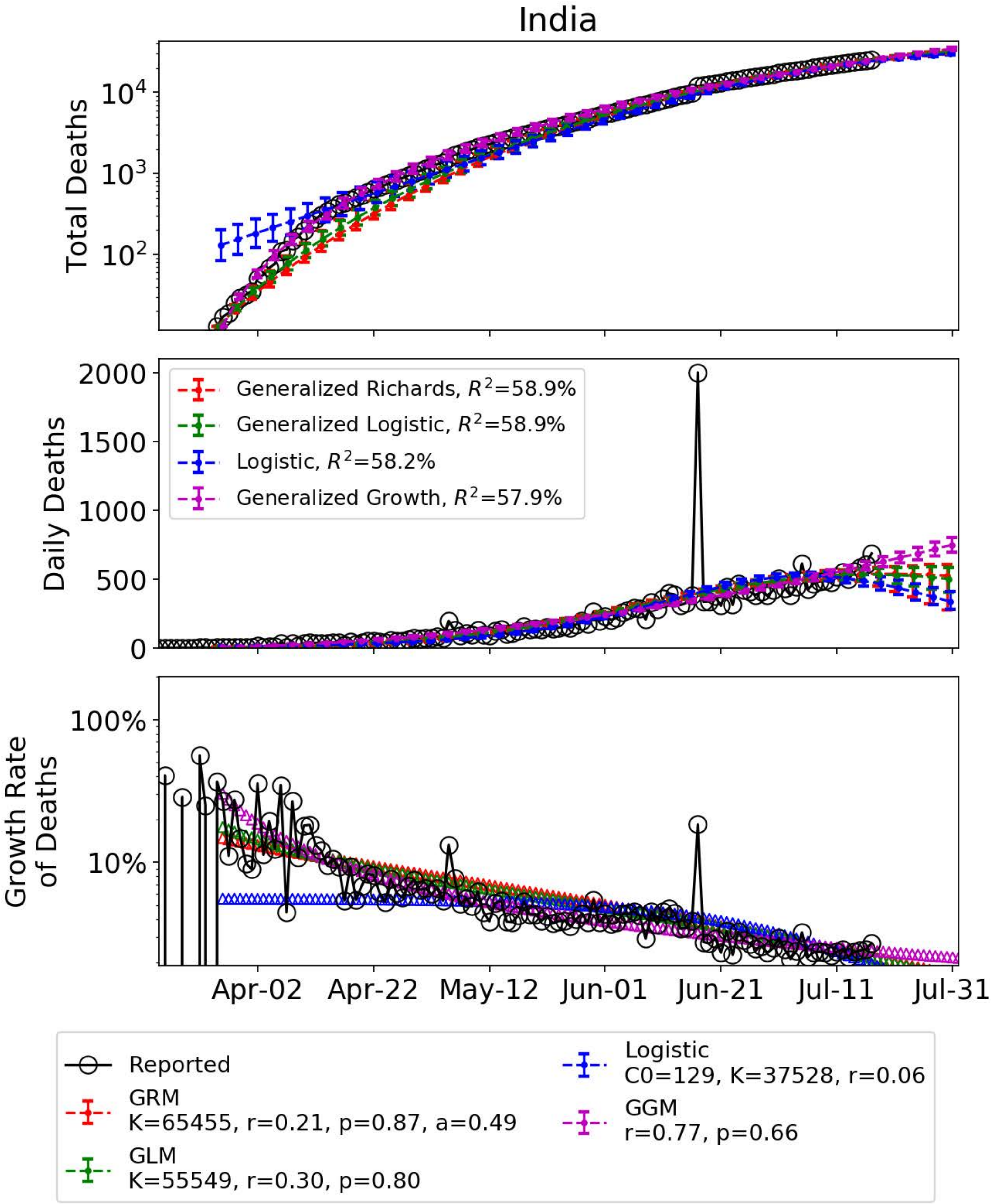

India
Total Deths
Total Deaths
10^2
10^3
10^4
Daily Deaths
0
500
1000
1500
2000
Generalized Richards, R^2=58.9%
Generalized Logistic, R^2=58.9%
Logistic, R^2=58.2%
Generalized Growth, R^2=57.9%
Growth Rate of Deaths
10%
100%
Apr-02
Apr-22
May-12
Jun-01
Jun-21
Jul-11
Jul-31
Reported
GRM
K=65455, r=0.21, p=0.87, a=0.49
GLM
K=55549, r=0.30, p=0.80
Logistic
C0=129, K=37528, r=0.06
GGM
r=0.77, p=0.66

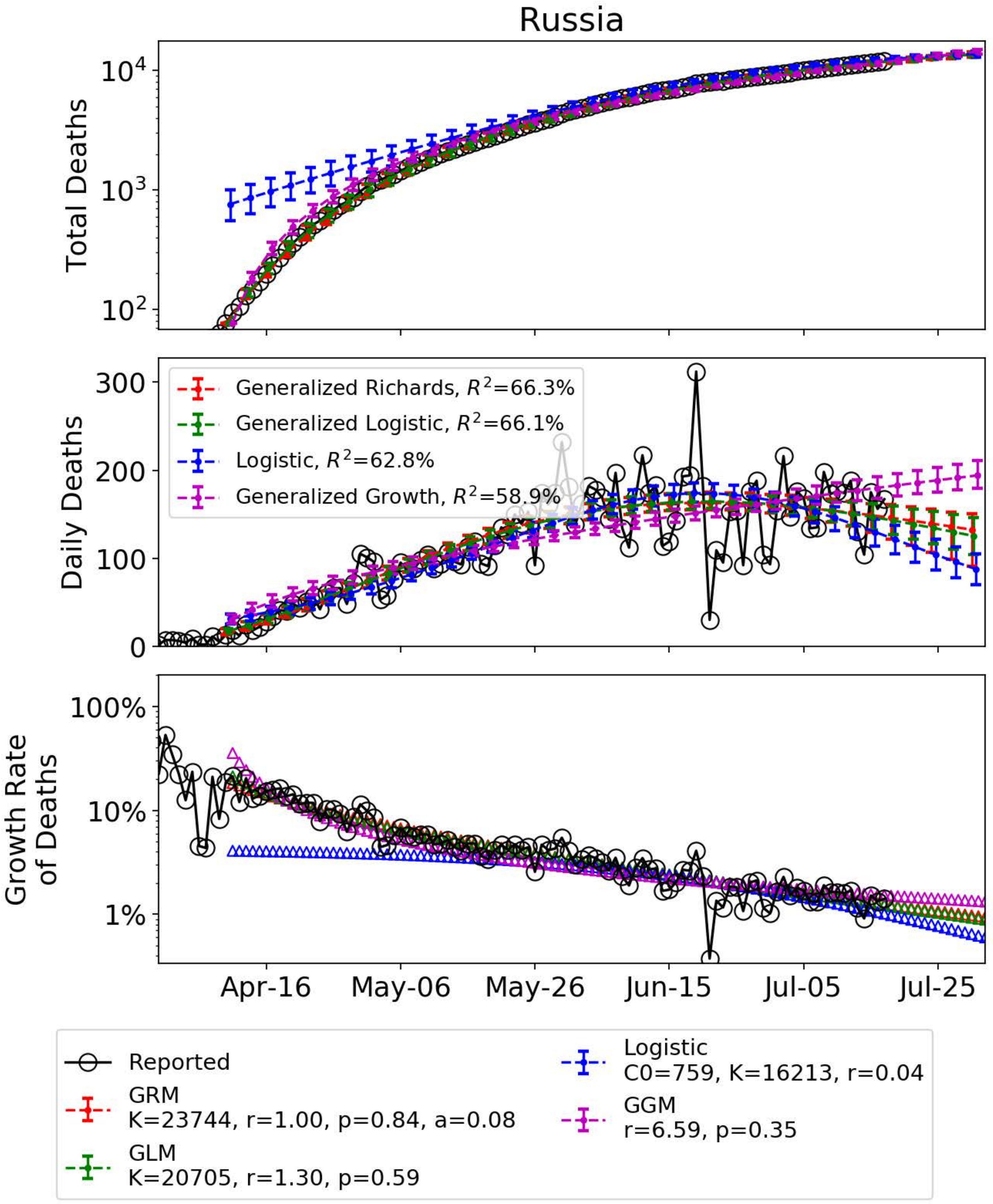
Russia
Total Deaths
Daily Deaths
Growth Rate of Deaths
Generalized Richards, R^2=66.3%
Generalized Logistic, R^2=66.1%
Logistic, R^2=62.8%
Generalized Growth, R^2=58.9%
Apr-16
May-06
May-26
Jun-15
Jul-05
Jul-25
Reported
GRM
K=23744, r=1.00, p=0.84, a=0.08
GLM
K=20705, r=1.30, p=0.59
Logistic
C0=759, K=16213, r=0.04
GGM
r=6.59, p=0.35

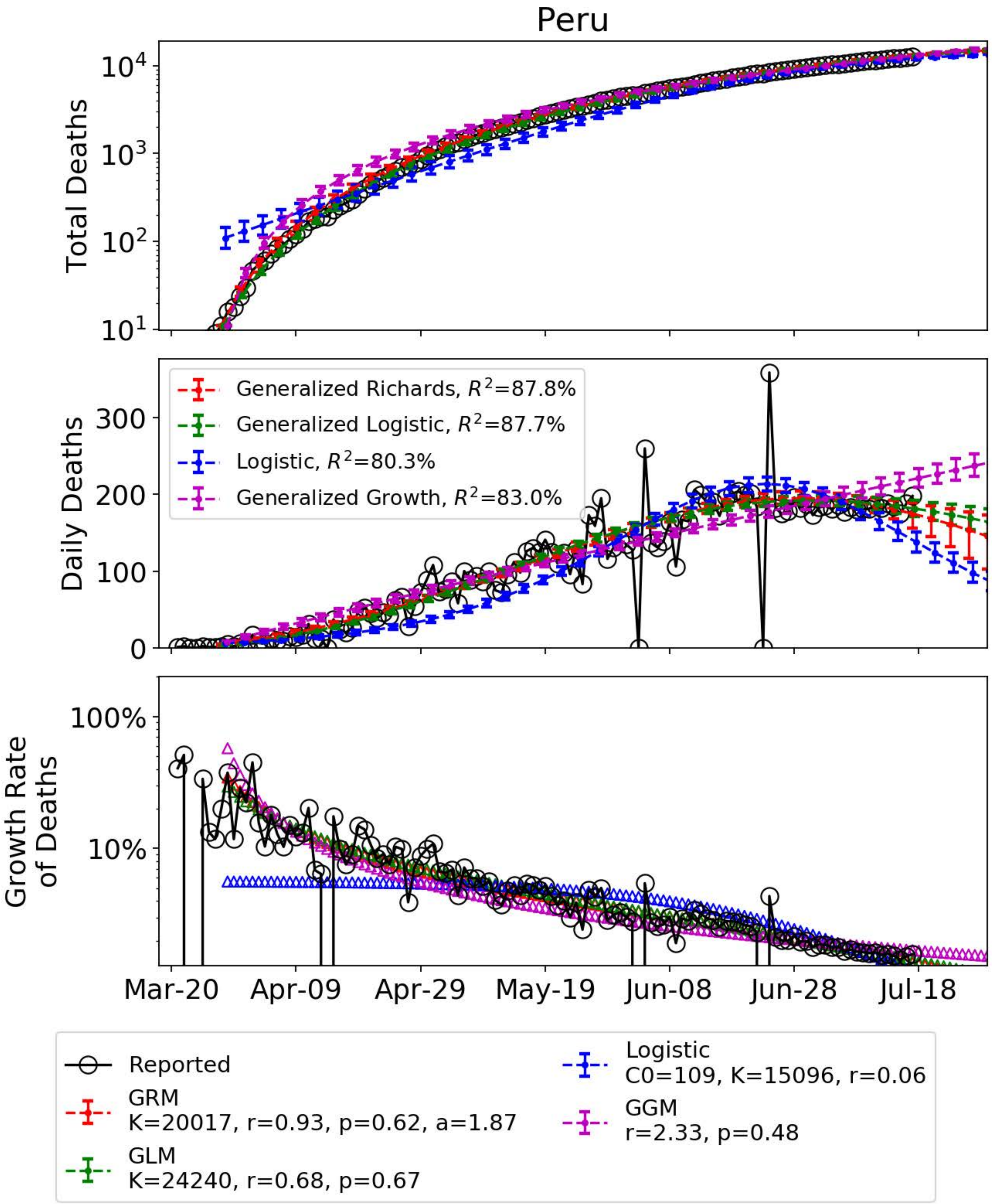

Peru
Total Deaths
Daily Deaths
Growth Rate of Deaths
Generalized Richards, R^2=87.8%
Generalized Logistic, R^2=87.7%
Logistic, R^2=80.3%
Generalized Growth, R^2=83.0%
Mar-20
Apr-09
Apr-29
May-19
Jun-08
Jun-28
Jul-18
Reported
GRM
K=20017, r=0.93, p=0.62, a=1.87
GLM
K=24240, r=0.68, p=0.67
Logistic
C0=109, K=15096, r=0.06
GGM
r=2.33, p=0.48

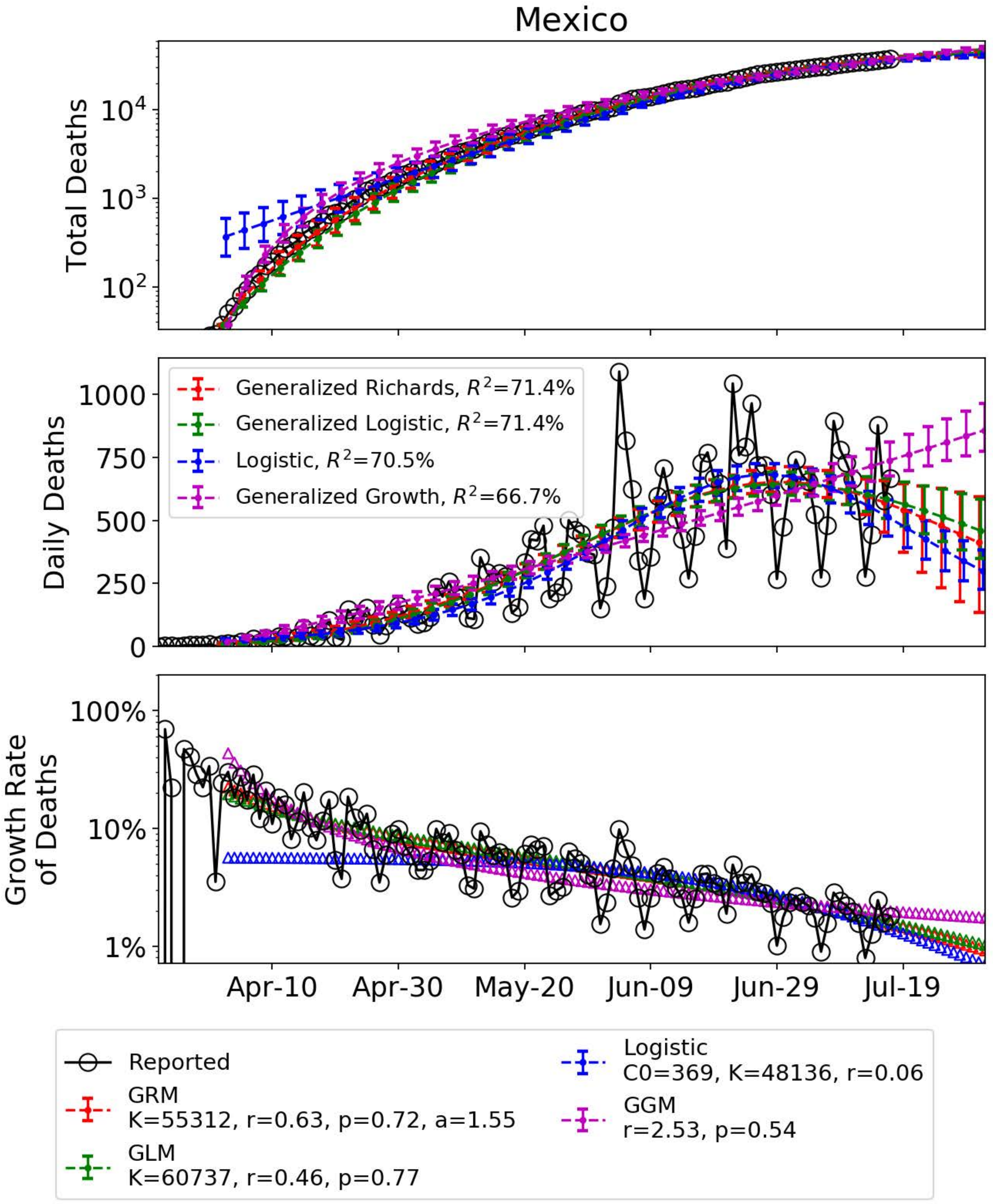
Mexico
Total Deaths
10^4
10^3
10^2
Daily Deaths
1000
750
500
250
0
Generalized Richards, R^2=71.4%
Generalized Logistic, R^2=71.4%
Logistic, R^2=70.5%
Generalized Growth, R^2=66.7%
Growth Rate of Deaths
100%
10%
1%
Apr-10
Apr-30
May-20
Jun-09
Jun-29
Jul-19
Reported
GRM
K=55312, r=0.63, p=0.72, a=1.55
GLM
K=60737, r=0.46, p=0.77
Logistic
C0=369, K=48136, r=0.06
GGM
r=2.53, p=0.54

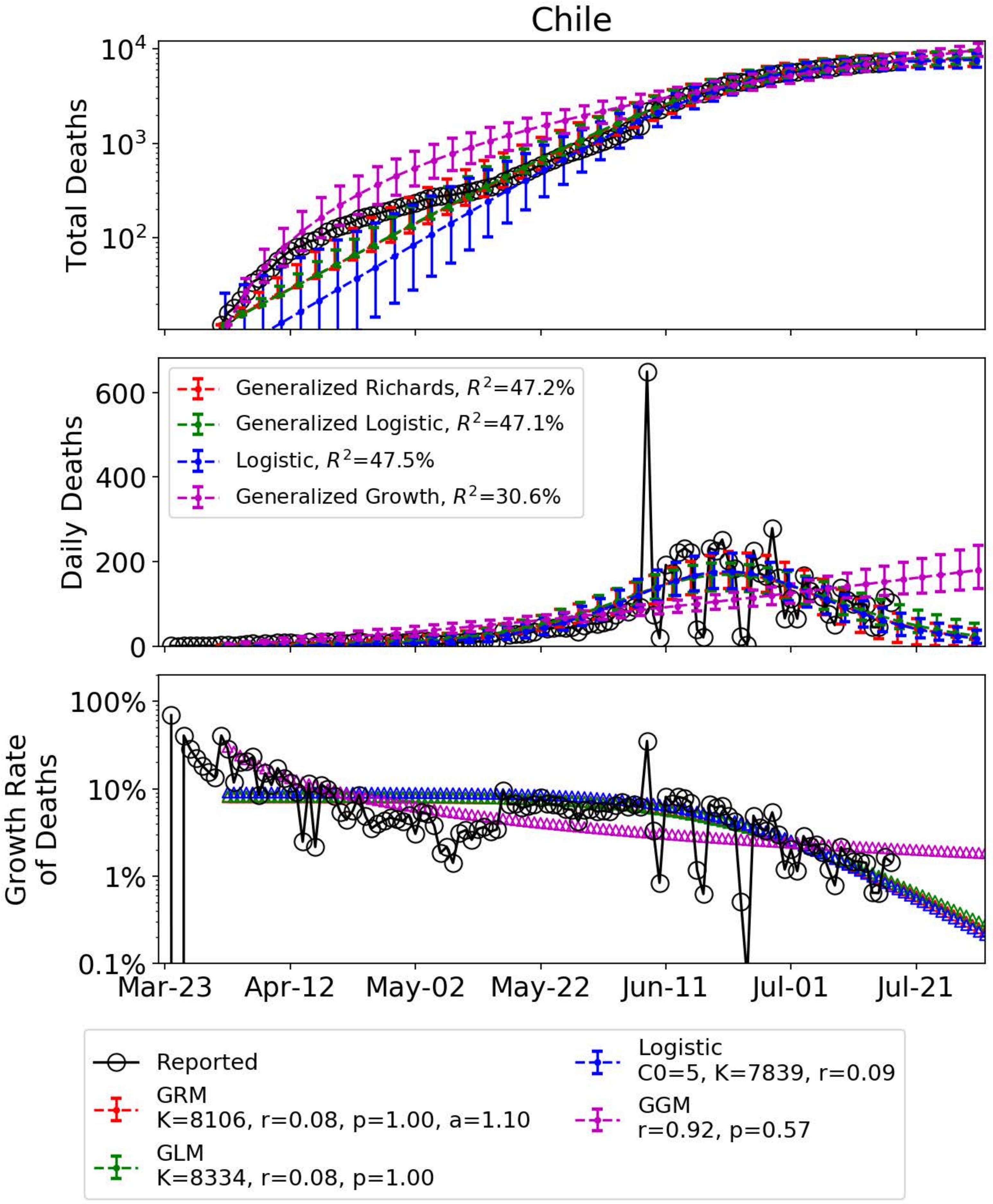

Chile
Total Deaths
Daily Deaths
Growth Rate of Deaths
Generalized Richards, R^2=47.2%
Generalized Logistic, R^2=47.1%
Logistic, R^2=47.5%
Generalized Growth, R^2=30.6%
Mar-23
Apr-12
May-02
May-22
Jun-11
Jul-01
Jul-21
Reported
GRM
K=8106, r=0.08, p=1.00, a=1.10
GLM
K=8334, r=0.08, p=1.00
Logistic
C0=5, K=7839, r=0.09
GGM
r=0.92, p=0.57

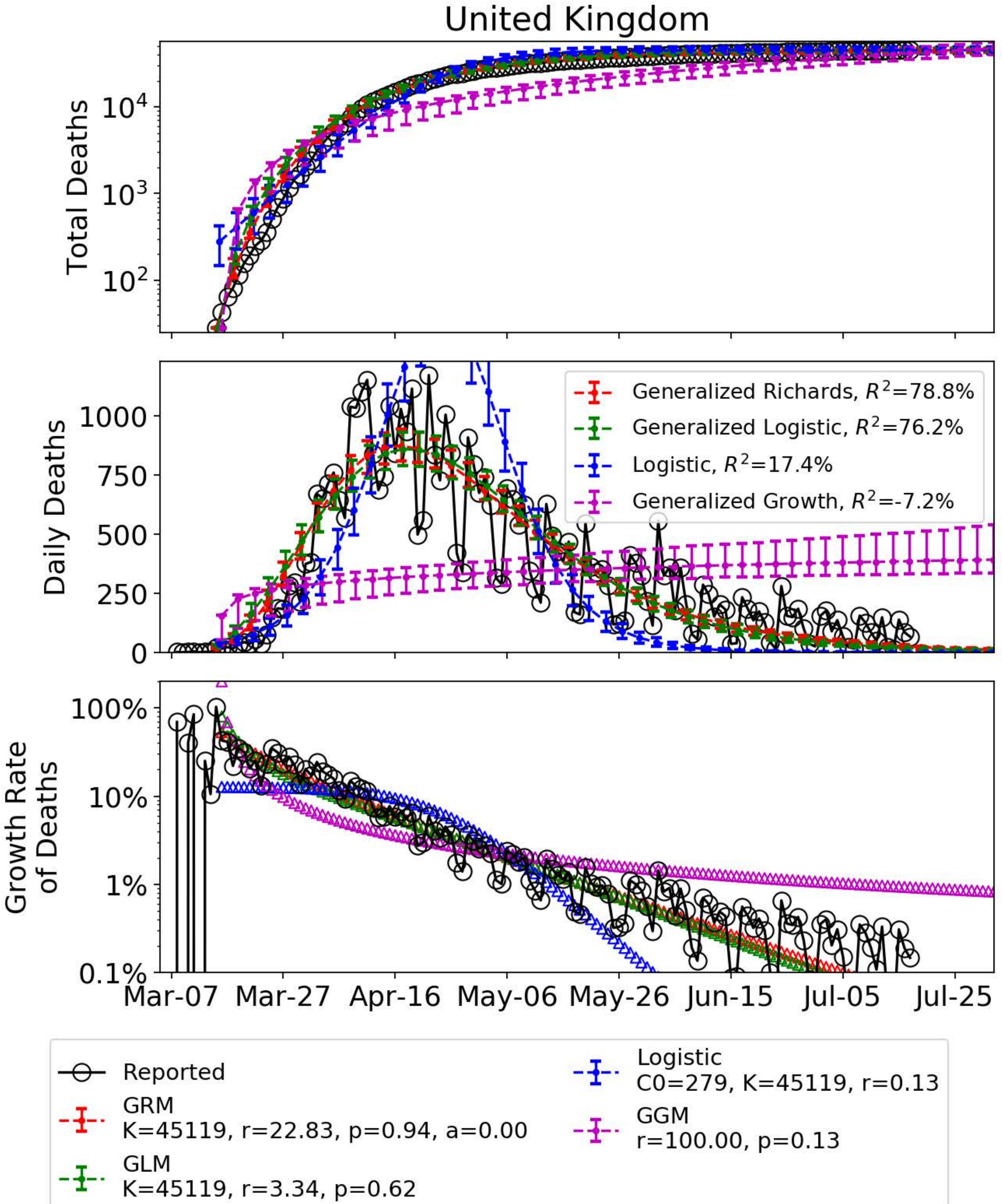

United Kingdom
Total Deaths
Daily Deaths
Growth Rate of Deaths
Generalized Richards, R^2=78.8%
Generalized Logistic, R^2=76.2%
Logistic, R^2=17.4%
Generalized Growth, R^2=-7.2%
Mar-07
Mar-27
Apr-16
May-06
May-26
Jun-15
Jul-05
Jul-25
Reported
GRM
K=45119, r=22.83, p=0.94, a=0.00
GLM
K=45119, r=3.34, p=0.62
Logistic
C0=279, K=45119, r=0.13
GGM
r=100.00, p=0.13

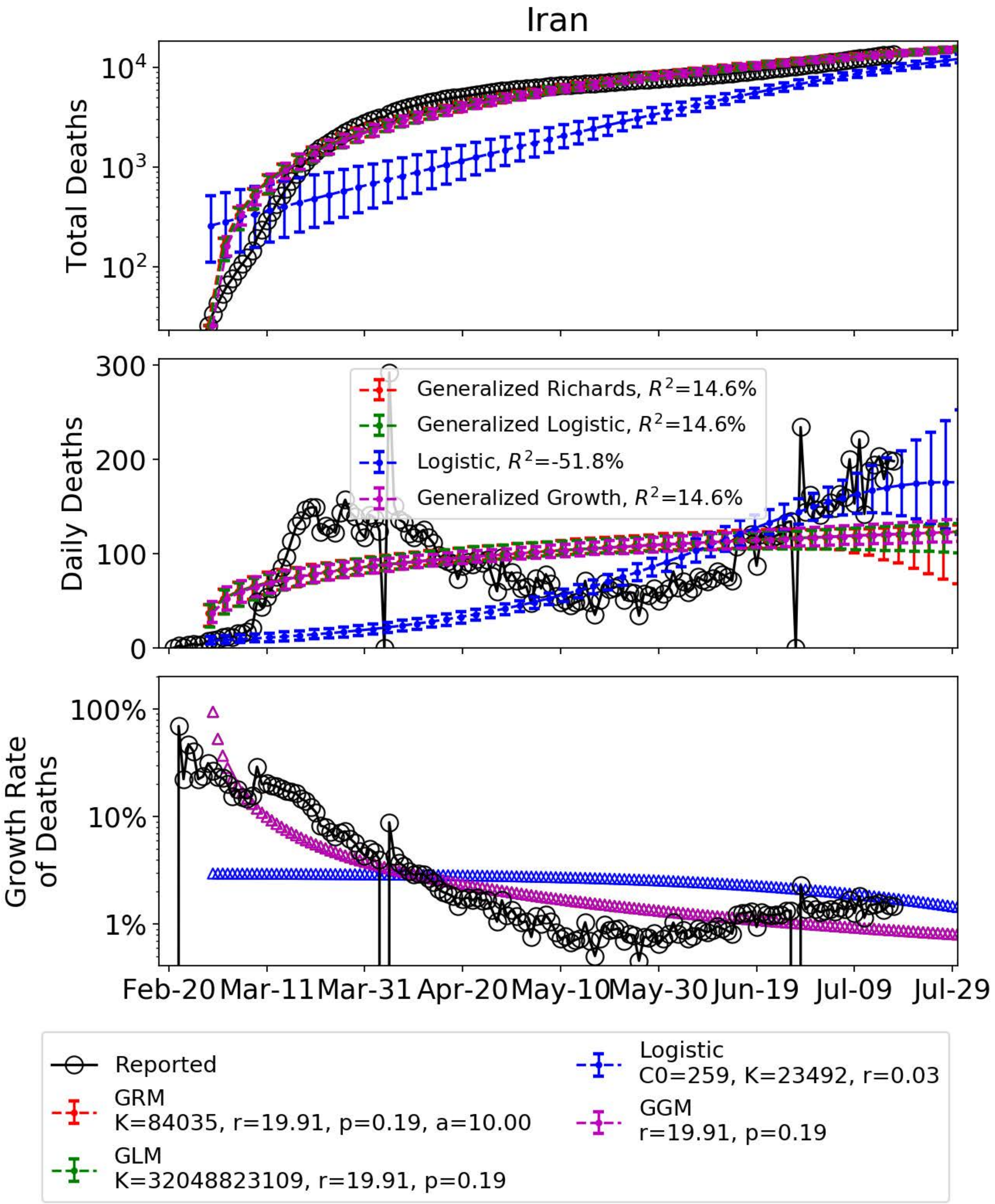

Iran
Total Deaths
Daily Deaths
Growth Rate of Deaths
Generalized Richards, R^2=14.6%
Generalized Logistic, R^2=14.6%
Logistic, R^2=-51.8%
Generalized Growth, R^2=14.6%
Feb-20
Mar-11
Mar-31
Apr-20
May-10
May-30
Jun-19
Jul-09
Jul-29
Reported
GRM
K=84035, r=19.91, p=0.19, a=10.00
GLM
K=32048823109, r=19.91, p=0.19
Logistic
C0=259, K=23492, r=0.03
GGM
r=19.91, p=0.19

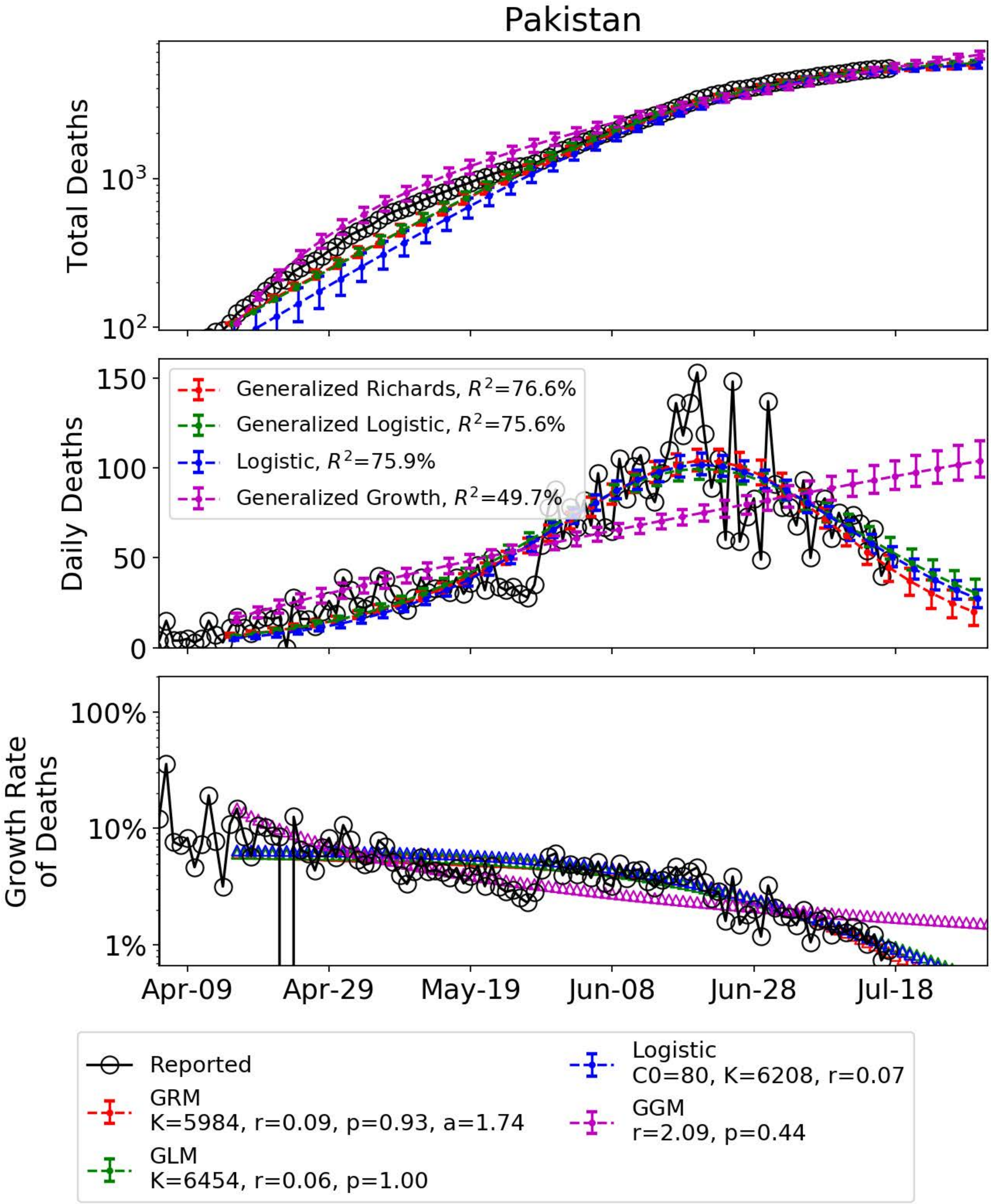

Pakistan
Total Deaths
10^3
10^2
Daily Deaths
150
100
50
0
Generalized Richards, R^2=76.6%
Generalized Logistic, R^2=75.6%
Logistic, R^2=75.9%
Generalized Growth, R^2=49.7%
Growth Rate of Deaths
100%
10%
1%
Apr-09
Apr-29
May-19
Jun-08
Jun-28
Jul-18
Reported
GRM
K=5984, r=0.09, p=0.93, a=1.74
GLM
K=6454, r=0.06, p=1.00
Logistic
C0=80, K=6208, r=0.07
GGM
r=2.09, p=0.44

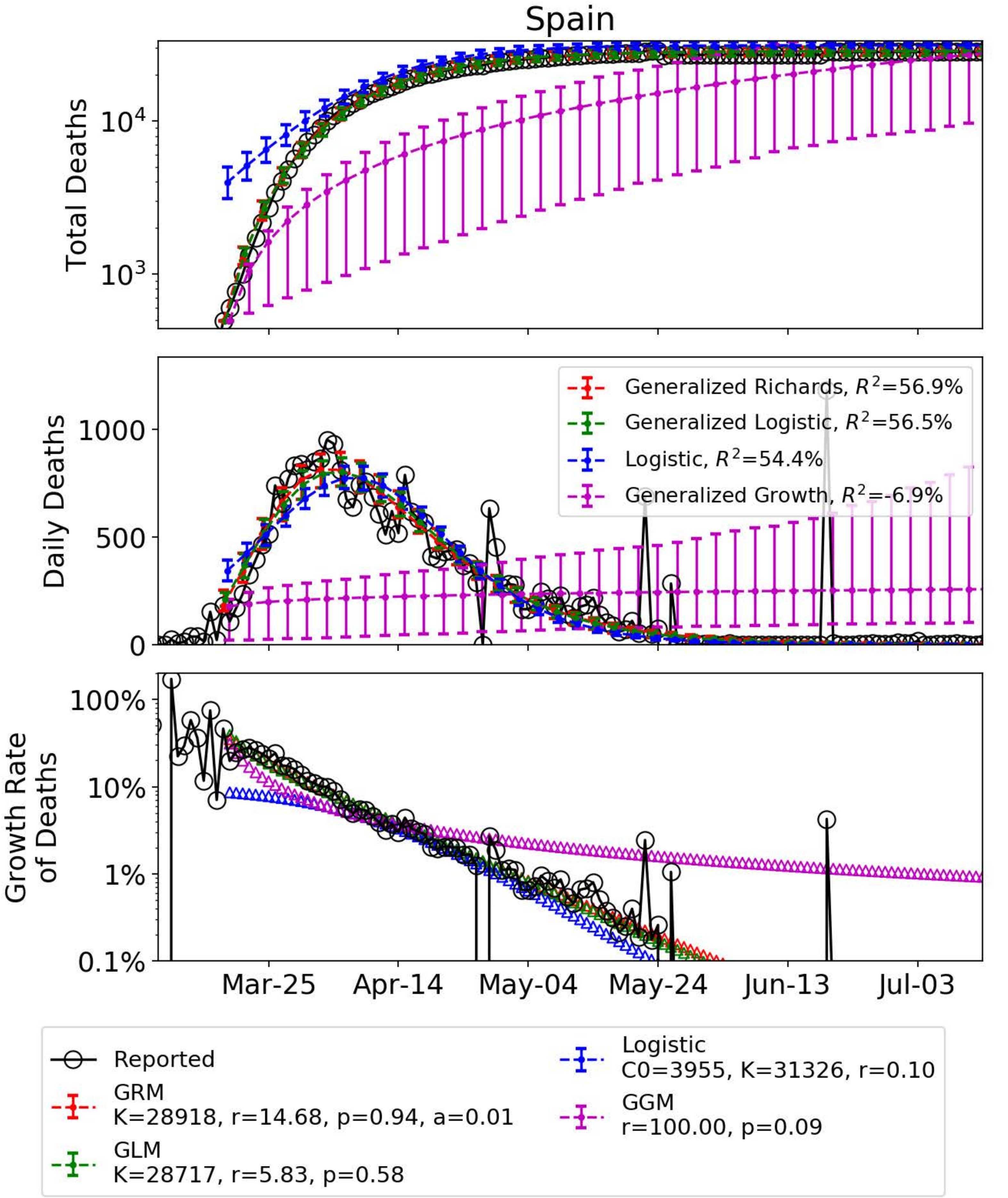

Spain
Total Deths
Total Deaths
10^4
10^3
Daily Deaths
1000
500
0
Generalized Richards, R^2=56.9%
Generalized Logistic, R^2=56.5%
Logistic, R^2=54.4%
Generalized Growth, R^2=-6.9%
Growth Rate of Deaths
100%
10%
1%
0.1%
Mar-25
Apr-14
May-04
May-24
Jun-13
Jul-03
Reported
GRM
K=28918, r=14.68, p=0.94, a=0.01
GLM
K=28717, r=5.83, p=0.58
Logistic
C0=3955, K=31326, r=0.10
GGM
r=100.00, p=0.09

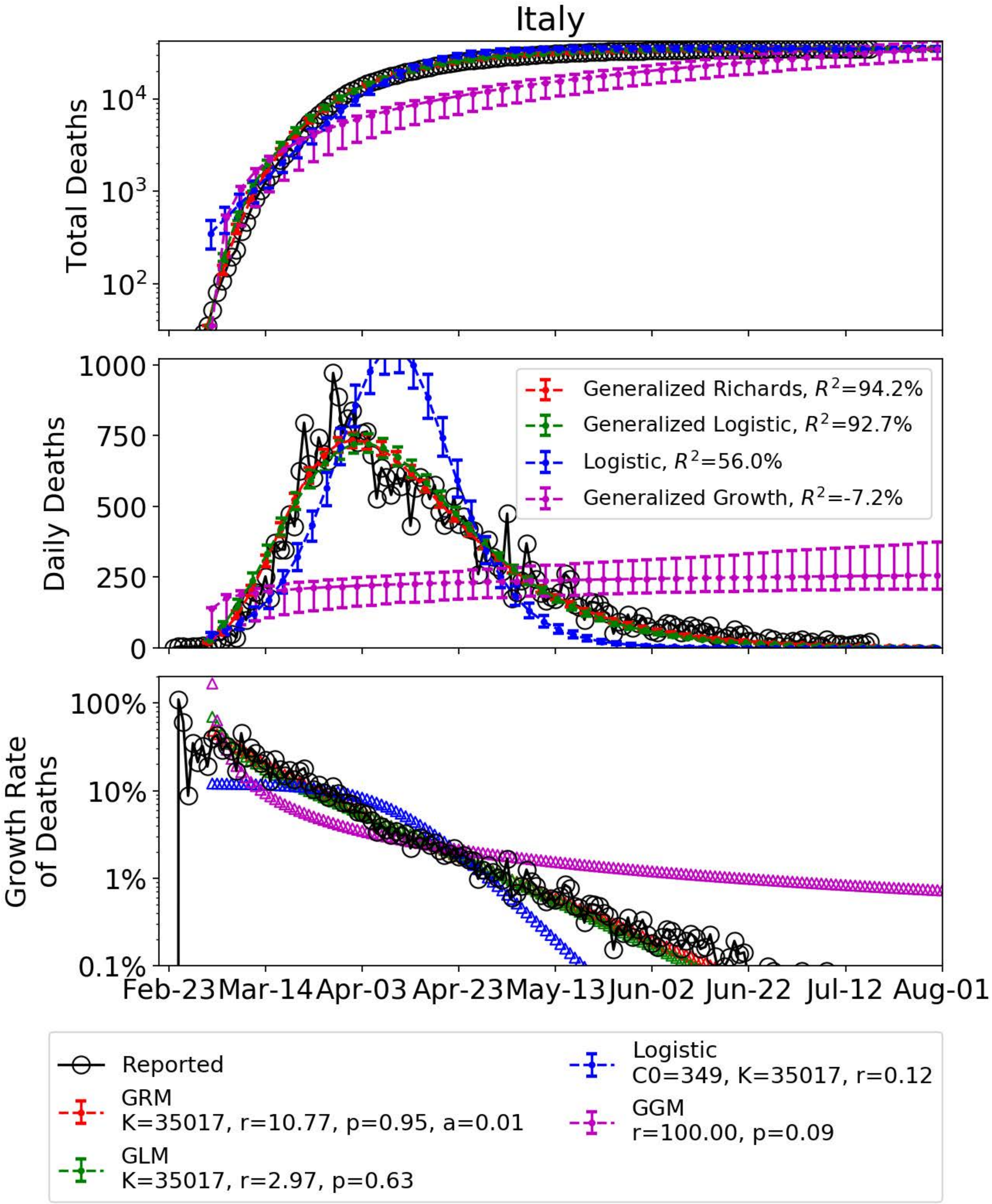
Italy
Total Deaths
10^4
10^3
10^2
Daily Deaths
1000
750
500
250
0
Generalized Richards, R^2=94.2%
Generalized Logistic, R^2=92.7%
Logistic, R^2=56.0%
Generalized Growth, R^2=-7.2%
Growth Rate of Deaths
100%
10%
1%
0.1%
Feb-23
Mar-14
Apr-03
Apr-23
May-13
Jun-02
Jun-22
Jul-12
Aug-01
Reported
GRM
K=35017, r=10.77, p=0.95, a=0.01
GLM
K=35017, r=2.97, p=0.63
Logistic
C0=349, K=35017, r=0.12
GGM
r=100.00, p=0.09

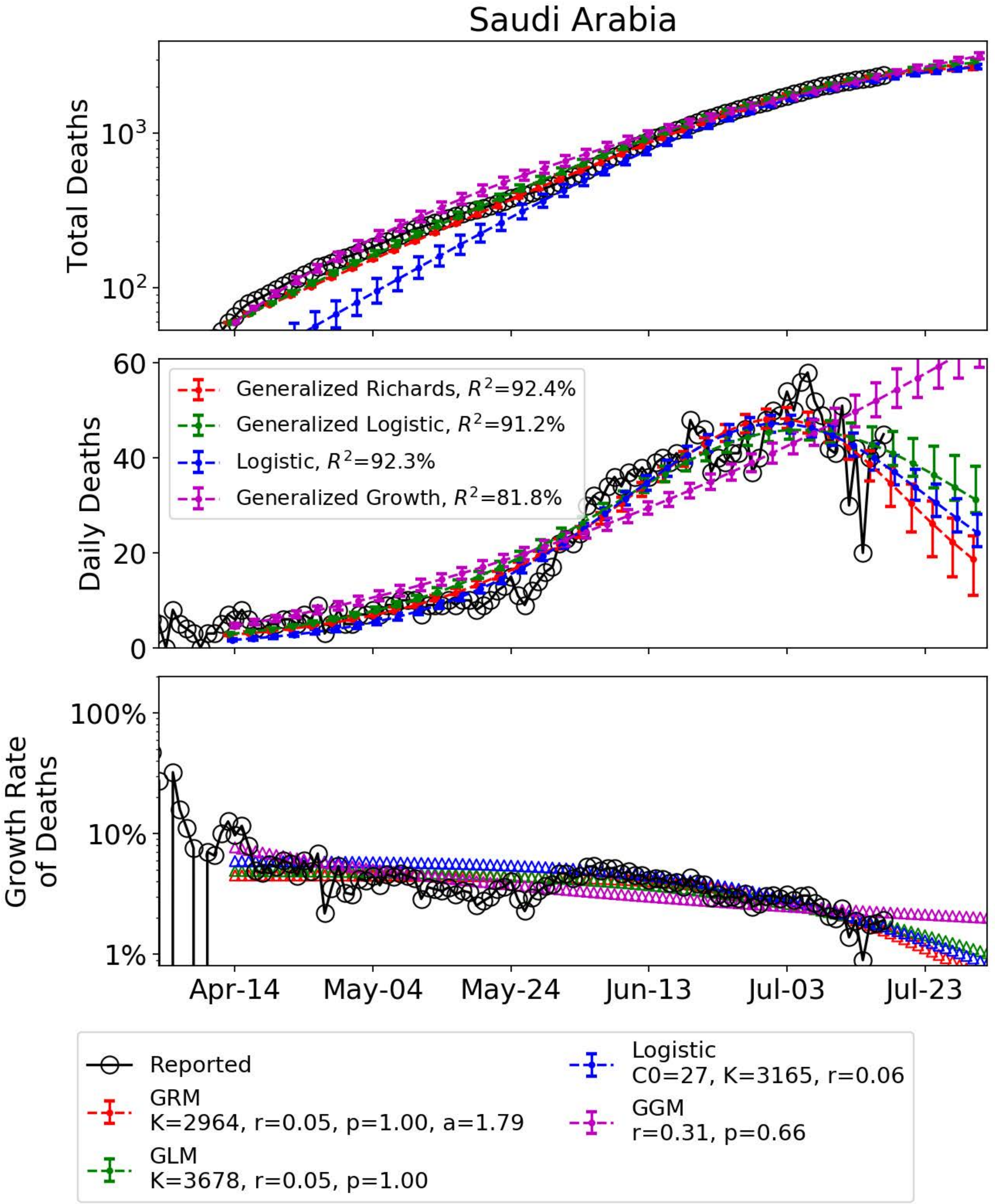

Saudi Arabia
Total Deths
10^3
10^2
Daily Deaths
Generalized Richards, R^2=92.4%
Generalized Logistic, R^2=91.2%
Logistic, R^2=92.3%
Generalized Growth, R^2=81.8%
60
40
20
0
Growth Rate of Deaths
100%
10%
1%
Apr-14
May-04
May-24
Jun-13
Jul-03
Jul-23
Reported
GRM
K=2964, r=0.05, p=1.00, a=1.79
GLM
K=3678, r=0.05, p=1.00
Logistic
C0=27, K=3165, r=0.06
GGM
r=0.31, p=0.66

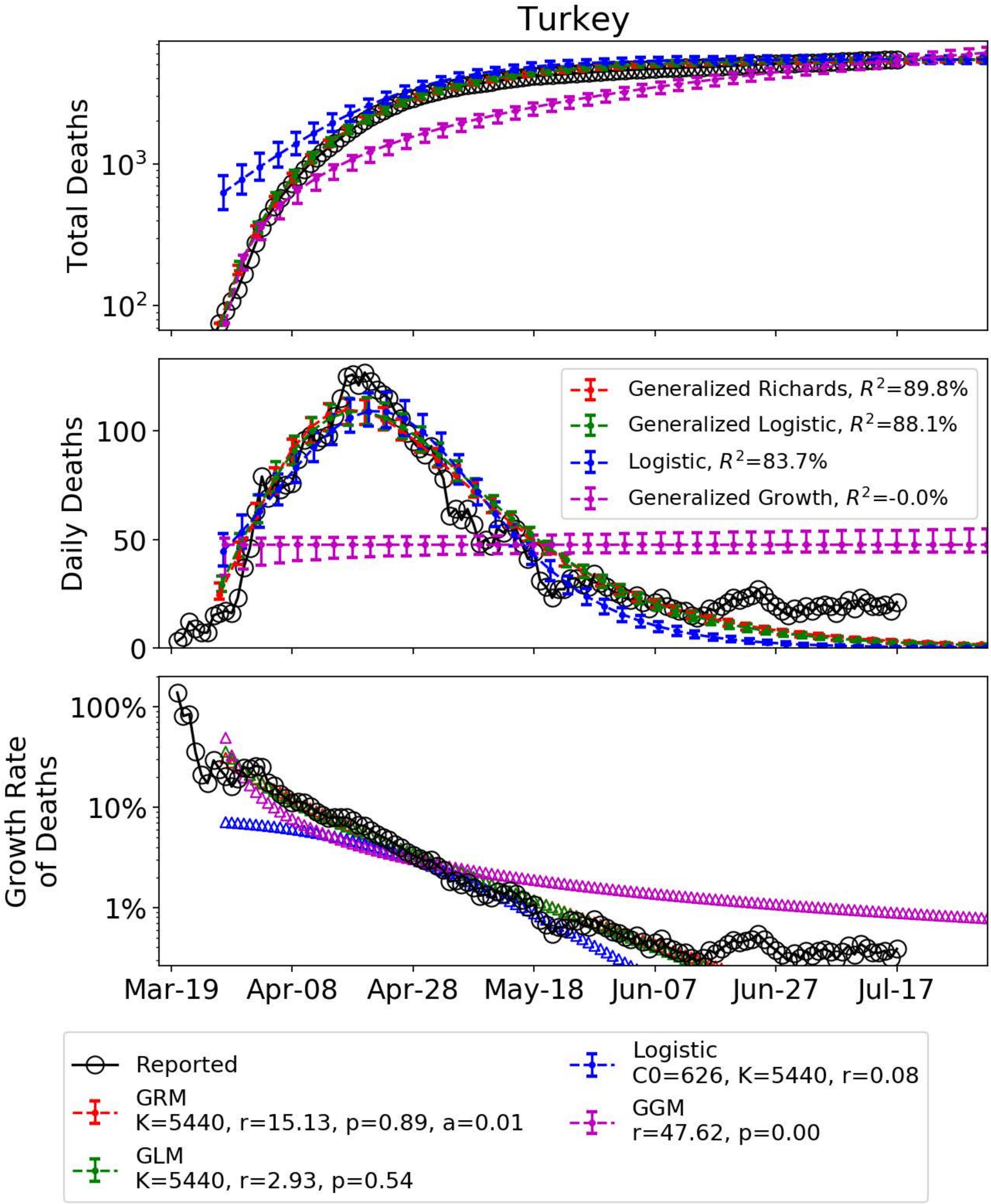
Turkey
Total Deaths
10^3
10^2
Daily Deaths
100
50
0
Generalized Richards, R^2=89.8%
Generalized Logistic, R^2=88.1%
Logistic, R^2=83.7%
Generalized Growth, R^2=-0.0%
Growth Rate of Deaths
100%
10%
1%
Mar-19
Apr-08
Apr-28
May-18
Jun-07
Jun-27
Jul-17
Reported
GRM
K=5440, r=15.13, p=0.89, a=0.01
GLM
K=5440, r=2.93, p=0.54
Logistic
C0=626, K=5440, r=0.08
GGM
r=47.62, p=0.00

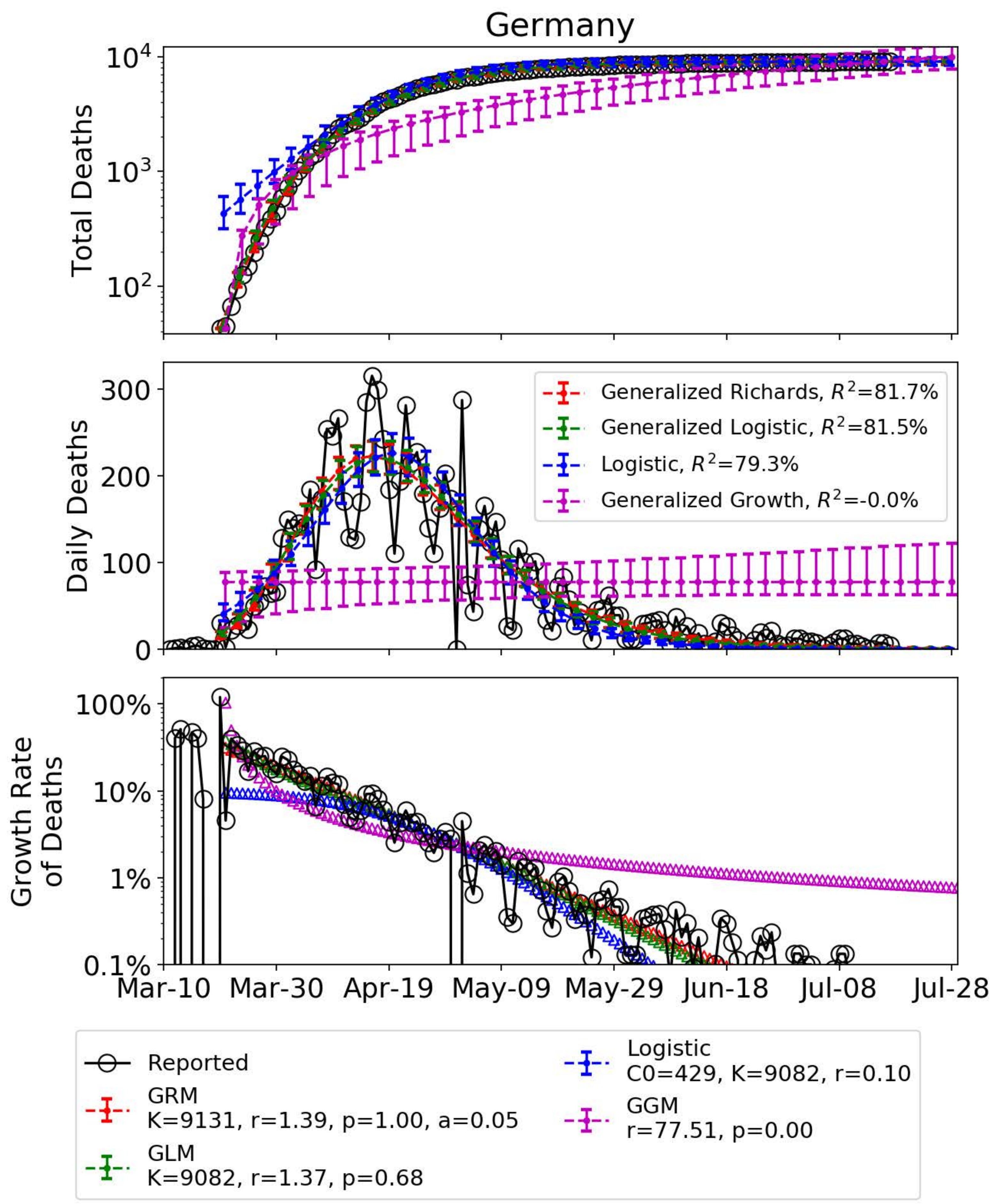

Germany
Total Deaths
Daily Deaths
Growth Rate of Deaths
Generalized Richards, R^2=81.7%
Generalized Logistic, R^2=81.5%
Logistic, R^2=79.3%
Generalized Growth, R^2=-0.0%
Mar-10
Mar-30
Apr-19
May-09
May-29
Jun-18
Jul-08
Jul-28
Reported
GRM
K=9131, r=1.39, p=1.00, a=0.05
GLM
K=9082, r=1.37, p=0.68
Logistic
C0=429, K=9082, r=0.10
GGM
r=77.51, p=0.00

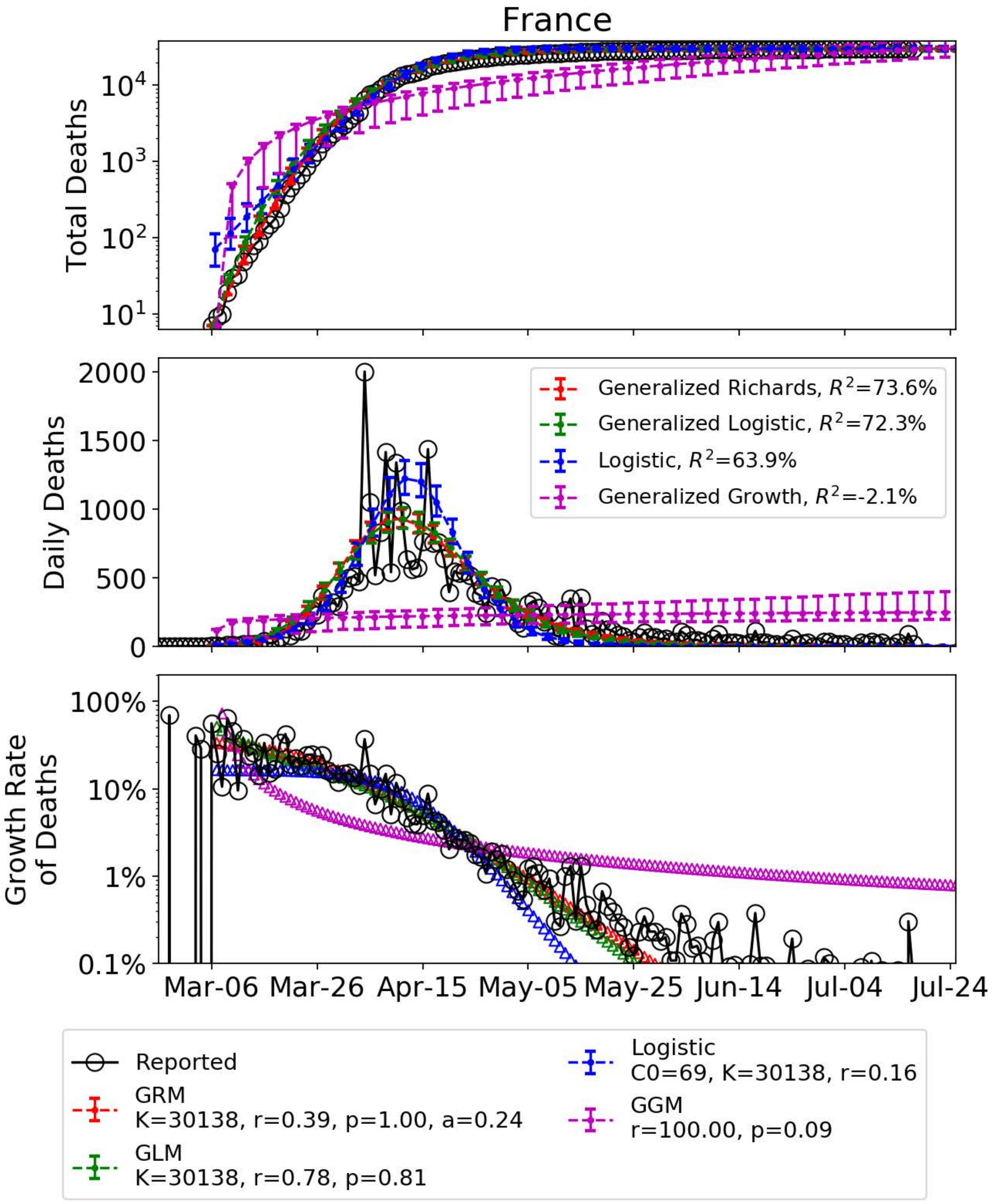
France
Total Deaths
Daily Deaths
Growth Rate of Deaths
Generalized Richards, R^2=73.6%
Generalized Logistic, R^2=72.3%
Logistic, R^2=63.9%
Generalized Growth, R^2=-2.1%
Mar-06
Mar-26
Apr-15
May-05
May-25
Jun-14
Jul-04
Jul-24
Reported
GRM
K=30138, r=0.39, p=1.00, a=0.24
GLM
K=30138, r=0.78, p=0.81
Logistic
C0=69, K=30138, r=0.16
GGM
r=100.00, p=0.09

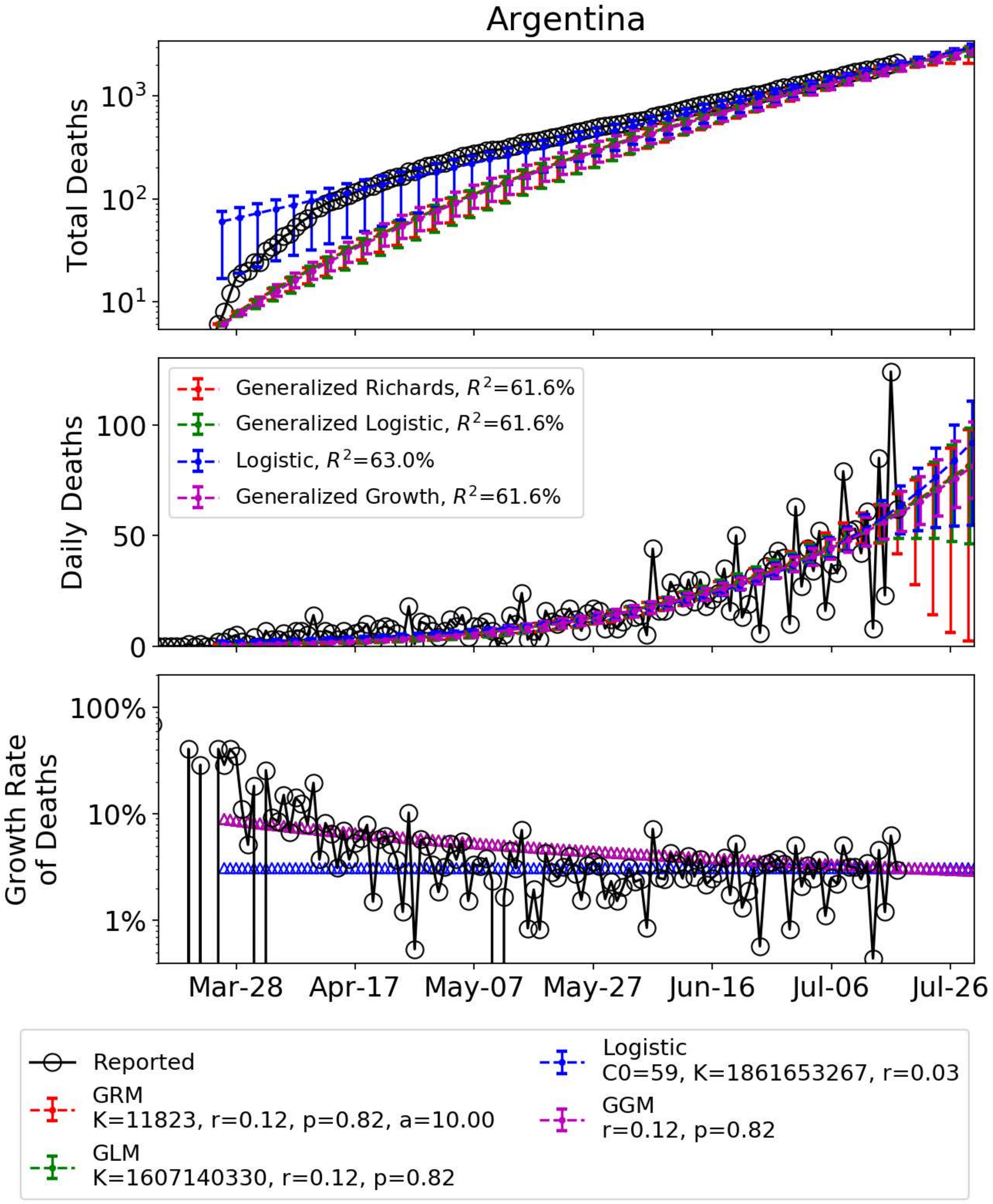

Argentina
Total Deaths
Daily Deaths
Generalized Richards, R^2=61.6%
Generalized Logistic, R^2=61.6%
Logistic, R^2=63.0%
Generalized Growth, R^2=61.6%
Growth Rate of Deaths
Mar-28
Apr-17
May-07
May-27
Jun-16
Jul-06
Jul-26
Reported
GRM
K=11823, r=0.12, p=0.82, a=10.00
GLM
K=1607140330, r=0.12, p=0.82
Logistic
C0=59, K=1861653267, r=0.03
GGM
r=0.12, p=0.82

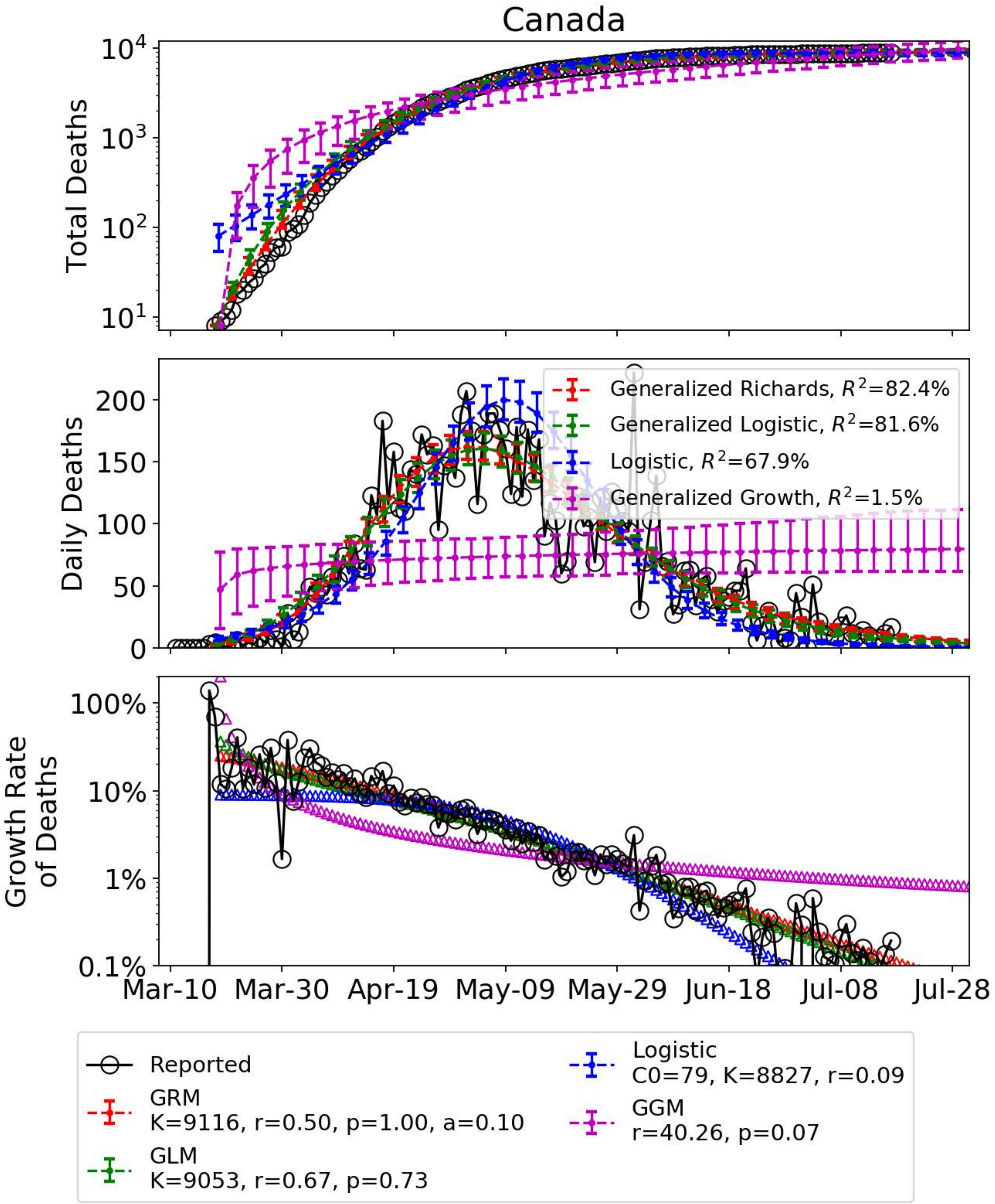
Canada
Total Deaths
Daily Deaths
Growth Rate of Deaths
Generalized Richards, R^2=82.4%
Generalized Logistic, R^2=81.6%
Logistic, R^2=67.9%
Generalized Growth, R^2=1.5%
Mar-10
Mar-30
Apr-19
May-09
May-29
Jun-18
Jul-08
Jul-28
Reported
GRM
K=9116, r=0.50, p=1.00, a=0.10
GLM
K=9053, r=0.67, p=0.73
Logistic
C0=79, K=8827, r=0.09
GGM
r=40.26, p=0.07

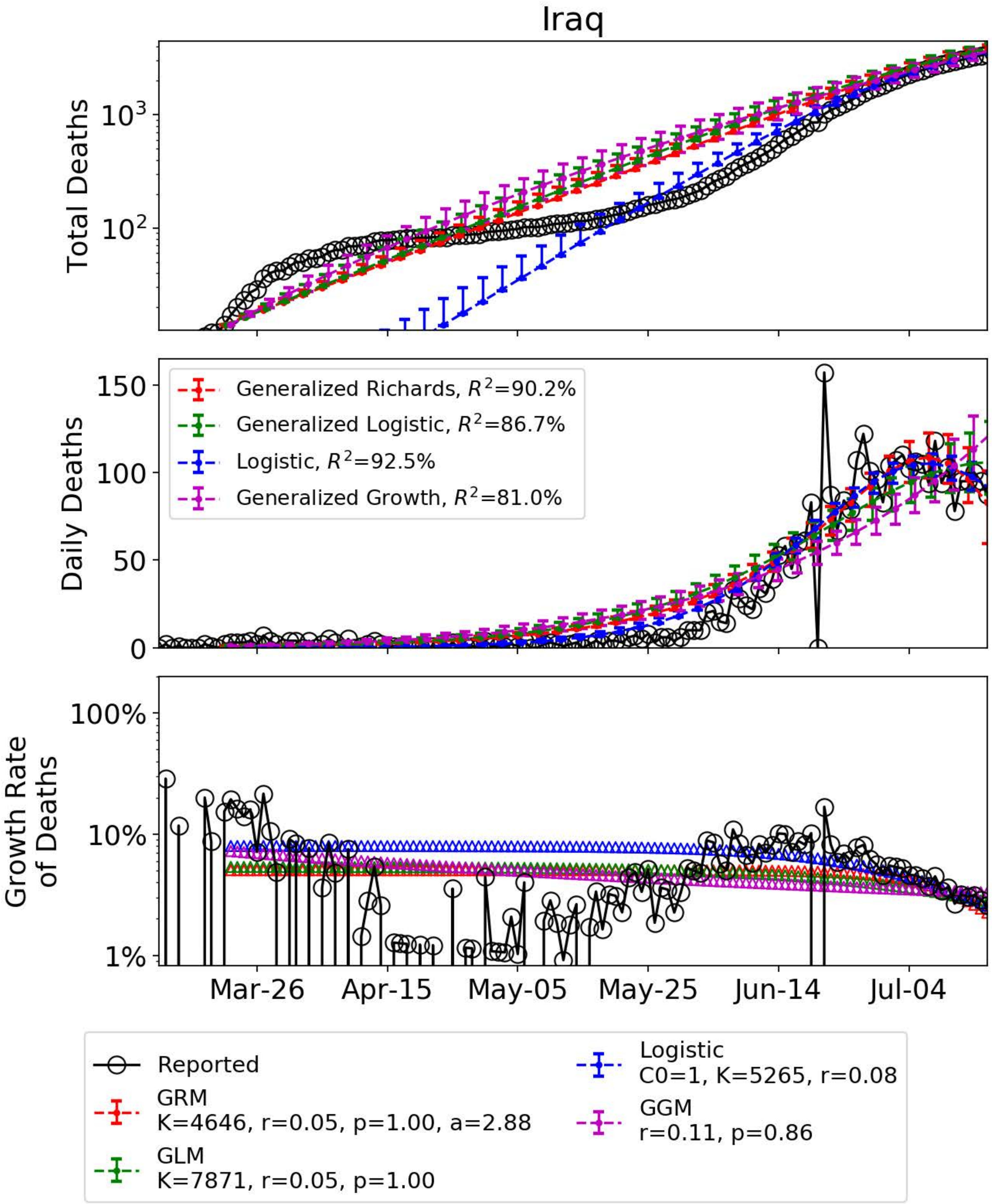

Iraq
Total Deaths
10^3
10^2
Daily Deaths
150
100
50
0
Generalized Richards, R^2=90.2%
Generalized Logistic, R^2=86.7%
Logistic, R^2=92.5%
Generalized Growth, R^2=81.0%
Growth Rate of Deaths
100%
10%
1%
Mar-26
Apr-15
May-05
May-25
Jun-14
Jul-04
Reported
GRM
K=4646, r=0.05, p=1.00, a=2.88
GLM
K=7871, r=0.05, p=1.00
Logistic
C0=1, K=5265, r=0.08
GGM
r=0.11, p=0.86

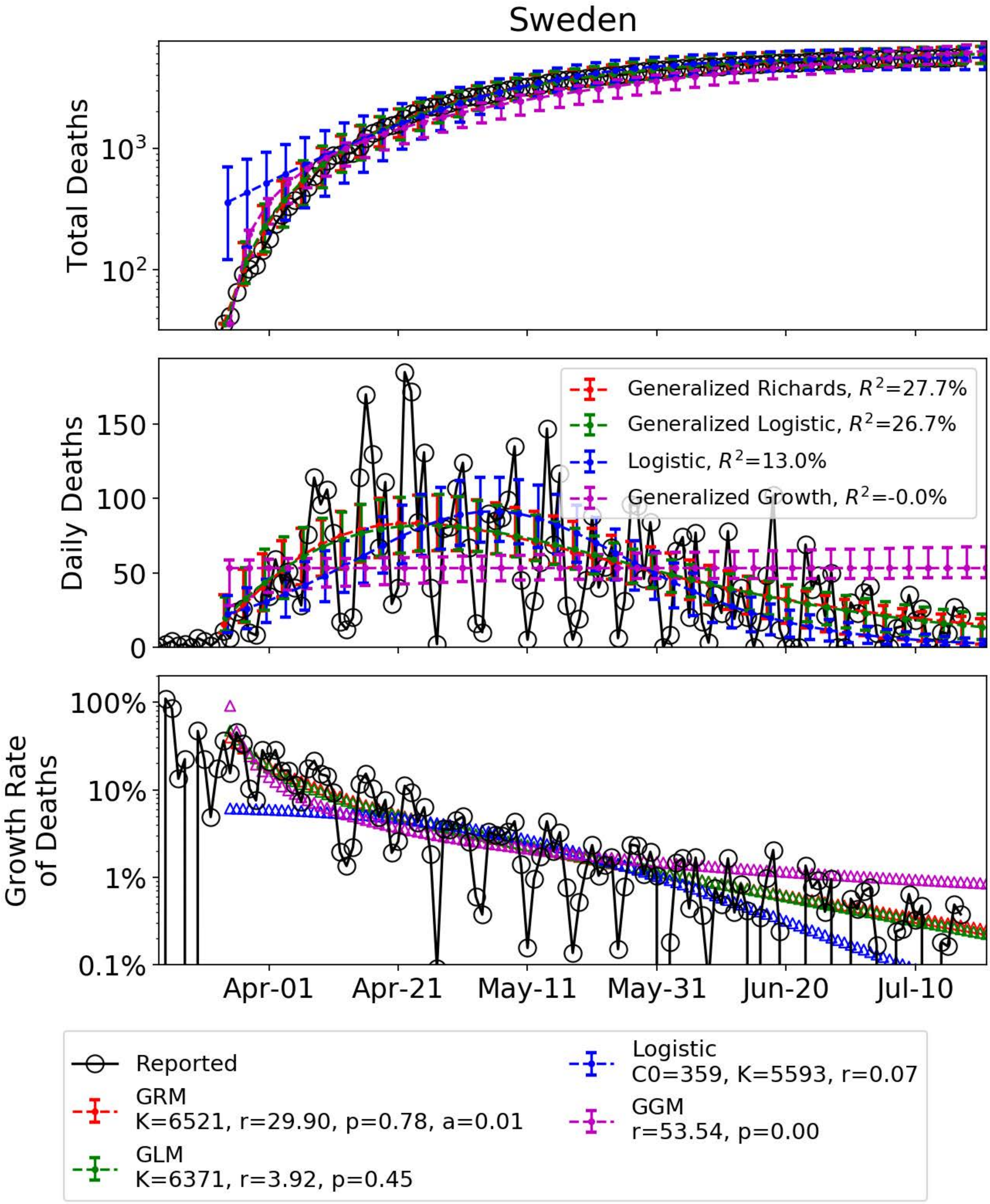

Sweden
Total Deths
Daily Deaths
Growth Rate of Deaths
Generalized Richards, R^2=27.7%
Generalized Logistic, R^2=26.7%
Logistic, R^2=13.0%
Generalized Growth, R^2=-0.0%
Apr-01
Apr-21
May-11
May-31
Jun-20
Jul-10
Reported
GRM
K=6521, r=29.90, p=0.78, a=0.01
GLM
K=6371, r=3.92, p=0.45
Logistic
C0=359, K=5593, r=0.07
GGM
r=53.54, p=0.00

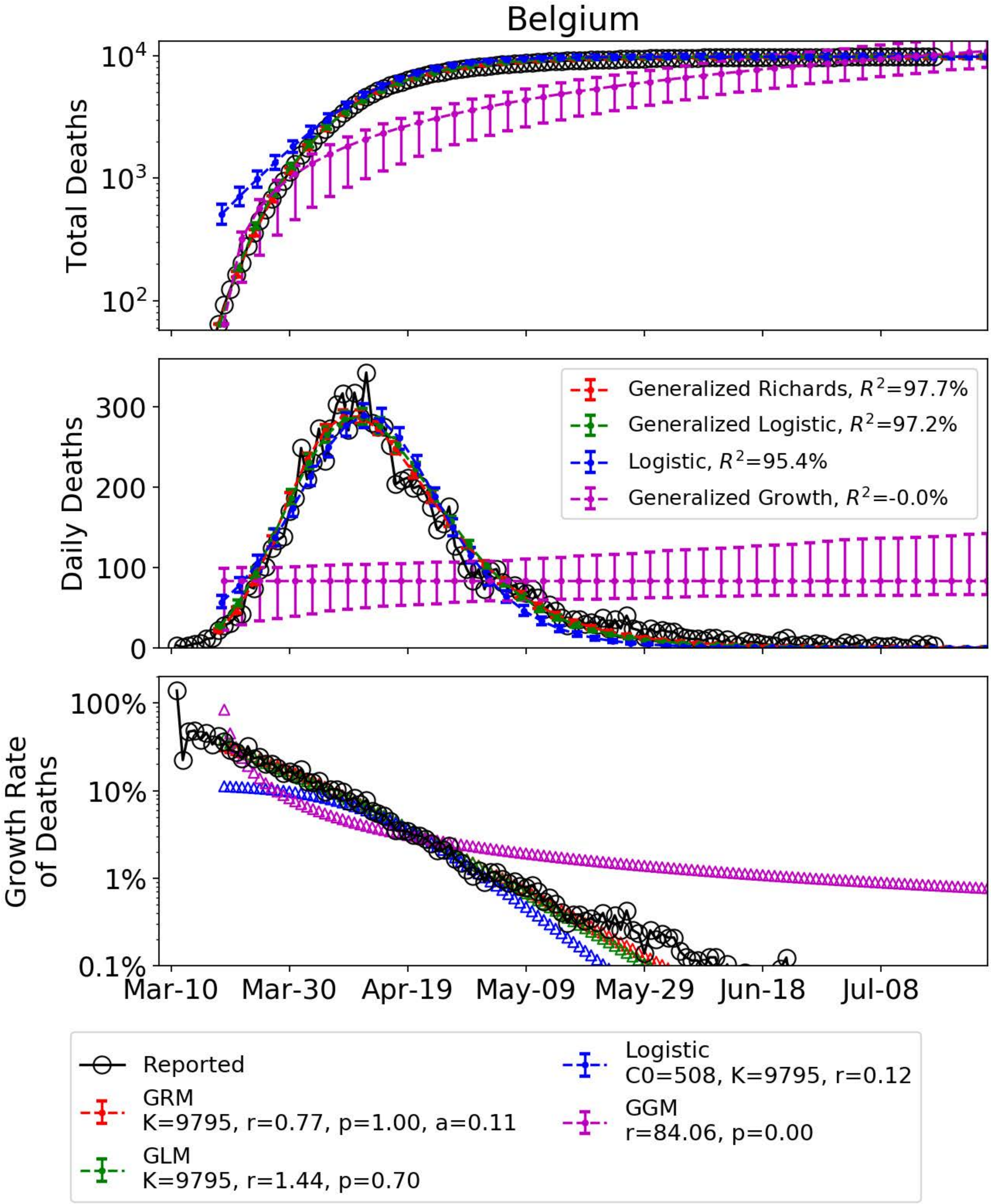

Belgium
Total Deaths
10^4
10^3
10^2
Daily Deaths
300
200
100
0
Generalized Richards, R^2=97.7%
Generalized Logistic, R^2=97.2%
Logistic, R^2=95.4%
Generalized Growth, R^2=-0.0%
Growth Rate of Deaths
100%
10%
1%
0.1%
Mar-10
Mar-30
Apr-19
May-09
May-29
Jun-18
Jul-08
Reported
GRM
K=9795, r=0.77, p=1.00, a=0.11
GLM
K=9795, r=1.44, p=0.70
Logistic
C0=508, K=9795, r=0.12
GGM
r=84.06, p=0.00

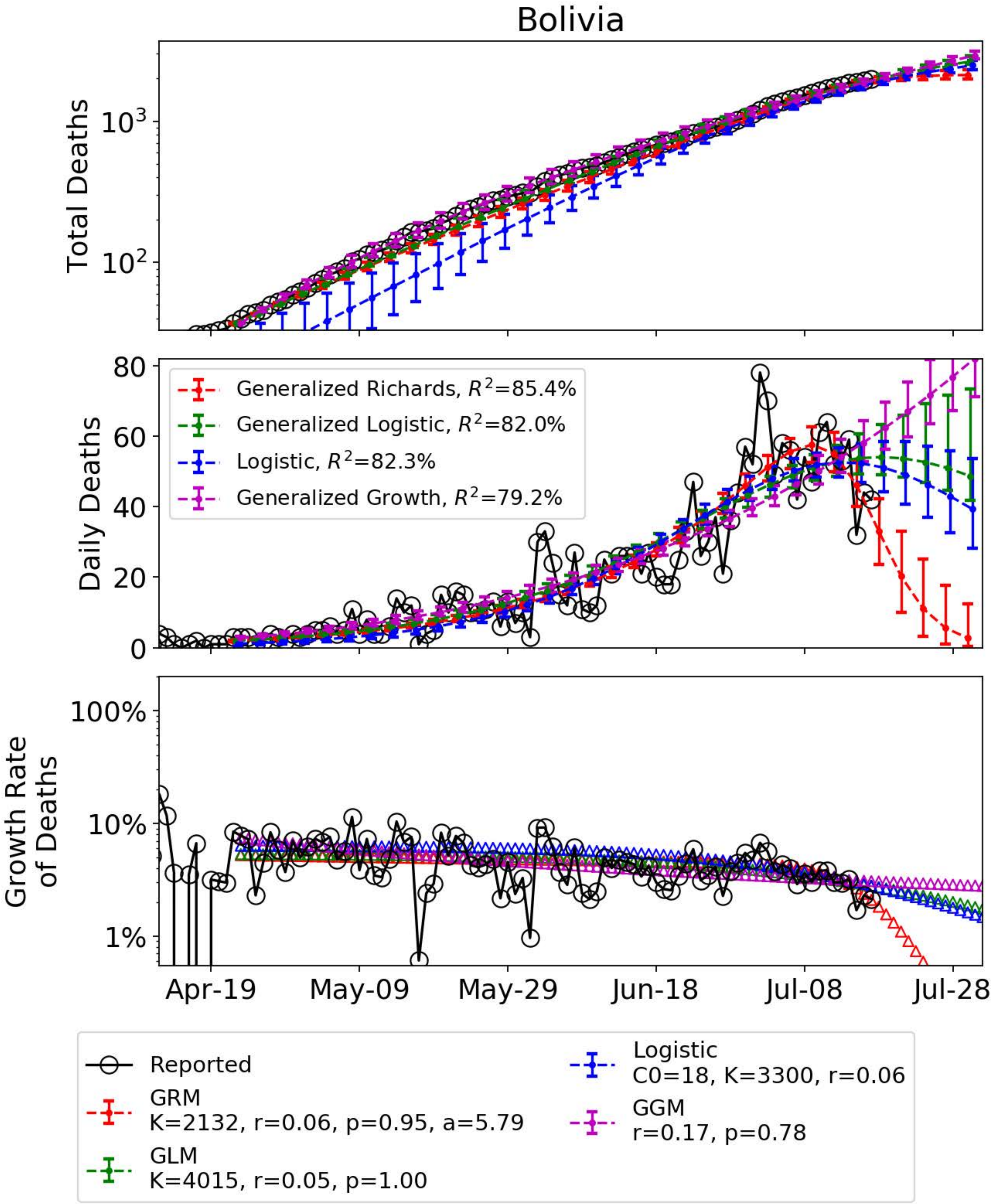
Bolivia
Total Deaths
10^3
10^2
Daily Deaths
80
60
40
20
0
Generalized Richards, R^2=85.4%
Generalized Logistic, R^2=82.0%
Logistic, R^2=82.3%
Generalized Growth, R^2=79.2%
Growth Rate of Deaths
100%
10%
1%
Apr-19
May-09
May-29
Jun-18
Jul-08
Jul-28
Reported
GRM
K=2132, r=0.06, p=0.95, a=5.79
GLM
K=4015, r=0.05, p=1.00
Logistic
C0=18, K=3300, r=0.06
GGM
r=0.17, p=0.78

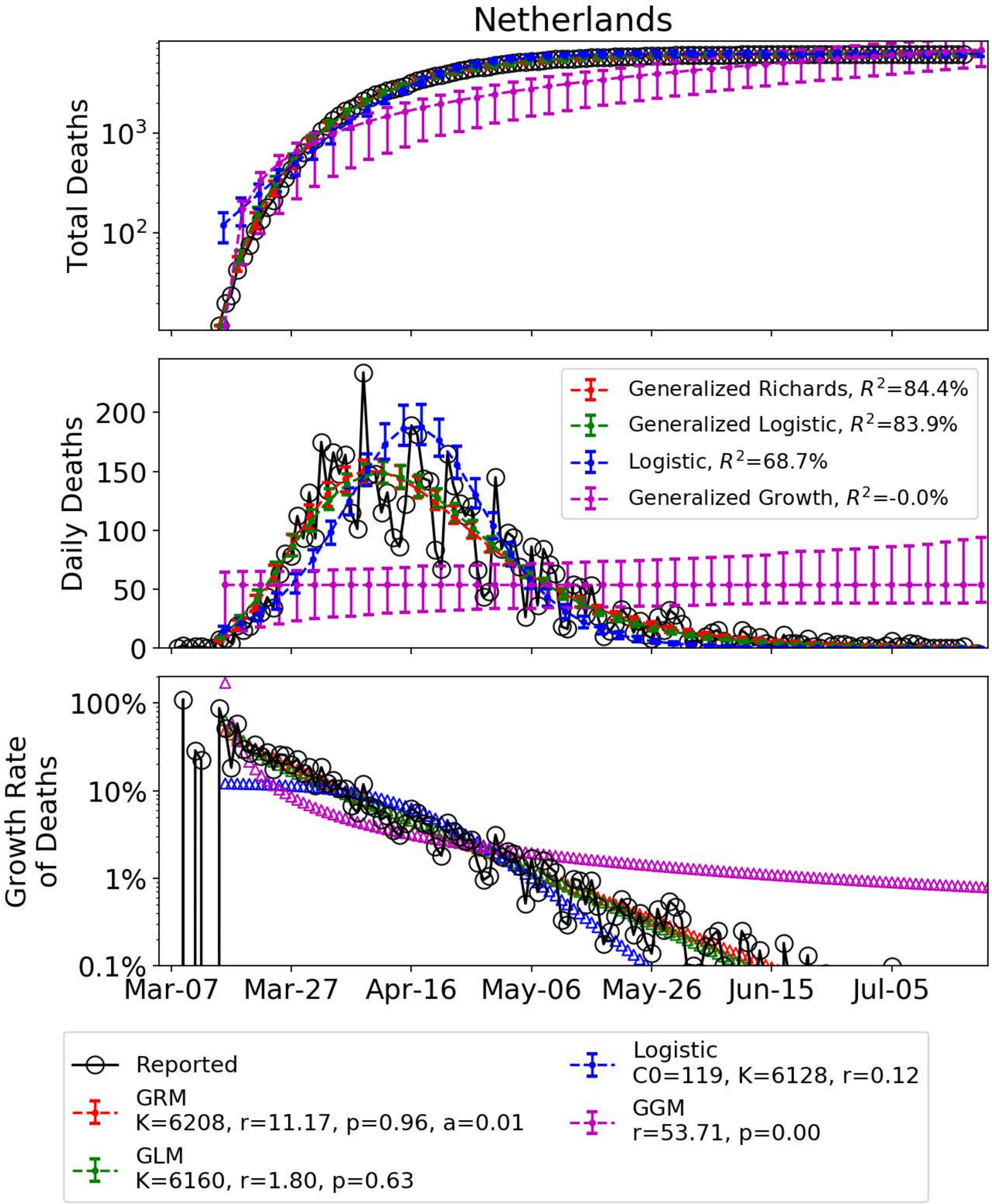

Netherlands
Total Deths
Daily Deaths
Growth Rate of Deaths
Generalized Richards, R^2=84.4%
Generalized Logistic, R^2=83.9%
Logistic, R^2=68.7%
Generalized Growth, R^2=-0.0%
Mar-07
Mar-27
Apr-16
May-06
May-26
Jun-15
Jul-05
Reported
GRM
K=6208, r=11.17, p=0.96, a=0.01
GLM
K=6160, r=1.80, p=0.63
Logistic
C0=119, K=6128, r=0.12
GGM
r=53.71, p=0.00

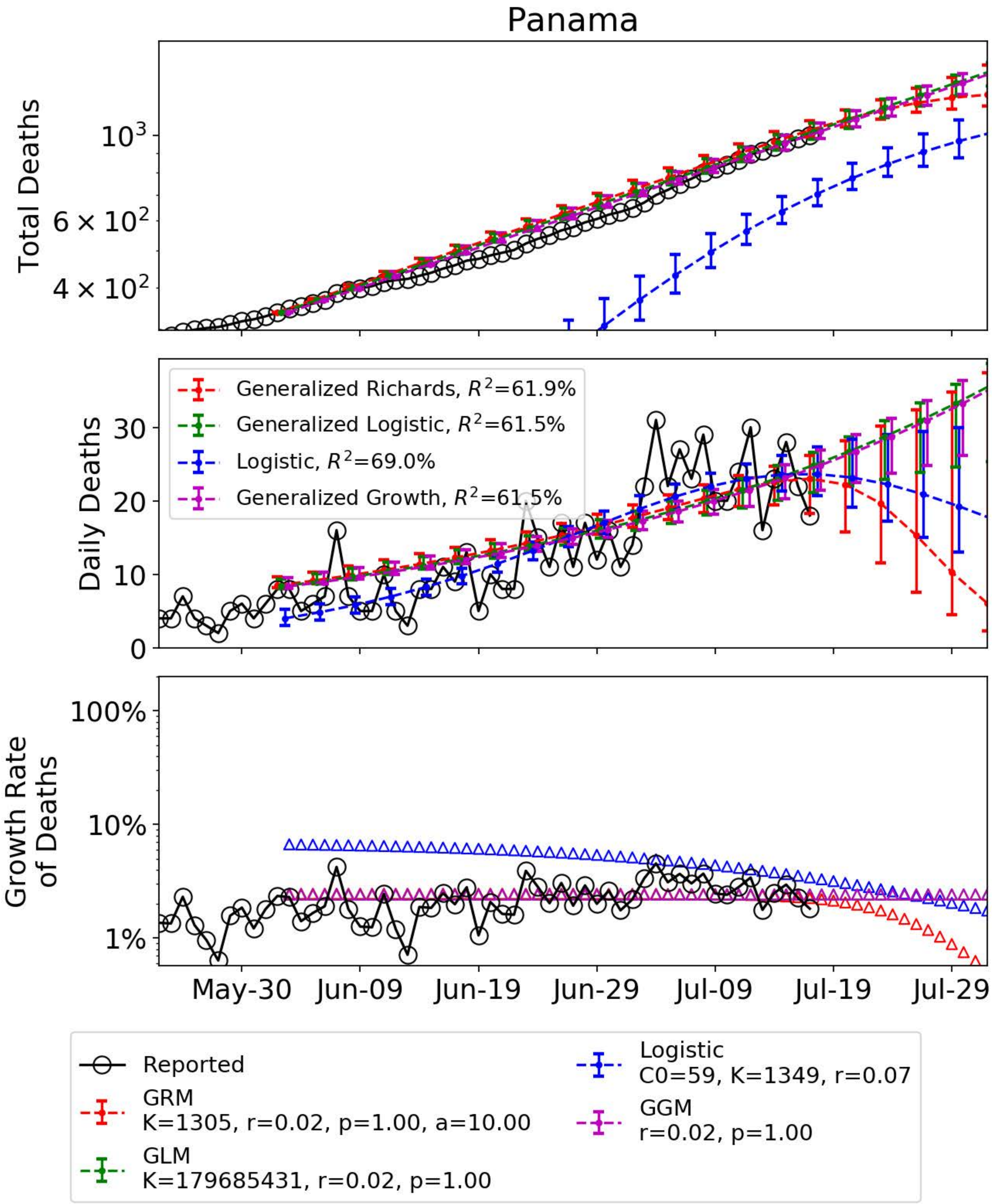
Panama
Total Deths
10^3
6×10^2
4×10^2
Daily Deaths
Generalized Richards, R^2=61.9%
Generalized Logistic, R^2=61.5%
Logistic, R^2=69.0%
Generalized Growth, R^2=61.5%
Growth Rate of Deaths
100%
10%
1%
May-30
Jun-09
Jun-19
Jun-29
Jul-09
Jul-19
Jul-29
Reported
GRM
K=1305, r=0.02, p=1.00, a=10.00
GLM
K=179685431, r=0.02, p=1.00
Logistic
C0=59, K=1349, r=0.07
GGM
r=0.02, p=1.00

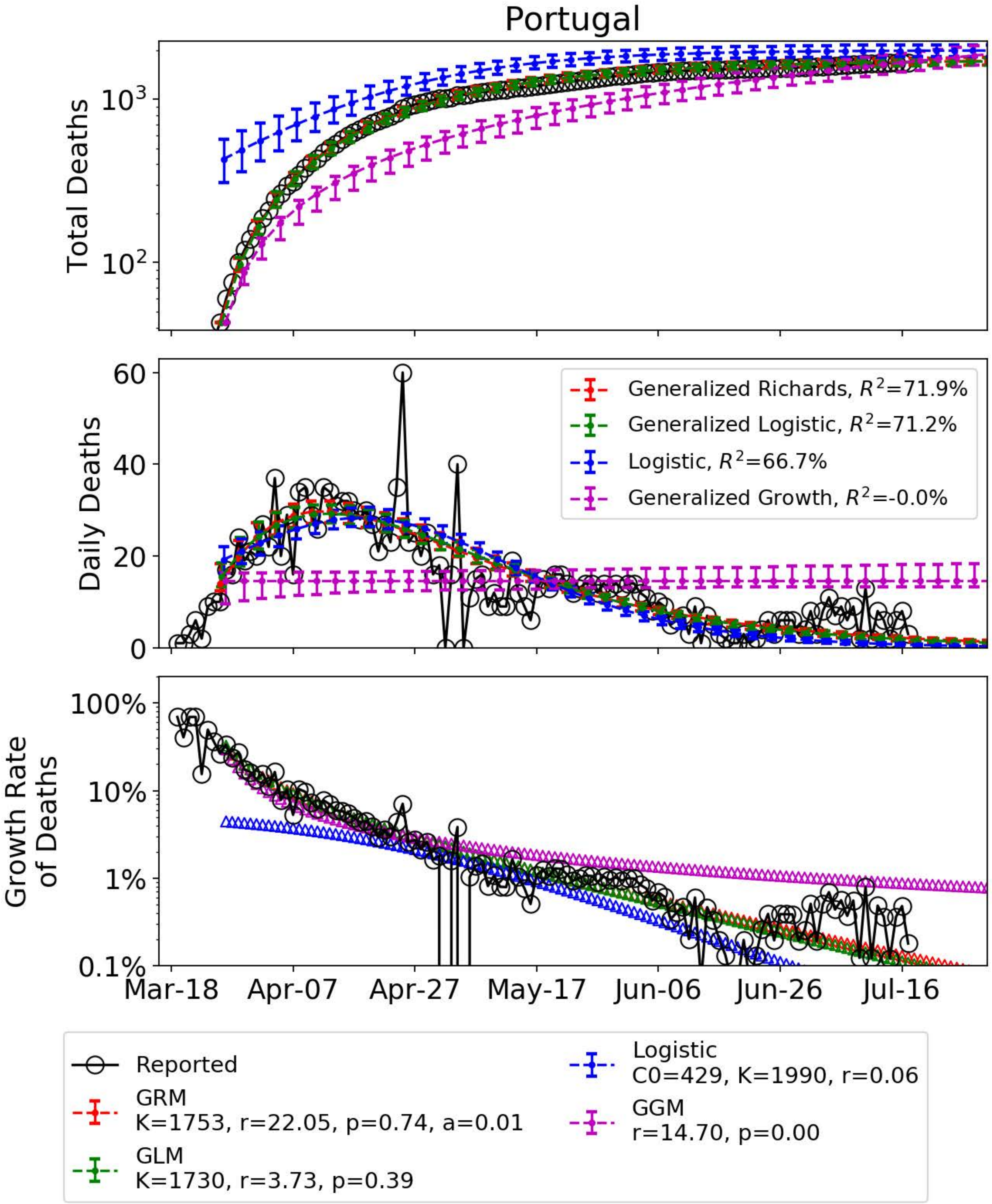

Portugal
Total Deaths
Daily Deaths
Growth Rate of Deaths
Generalized Richards, R^2=71.9%
Generalized Logistic, R^2=71.2%
Logistic, R^2=66.7%
Generalized Growth, R^2=-0.0%
Mar-18
Apr-07
Apr-27
May-17
Jun-06
Jun-26
Jul-16
Reported
GRM
K=1753, r=22.05, p=0.74, a=0.01
GLM
K=1730, r=3.73, p=0.39
Logistic
C0=429, K=1990, r=0.06
GGM
r=14.70, p=0.00

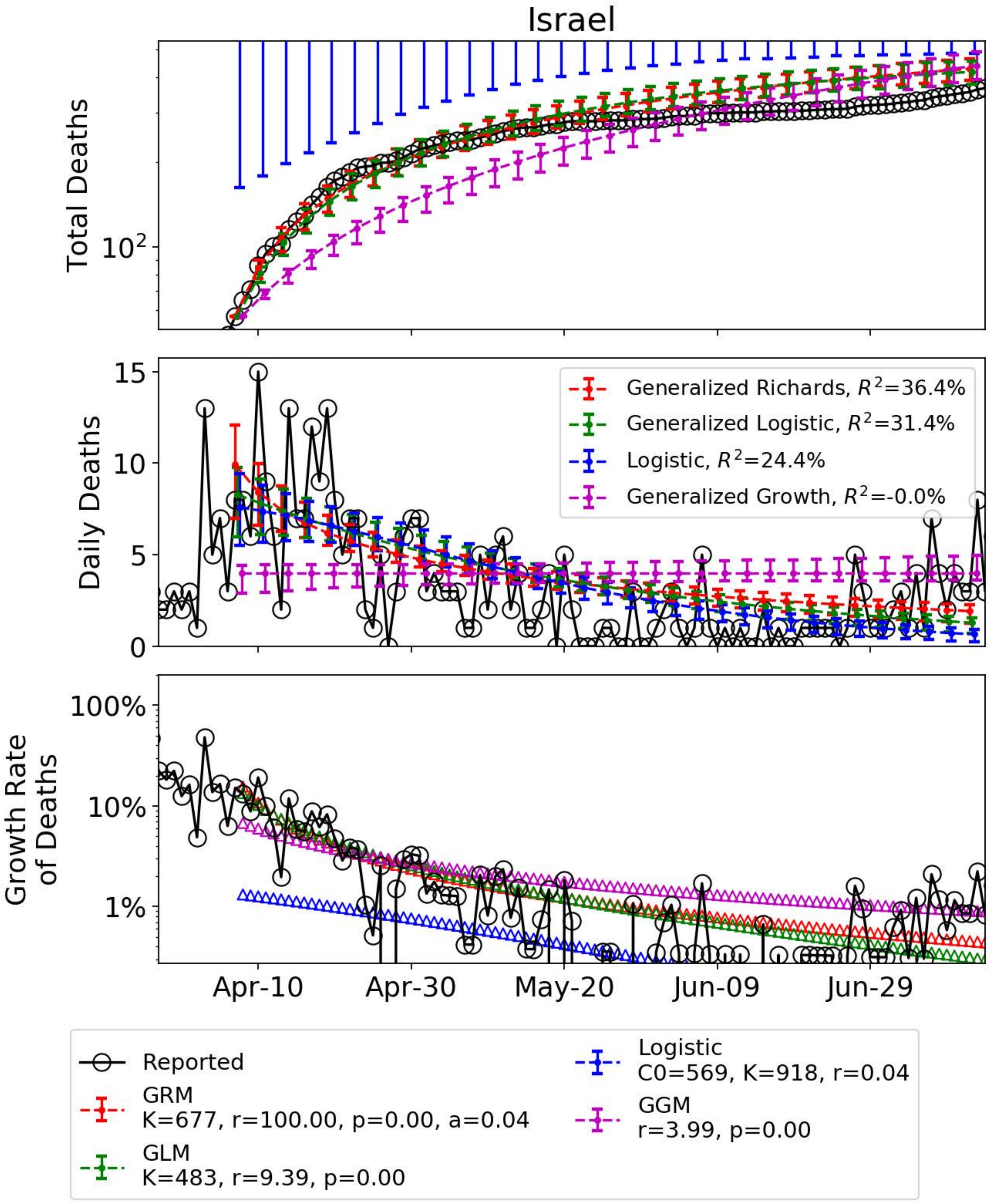

Israel
Total Deaths
10^2
Daily Deaths
15
10
5
0
Generalized Richards, R^2=36.4%
Generalized Logistic, R^2=31.4%
Logistic, R^2=24.4%
Generalized Growth, R^2=-0.0%
Growth Rate of Deaths
100%
10%
1%
Apr-10
Apr-30
May-20
Jun-09
Jun-29
Reported
GRM
K=677, r=100.00, p=0.00, a=0.04
GLM
K=483, r=9.39, p=0.00
Logistic
C0=569, K=918, r=0.04
GGM
r=3.99, p=0.00

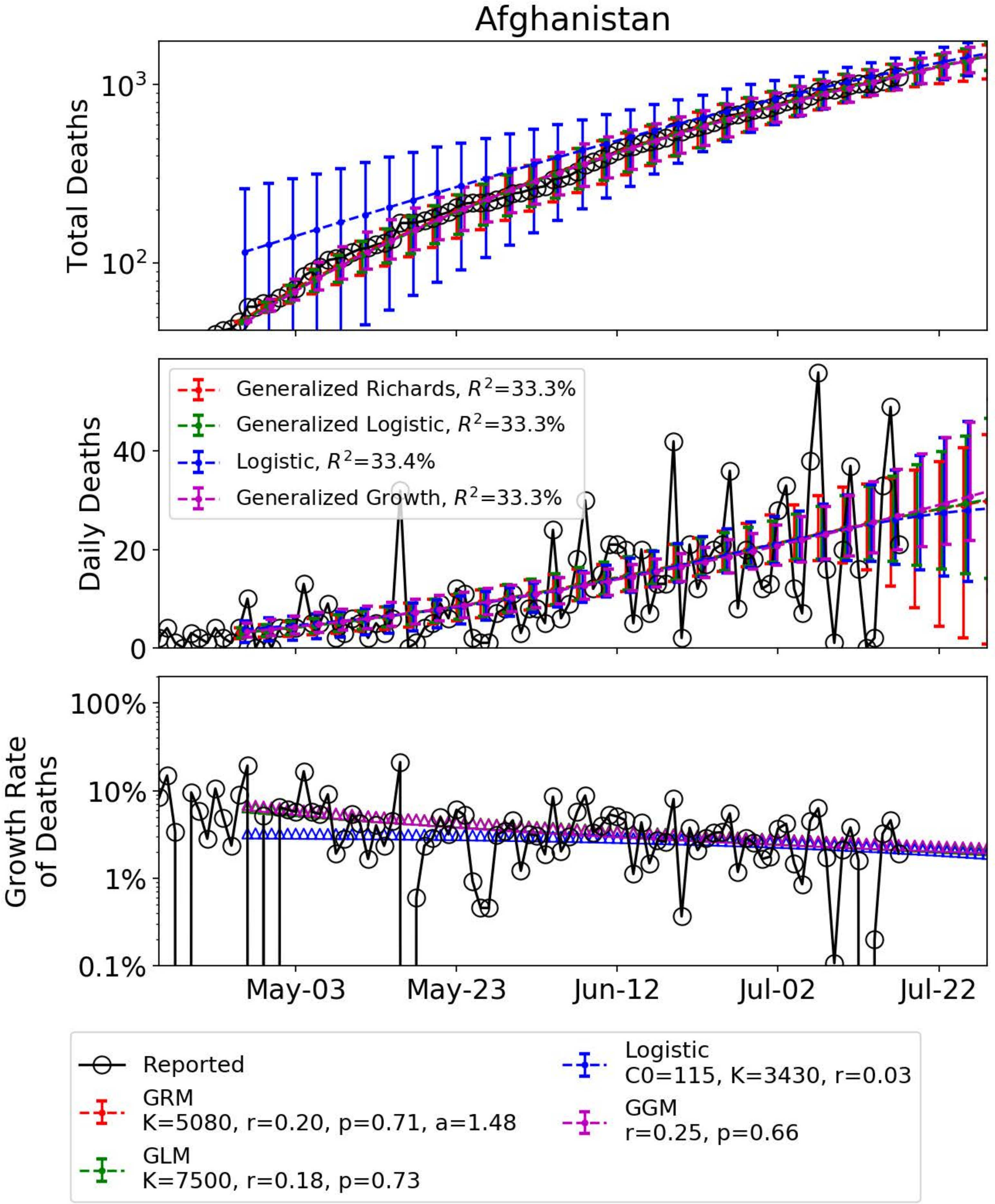

Afghanistan
Total Deaths
10^3
10^2
Daily Deaths
40
20
0
Generalized Richards, R^2=33.3%
Generalized Logistic, R^2=33.3%
Logistic, R^2=33.4%
Generalized Growth, R^2=33.3%
Growth Rate of Deaths
100%
10%
1%
0.1%
May-03
May-23
Jun-12
Jul-02
Jul-22
Reported
GRM
K=5080, r=0.20, p=0.71, a=1.48
GLM
K=7500, r=0.18, p=0.73
Logistic
C0=115, K=3430, r=0.03
GGM
r=0.25, p=0.66

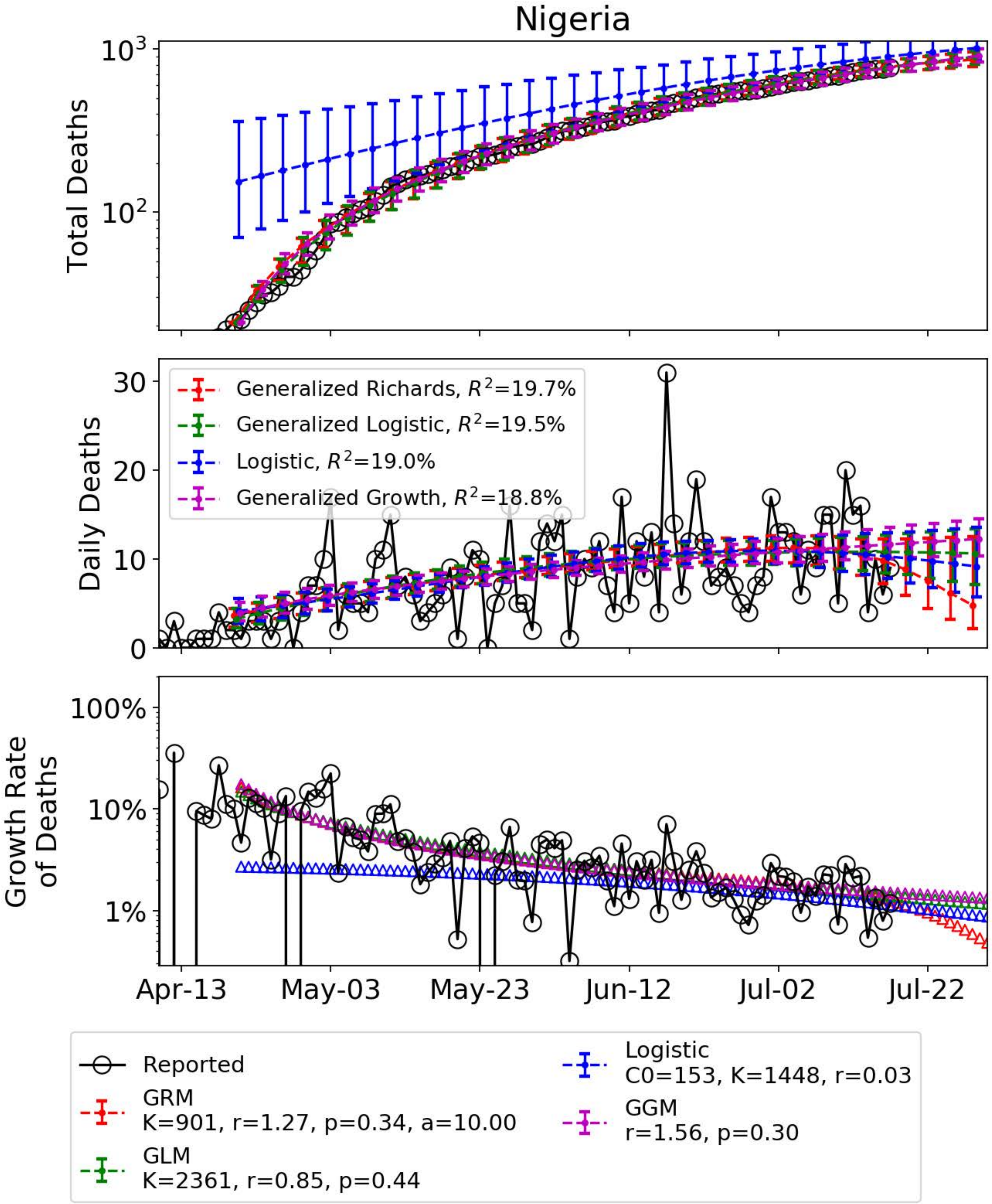

Nigeria
Total Deaths
10^3
10^2
Daily Deaths
30
20
10
0
Generalized Richards, R^2=19.7%
Generalized Logistic, R^2=19.5%
Logistic, R^2=19.0%
Generalized Growth, R^2=18.8%
Growth Rate of Deaths
100%
10%
1%
Apr-13
May-03
May-23
Jun-12
Jul-02
Jul-22
Reported
GRM
K=901, r=1.27, p=0.34, a=10.00
GLM
K=2361, r=0.85, p=0.44
Logistic
C0=153, K=1448, r=0.03
GGM
r=1.56, p=0.30

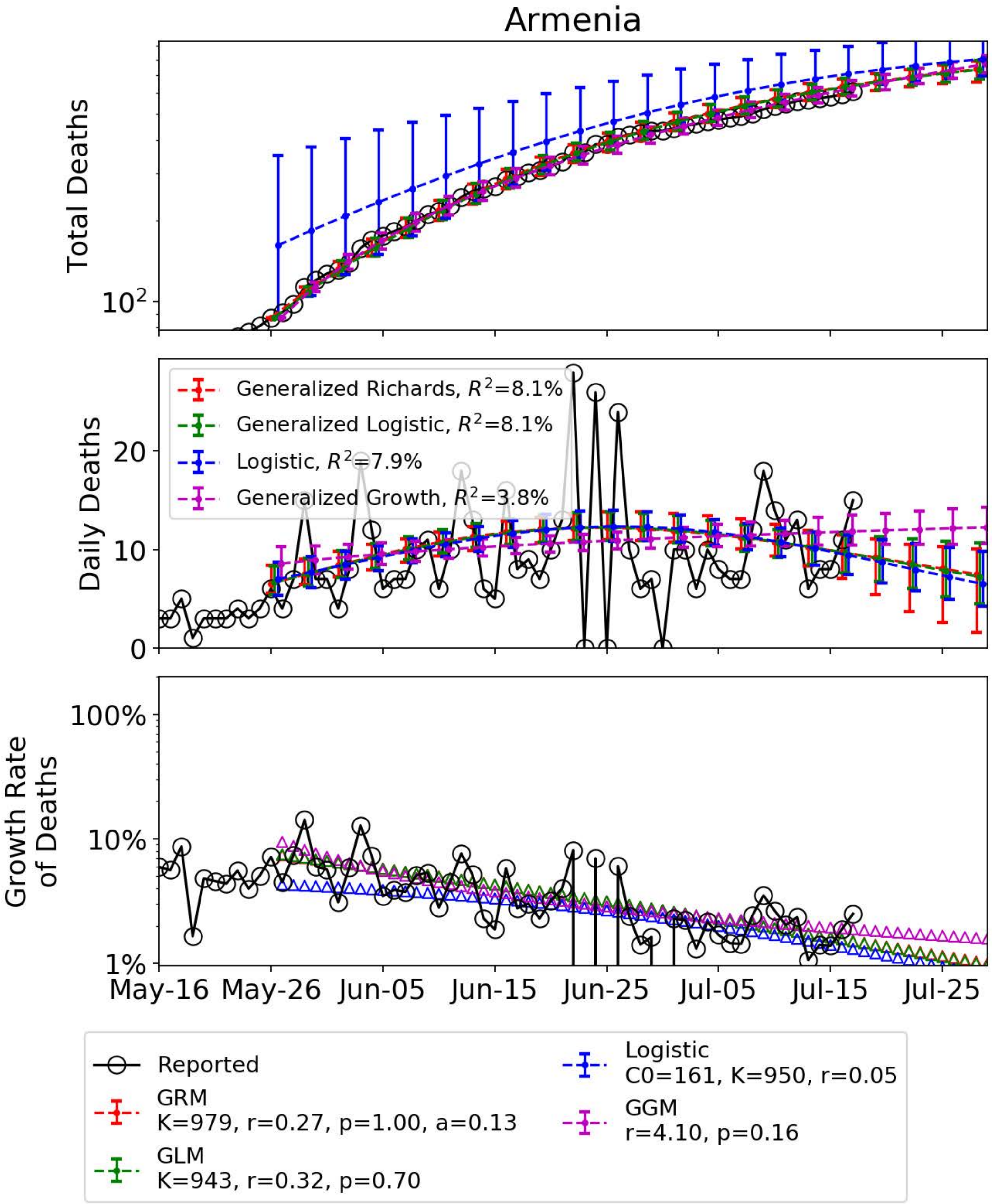
Armenia
Total Deaths
10^2
Daily Deaths
Generalized Richards, R^2=8.1%
Generalized Logistic, R^2=8.1%
Logistic, R^2=7.9%
Generalized Growth, R^2=3.8%
20
10
0
Growth Rate of Deaths
100%
10%
1%
May-16
May-26
Jun-05
Jun-15
Jun-25
Jul-05
Jul-15
Jul-25
Reported
GRM
K=979, r=0.27, p=1.00, a=0.13
GLM
K=943, r=0.32, p=0.70
Logistic
C0=161, K=950, r=0.05
GGM
r=4.10, p=0.16

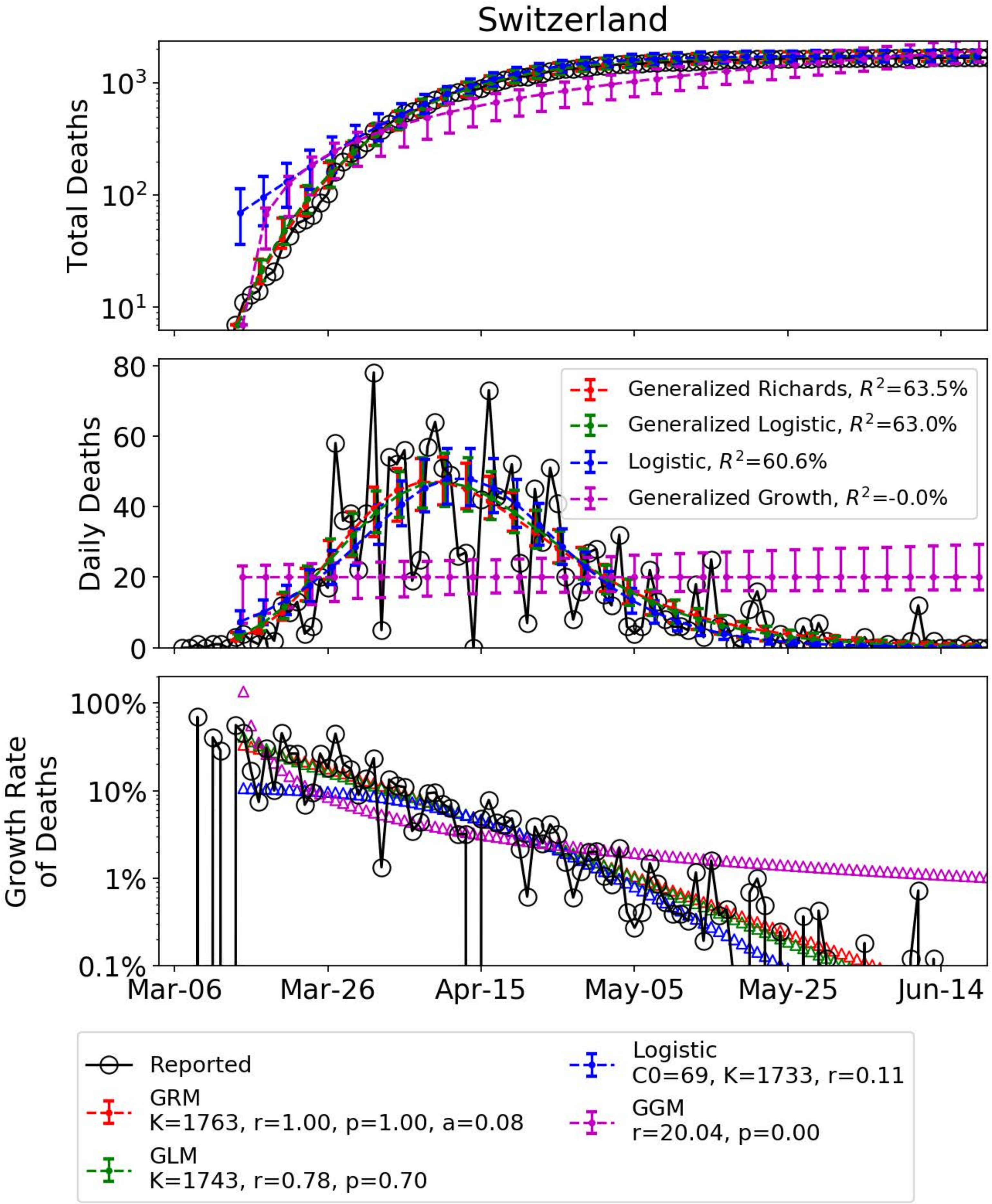

Switzerland
Total Deaths
Daily Deaths
Growth Rate of Deaths
Generalized Richards, R^2=63.5%
Generalized Logistic, R^2=63.0%
Logistic, R^2=60.6%
Generalized Growth, R^2=-0.0%
Mar-06
Mar-26
Apr-15
May-05
May-25
Jun-14
Reported
GRM
K=1763, r=1.00, p=1.00, a=0.08
GLM
K=1743, r=0.78, p=0.70
Logistic
C0=69, K=1733, r=0.11
GGM
r=20.04, p=0.00

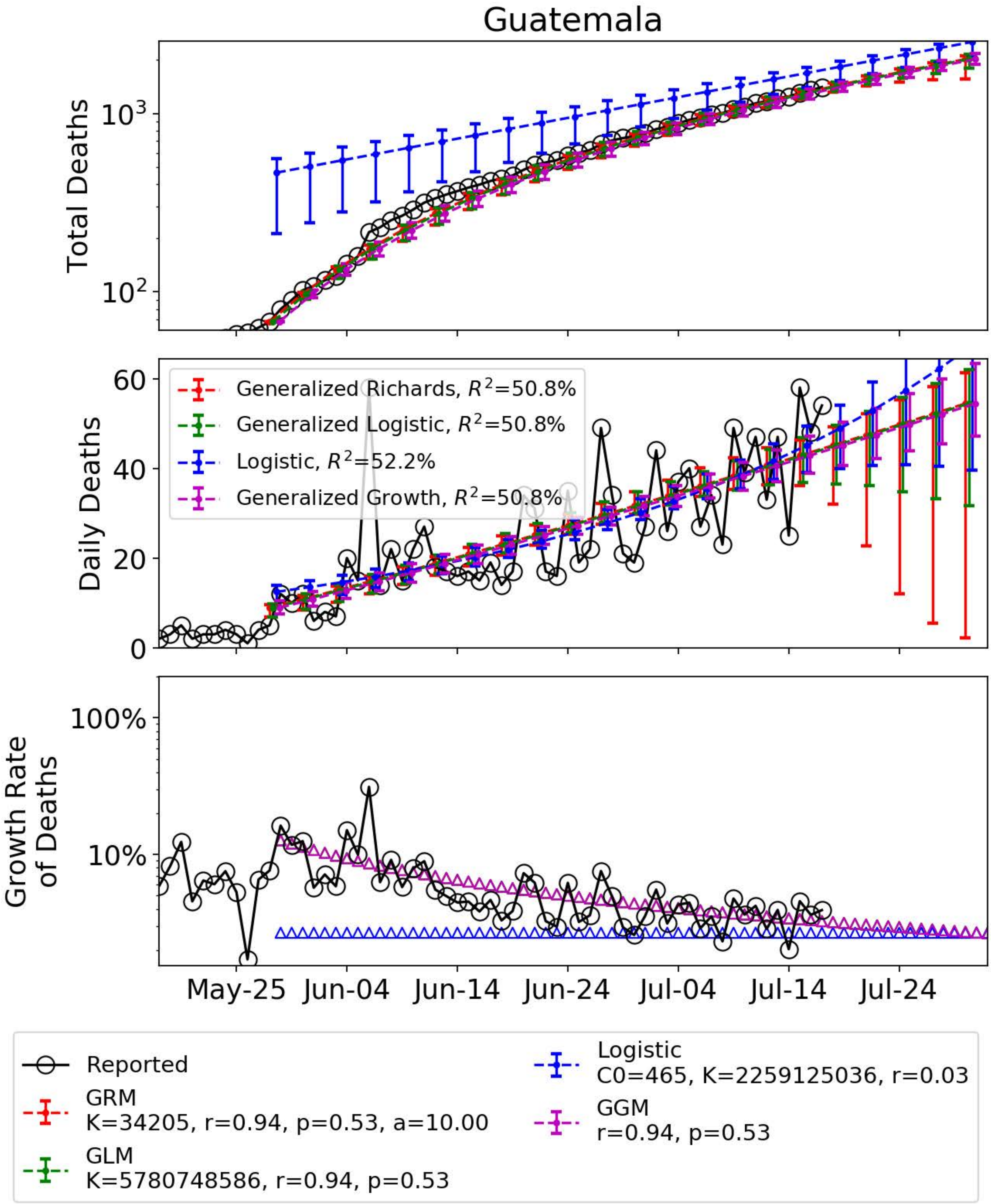

Guatemala
Total Deaths
10^3
10^2
Daily Deaths
60
40
20
0
Generalized Richards, R^2=50.8%
Generalized Logistic, R^2=50.8%
Logistic, R^2=52.2%
Generalized Growth, R^2=50.8%
Growth Rate of Deaths
100%
10%
May-25
Jun-04
Jun-14
Jun-24
Jul-04
Jul-14
Jul-24
Reported
GRM
K=34205, r=0.94, p=0.53, a=10.00
GLM
K=5780748586, r=0.94, p=0.53
Logistic
C0=465, K=2259125036, r=0.03
GGM
r=0.94, p=0.53

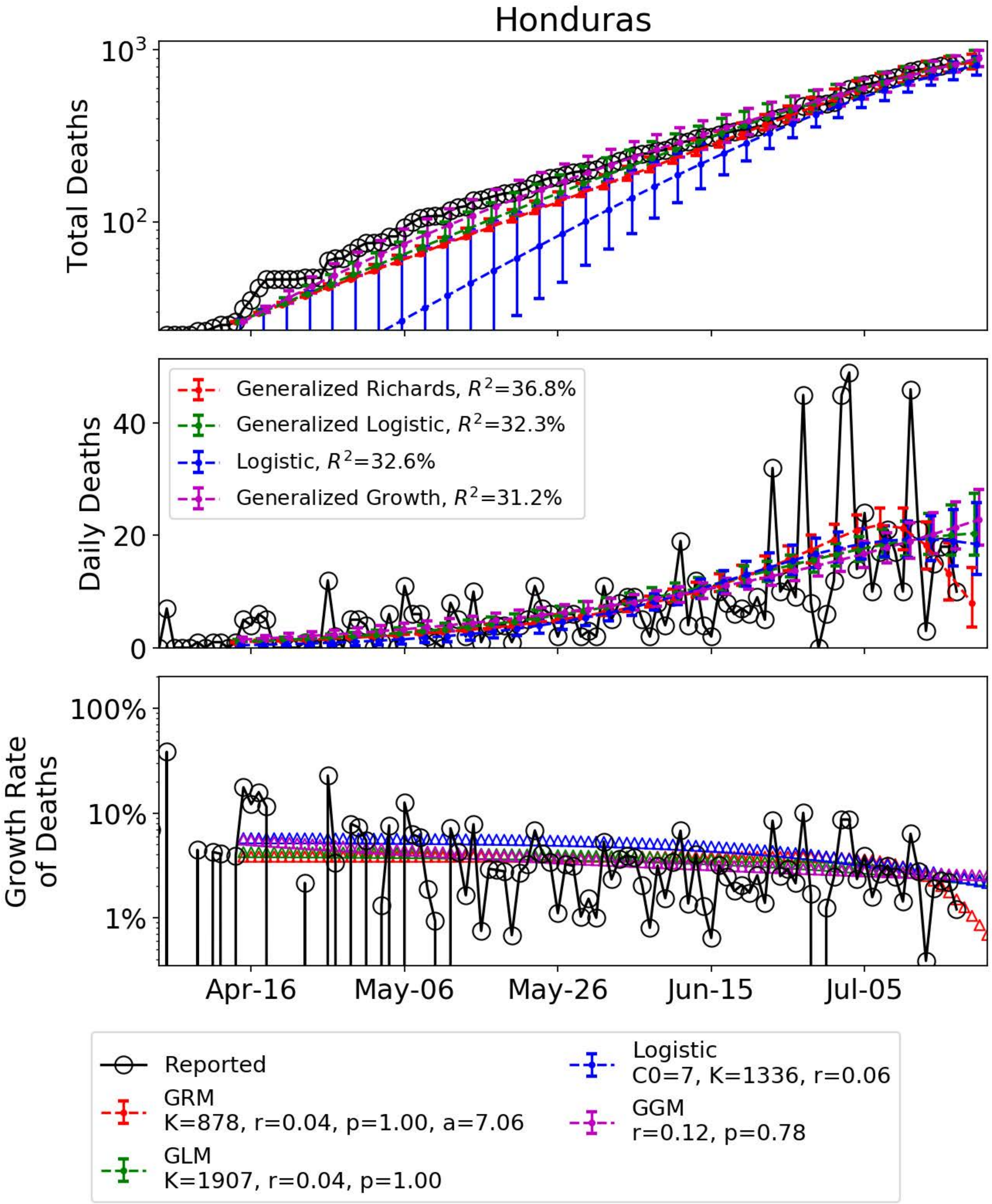

Honduras
Total Deaths
Daily Deaths
Growth Rate of Deaths
Generalized Richards, R^2=36.8%
Generalized Logistic, R^2=32.3%
Logistic, R^2=32.6%
Generalized Growth, R^2=31.2%
Apr-16
May-06
May-26
Jun-15
Jul-05
Reported
GRM
K=878, r=0.04, p=1.00, a=7.06
GLM
K=1907, r=0.04, p=1.00
Logistic
C0=7, K=1336, r=0.06
GGM
r=0.12, p=0.78

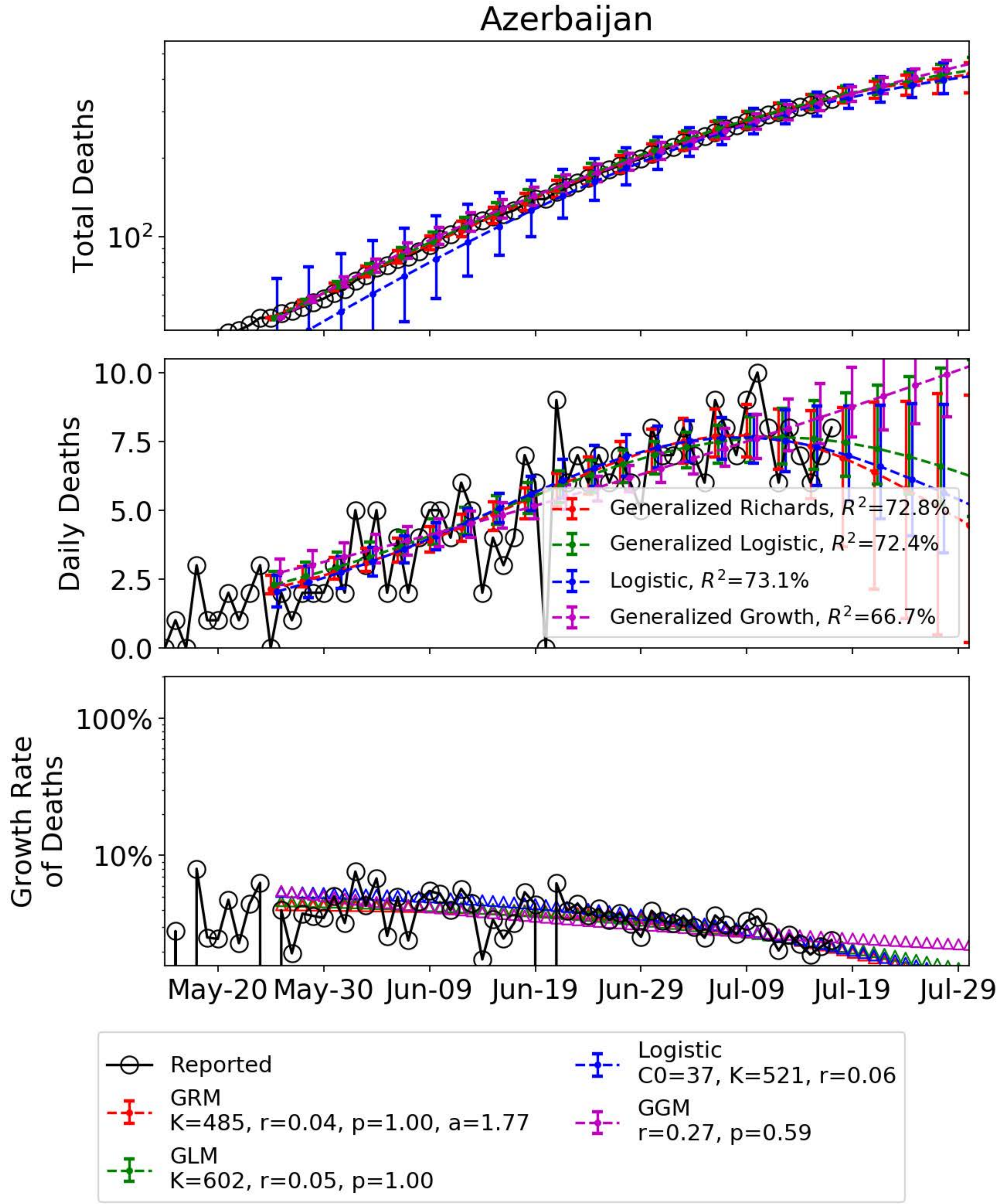

Azerbaijan
Total Deaths
10^2
Daily Deaths
10.0
7.5
5.0
2.5
0.0
Generalized Richards, R^2=72.8%
Generalized Logistic, R^2=72.4%
Logistic, R^2=73.1%
Generalized Growth, R^2=66.7%
Growth Rate of Deaths
100%
10%
May-20
May-30
Jun-09
Jun-19
Jun-29
Jul-09
Jul-19
Jul-29
Reported
GRM
K=485, r=0.04, p=1.00, a=1.77
GLM
K=602, r=0.05, p=1.00
Logistic
C0=37, K=521, r=0.06
GGM
r=0.27, p=0.59

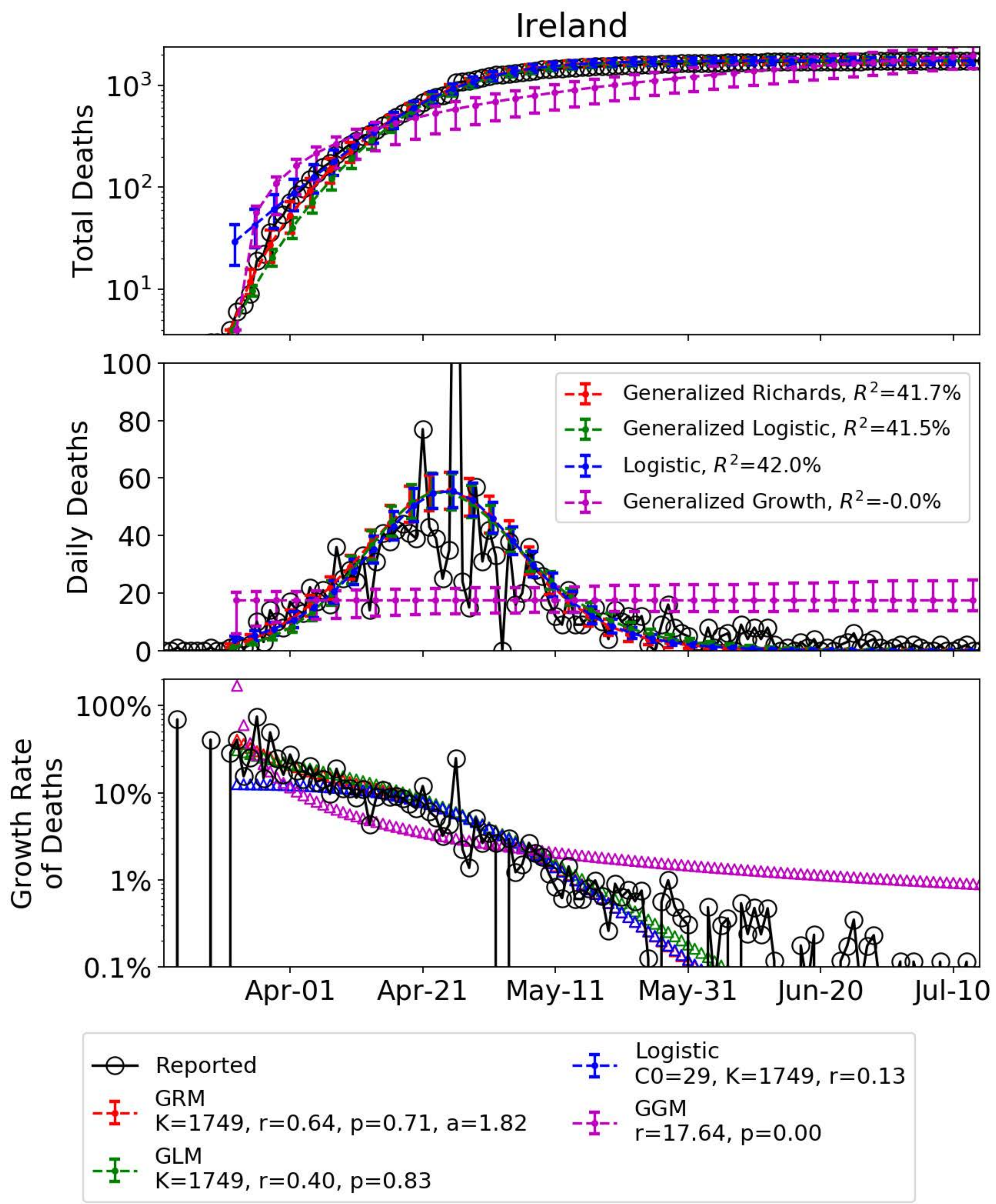

Ireland
Total Deaths
Daily Deaths
Growth Rate of Deaths
Generalized Richards, R^2=41.7%
Generalized Logistic, R^2=41.5%
Logistic, R^2=42.0%
Generalized Growth, R^2=-0.0%
Apr-01
Apr-21
May-11
May-31
Jun-20
Jul-10
Reported
GRM
K=1749, r=0.64, p=0.71, a=1.82
GLM
K=1749, r=0.40, p=0.83
Logistic
C0=29, K=1749, r=0.13
GGM
r=17.64, p=0.00

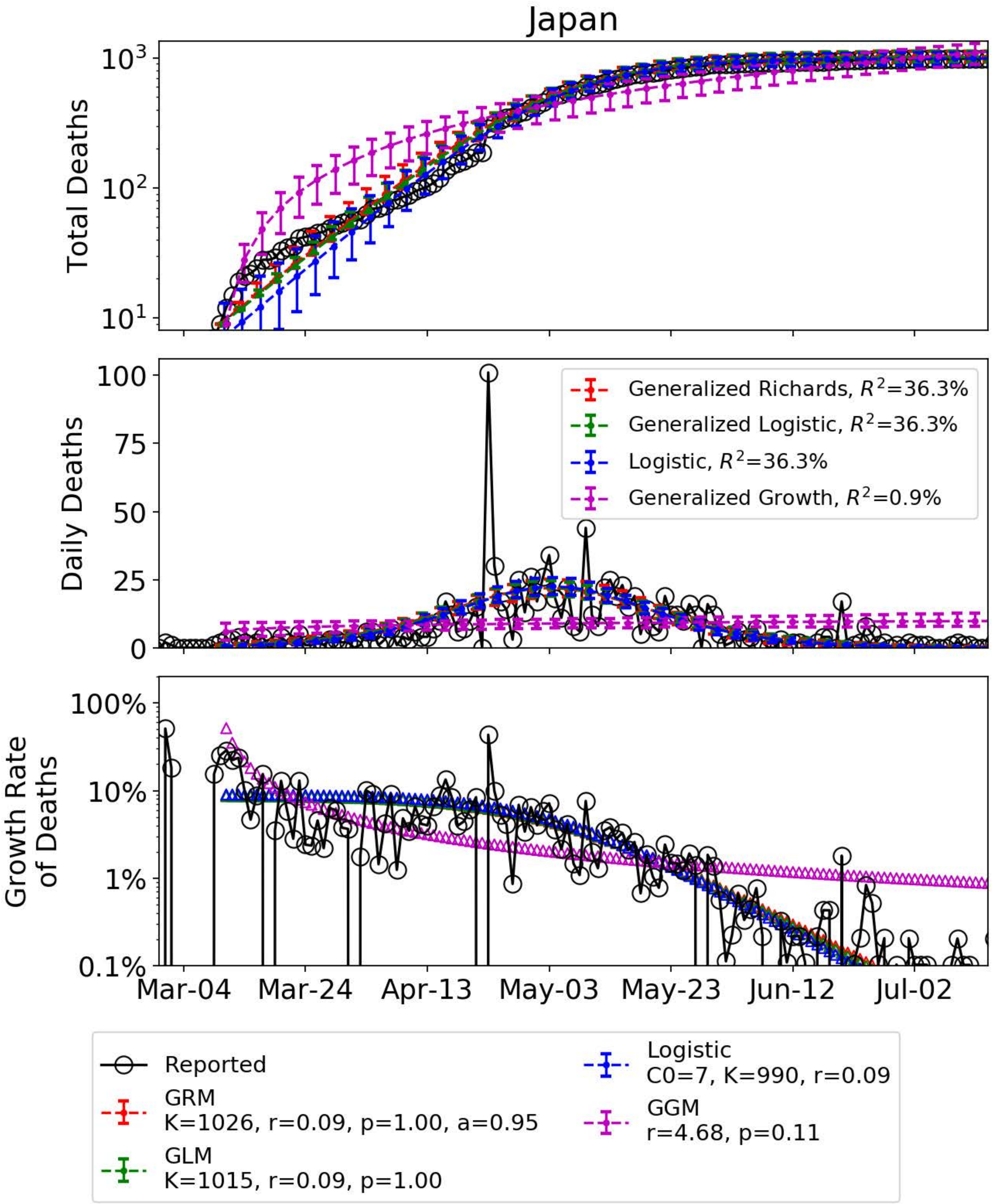

Japan
Total Deaths
Daily Deaths
Growth Rate of Deaths
Generalized Richards, R^2=36.3%
Generalized Logistic, R^2=36.3%
Logistic, R^2=36.3%
Generalized Growth, R^2=0.9%
Mar-04
Mar-24
Apr-13
May-03
May-23
Jun-12
Jul-02
Reported
GRM
K=1026, r=0.09, p=1.00, a=0.95
GLM
K=1015, r=0.09, p=1.00
Logistic
C0=7, K=990, r=0.09
GGM
r=4.68, p=0.11

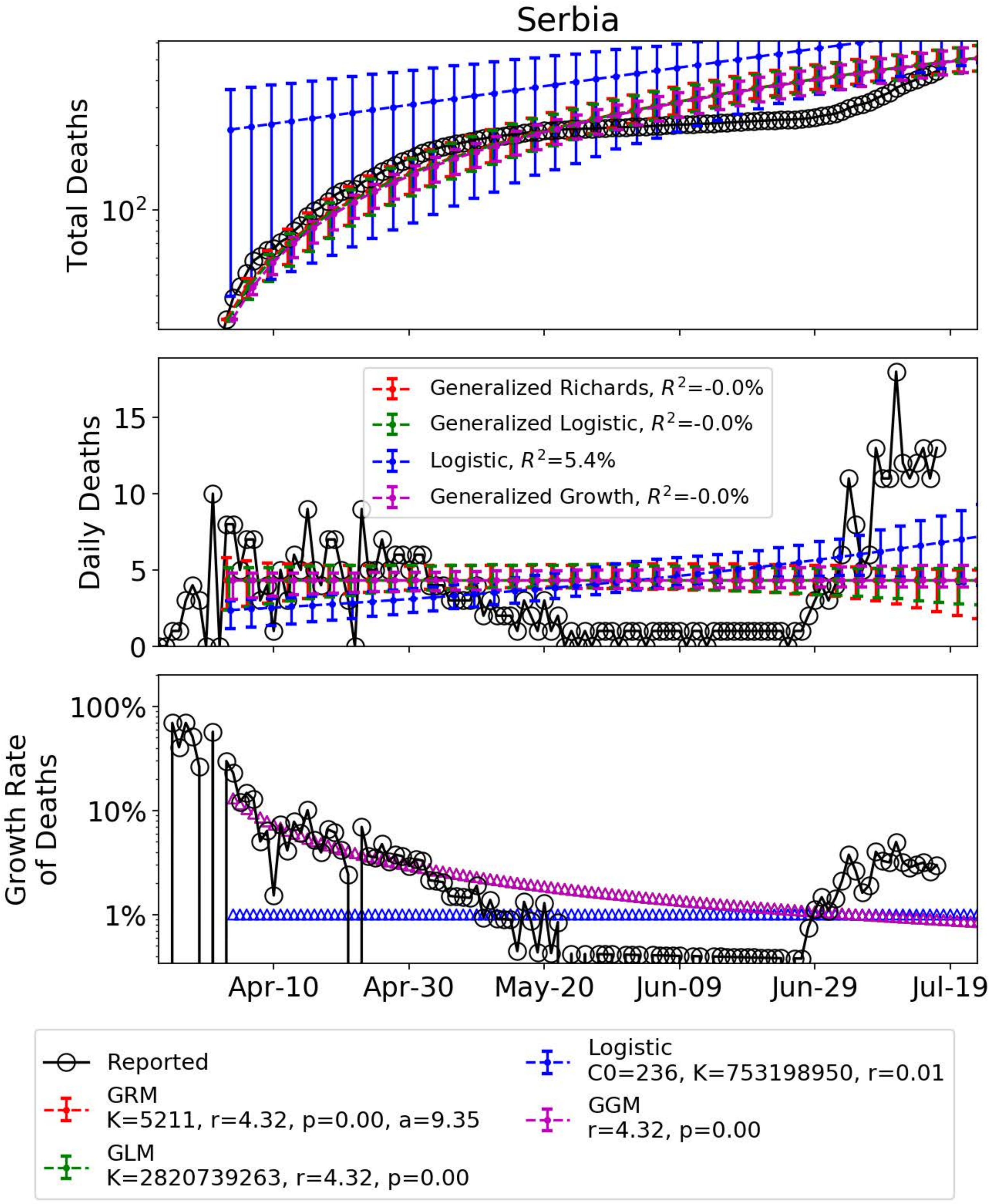
Serbia
Total Deaths
10^2
Daily Deaths
0
5
10
15
Generalized Richards, R^2=-0.0%
Generalized Logistic, R^2=-0.0%
Logistic, R^2=5.4%
Generalized Growth, R^2=-0.0%
Growth Rate of Deaths
100%
10%
1%
Apr-10
Apr-30
May-20
Jun-09
Jun-29
Jul-19
Reported
GRM
K=5211, r=4.32, p=0.00, a=9.35
GLM
K=2820739263, r=4.32, p=0.00
Logistic
C0=236, K=753198950, r=0.01
GGM
r=4.32, p=0.00

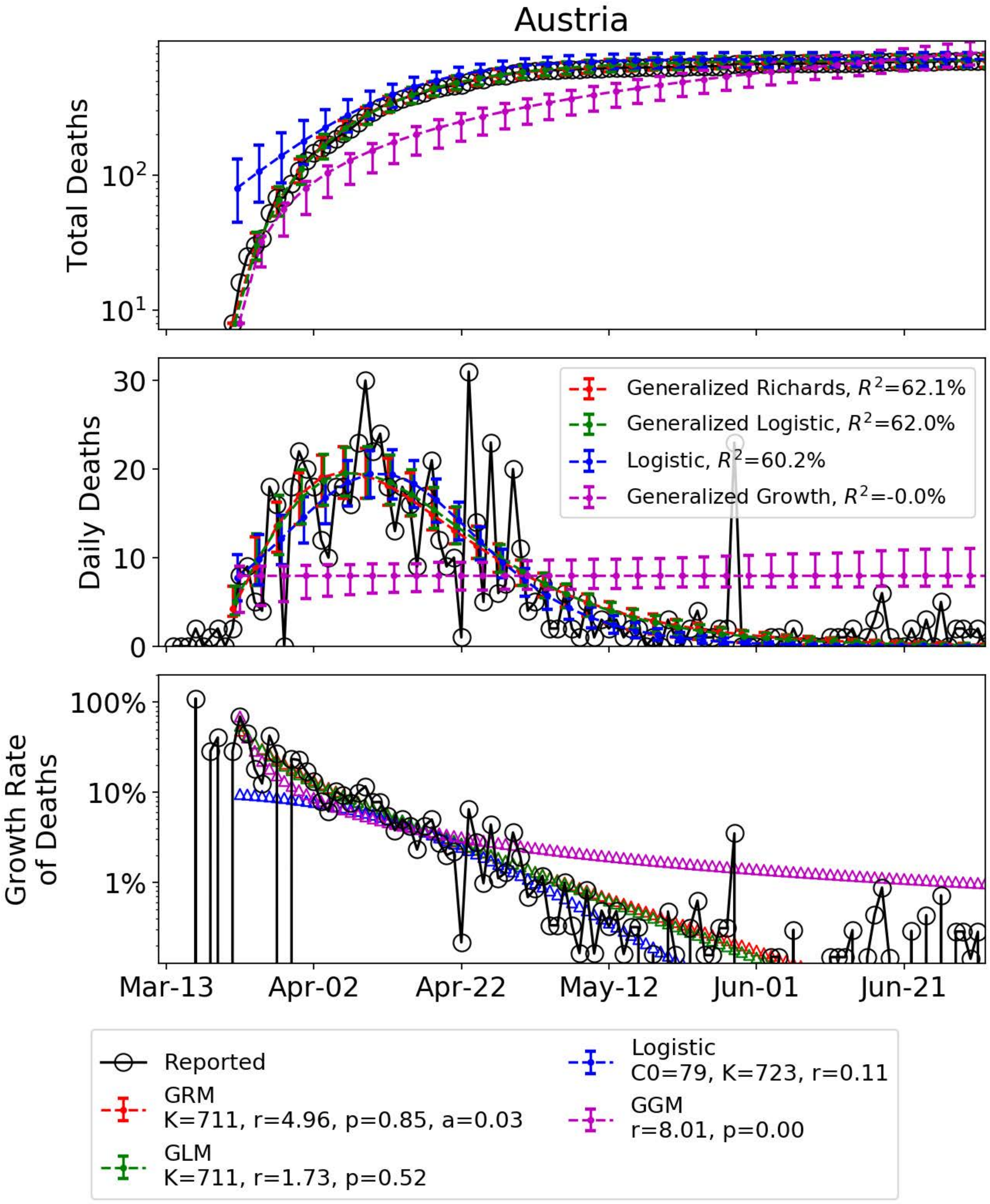

Austria
Total Deaths
10^2
10^1
Daily Deaths
30
20
10
0
Generalized Richards, R^2=62.1%
Generalized Logistic, R^2=62.0%
Logistic, R^2=60.2%
Generalized Growth, R^2=-0.0%
Growth Rate of Deaths
100%
10%
1%
Mar-13
Apr-02
Apr-22
May-12
Jun-01
Jun-21
Reported
GRM
K=711, r=4.96, p=0.85, a=0.03
GLM
K=711, r=1.73, p=0.52
Logistic
C0=79, K=723, r=0.11
GGM
r=8.01, p=0.00
